# Effects of virtual reality-based interventions on cognitive function, emotional state, and quality of life in patients with mild cognitive impairment: a meta-analysis

**DOI:** 10.3389/fneur.2025.1496382

**Published:** 2025-04-02

**Authors:** Xiaohan Li, Yuting Zhang, Lifeng Tang, Lin Ye, Min Tang

**Affiliations:** ^1^Neurological Rehabilitation Department, Ningbo Rehabilitation Hospital, Ningbo, China; ^2^Faculty of Marine, Ningbo University, Ningbo, China; ^3^Faculty of Rehabilitation, Gannan Medical University, Ganzhou, China

**Keywords:** virtual reality, mild cognitive impairment, cognitive function, emotional state, quality of life

## Abstract

**Objectives:**

This meta-analysis aims to systematically evaluate the effects of virtual reality (VR)-based interventions on cognitive function, emotional state, and quality of life in patients with mild cognitive impairment (MCI).

**Methods:**

A comprehensive literature search was conducted using five databases from their inception to June 2024. The inclusion criteria focused on randomized controlled trials (RCTs) that examined VR-based interventions in adults aged 60 or older diagnosed with MCI. The primary outcome was cognitive function, while secondary outcomes included emotional state, quality of life, and dynamic balance. To investigate potential sources of heterogeneity, subgroup analyses and meta-regression were conducted. Subgroup analyses were stratified by VR parameters (immersion level, duration, session, and frequency) and demographic factors (geographic region, education level, and male proportion). Publication bias was assessed using funnel plots and Egger’s regression test. A “trim and fill” method was employed to adjust for any detected publication bias. The certainty of the evidence was evaluated using the Grading of Recommendations Assessment, Development, and Evaluation (GRADE) framework with the GRADEpro GDT software.

**Results:**

A total of 30 RCTs involving 1,365 participants from 9 countries across 4 continents were included. The meta-analysis revealed that VR-based interventions significantly improved global cognition, as assessed by the Montreal Cognitive Assessment (MoCA; SMD = 0.82, 95% CI: 0.27 to 1.38, *p* = 0.003, GRADE: moderate) and the Mini-Mental State Examination (MMSE; SMD = 0.83, 95% CI: 0.40 to 1.26, *p* = 0.0001, GRADE: low). Additionally, VR interventions enhanced attention, as measured by the Digit Span Backward (DSB; SMD = 0.61, 95% CI: 0.21 to 1.02, *p* = 0.003, GRADE: low) and Digit Span Forward (DSF; SMD = 0.89, 95% CI: 0.34 to 1.45, *p* = 0.002, GRADE: low). Improvements were also observed in quality of life, as indicated by scores on the Instrumental Activities of Daily Living (IADL; SMD = 0.22, 95% CI: 0.00 to 0.45, *p* = 0.049, GRADE: moderate). However, no significant effects were found for executive function, memory, verbal fluency, visual abilities, emotional status, or dynamic balance (*p* > 0.05). Subgroup analysis revealed that VR interventions were more effective when using semi-immersive VR, with session durations of ≤60 min and a frequency of more than twice per week. Participants from Asia and Europe demonstrated better outcomes, and a lower proportion of male participants (≤ 40%) was also associated with improvements in targeted cognitive domains.

**Conclusion:**

The findings indicate that VR interventions can significantly improve global cognition, attention, and quality of life in individuals with MCI. Subgroup analyses further revealed that optimal cognitive outcomes were associated with semi-immersive VR, session durations of ≤60 min, intervention frequencies exceeding twice per week, studies conducted in Asia and Europe, and participant groups with a male proportion of ≤40%. Moreover, the study provides valuable insights into secondary outcomes, suggesting that VR interventions may positively impact emotional state and dynamic balance when appropriately tailored to factors such as immersion level, duration, frequency, and other relevant parameters.

## 1 Introduction

Mild cognitive impairment (MCI) is a clinical condition that represents a transitional stage between the cognitive decline associated with normal aging and the more severe impairment seen in dementia, particularly Alzheimer’s disease (AD) (1, 2). Individuals diagnosed with MCI are at a significantly higher risk of progressing to dementia, with a mean annual conversion rate of approximately 10%, compared to the annual incidence of 1–2% in the general population (3, 4). This critical period offers an opportunity for intervention, making the identification of effective therapeutic strategies to delay or prevent the progression to dementia of utmost importance.

The increasing prevalence of MCI, driven by the aging global population, has intensified the need for innovative interventions that not only address cognitive decline but also improve emotional well-being and overall quality of life. Traditional cognitive rehabilitation methods, including memory training (5), cognitive exercises (6), and pharmacotherapy (7), have shown some efficacy but often suffer from limitations such as low patient engagement and adherence. These limitations have spurred interest in alternative and more engaging therapeutic approaches.

Virtual reality (VR) technology has emerged as a promising tool in the field of cognitive rehabilitation due to its unique ability to create immersive and interactive environments (8). VR allows for the simulation of real-world scenarios in a controlled and customizable manner, making it an ideal platform for cognitive training (9). Unlike traditional cognitive exercises, VR can engage multiple senses simultaneously, potentially leading to more robust cognitive benefits (8). Moreover, the interactive nature of VR can enhance patient motivation and adherence to therapy (9, 10), which are critical factors in the success of any long-term intervention.

In recent years, a growing body of research has explored the application of VR-based interventions for cognitive rehabilitation in various populations, including stroke, Parkinson, MCI, and dementia, and et. For instance, a recent meta-analysis demonstrated that VR has significant beneficial effects on cognitive function in individuals who have sustained a stroke (11). Similarly, Lei et al. reported that VR not only achieves similar effects to conventional rehabilitation training but also improves gait and balance performance in patients with Parkinson’s disease (12). Another review also found that computerized cognitive training or VR technology could improve cognition, executive functions, and attention of MCI or AD patients to some extent (13).

However, much of the existing literature tends to combine MCI and dementia in analyses, which may obscure important differences in intervention effectiveness between these groups (1, 14–17). Given that MCI and dementia represent distinct stages of cognitive decline, it is critical to separately analyze the effects of VR-based interventions on individuals with MCI. Such an approach allows for a more precise understanding of how VR can be utilized to target the specific needs of this population and potentially prevent the progression to dementia.

This meta-analysis aims to address this gap by systematically evaluating the effects of VR-based interventions on cognitive function, emotional state, and quality of life in patients with MCI. By focusing specifically on MCI, this study seeks to provide a clearer picture of the therapeutic potential of VR for this population and offer evidence-based recommendations for its application in clinical practice.

## 2 Materials and methods

### 2.1 Registration

The protocol was prospectively registered on the PROSPERO International Prospective Register for Systematic Reviews website (Registration #: CRD42023489464) in December 2023. Design and reporting of this review have followed “Preferred Reporting Items for Systematic Reviews and Meta-Analyses” (PRISMA) statement (Supplementary Table 1) (18).

### 2.2 Literature search strategy

English language articles were retrieved by title and abstract from the earliest record up to June 2024 from PubMed, Embase, Elsevier, Web of Science, and SciELO by two independent authors (X.L. and Y.Z.). The search strategy (based on Medical Subject Headings) combined the following terms: “Mild Cognitive Impairment”; “MCI”; “Virtual Reality”; “VR”; “Cognitive Function” (the full search strategy is reported in Supplementary Table 2). All literature was imported into Endnote X9 (Thomson Reuters, Carlsbad, CA, USA), which also removed duplications. Two reviewers (L.T. and L.Y.) screened all titles and abstracts. Once abstracts suggested that studies were potentially suitable, the full-text versions were screened and then included in the review if they fulfilled the selection criteria. A third reviewer (M.T.) was consulted in cases of disagreement.

### 2.3 Selection criteria

#### 2.3.1 Inclusion criteria

The inclusion criteria were defined with the PICOS approach:

(a)   P (population): all populations were aged more than 60 years, with a diagnosis of MCI or cognitive impairment. Diagnostic criteria for MCI patients included Mini-Mental State Examination (MMSE) (11–26 score), Montreal Cognitive Assessment (MoCA) (< 26 score), Monongahela-Youghiogheny Healthy Aging Team assessment, subjective cognitive decline and diagnosis by doctors.
(b)   I (intervention): The experimental group (EG) received VR-based rehabilitation training. The VR intervention should be the use of interactive simulations created with computer hardware and software to present users with a virtual figure to engage in environments that appear and feel similar to real world objects and events (1).
(c)   C (comparison): The control group (CG) received no intervention, or received conventional training, or received an alternative intervention such as health education.
(d)   O (outcomes): The primary outcome of this study was cognitive function: (I) Global Cognition: MoCA, MMSE, Symbol Digit Substitution Test (SDST), and Cognitive Failure Questionnaire (CFQ); (II) Execution Cognition: Trail Making Test–Part A (TMT-A) and Trail Making Test–Part B (TMT-B); (III) Attention: Digit Span Backward (DSB) and Digit Span Forward (DSF); (IV) Memory: Rey Auditory Verbal Learning Test-Immediate Recall (RAVLT-IR), Rey Auditory Verbal Learning Test-Delayed Recall (RAVLT-DR), Chinese Version Verbal Learning Test-Immediate Recall (CVVLT-IR), and Chinese Version Verbal Learning Test-Delayed Recall (CVVLT-DR); (V) Verbal Fluency: Animal Word and “ㅅ” Word; (VI) Visual Ability: Wechsler Adult Intelligence Scale-Block Design Test (WAIS-BDT) and Clock Drawing Test (CDT).

Secondary outcomes were (I) Emotional State: Geriatric Depression Scale-15 (GDS-15) and Geriatric Depression Scale-30 (GDS-30); (II) Quality of Life: Instrumental Activity of Daily Living (IADL) and Quality of Life-Alzheimer Disease (QoL-AD); and (III) Dynamic Balance: Timed Up-and-Go Test (TUG) and Berg Balance Scale (BBS).

(e)   S (study design): randomized controlled trials (RCTs).

#### 2.3.2 Exclusion criteria

- Patients with a history of other neurological diseases (e.g., Parkinson’s disease or stroke,) or psychiatric disorders (e.g., anxiety disorders or depressive).
- During the follow-up period, medications for MCI (cholinesterase inhibitors or memantine) were prescribed.
- Studies not published in English.
- Case reports, cross-sectional, retrospective, systematic reviews, editorial letters, or conference abstracts without the full text available.

### 2.4 Data extraction

Two authors (X.L. and Y.Z.) extracted data independently, with any discrepancies discussed until a consensus was reached. Data on study characteristics (author, published year, and country), sample characteristics (male/female size, age, education years, MMSE, MoCA, and IADL score), EG characteristics (immersive level, intervention component, session, frequency, and duration), CG characteristics (intervention component), and outcome characteristics was extracted. If a study reported results for different periods, each of them was treated as a separate trial (19).

### 2.5 Risk of bias and quality assessment

The quality assessment was conducted using the risk of bias tool from RevMan 5.4.1 (The Cochrane Collaboration in Oxford, England) (20). This tool evaluates seven aspects: random sequence generation, allocation concealment, blinding of participants and personnel, blinding of outcome assessment, incomplete outcome data, selective reporting, and other biases. Each aspect was rated by the researchers as high risk (−), low risk (+), or uncertain risk (?). In cases of disagreement on the ratings, a consultation process was implemented to reach a consensus.

### 2.6 Data synthesis and analysis

The Cochrane systematic review software RevMan 5.4.1 (The Cochrane Collaboration in Oxford, England) was used to create forest plots with 95% confidence intervals (CI). In order to avoid the impression of differences between studies, the standardized mean difference (SMD) was used in the data description. The heterogeneity was quantitatively determined by I^2^, where I^2^ values of <25, 26–74, and > 75% represented small, moderate, and large levels of heterogeneity, respectively. Fixed-effects models were applied when heterogeneity was graded as small, whereas random-effects models were utilized for moderate or large heterogeneity. For pooled effects with moderate or large heterogeneity, subgroup analyses were conducted based on VR parameters (immersion level, duration, session, and frequency) and demographic factors (geographic region, education level, and male proportion) to identify potential sources and influencing factors. Immersion levels were categorized as non-immersive, semi-immersive, and full immersive. Interactions using a PC monitor, keyboard, and mouse were classified as non-immersive, while more advanced graphics with larger surface displays were categorized as semi-immersive. Full immersive VR, representing the highest level of immersion, was defined as utilizing 3D displays (21). The subgroup classification criteria are presented in Table 1.

**Table 1:** Subgroup classification criteria.

Topics		Categories
VR Parameter	Immersive level	Full, Semi, Non
	Duration (weeks)	≤ 8, 8 < duration ≤16, > 16
	Session (min)	≤ 30, 30 < session ≤60, > 60
	Frequency (times/week)	≤ 2, > 2, NC
Demographic Factors	Geographic Region	Asia, Europe, America, Australia
	Education Level (years)	≤ 9, > 9, NC
	Male Proportion (%)	≤ 40%, > 40%, NC

VR, Virtual Reality; NC, Unrecorded.

If sufficient studies were available (≥ 10 study groups), meta-regression was performed to further analyze the impact of specific covariates. Moreover, publication bias was determined through funnel plot and Egger’s regression test when a sufficiently large sample of studies (≥ 10 study groups) was available for the EG vs. CG comparison (22, 23). In case of publication bias, the “trim and fill” method was used to further evaluate the influence of publication bias on pooled results (24). Sensitivity analyses were performed using the “leave-one-out” method, whereby individual studies were sequentially excluded to examine their influence on the overall estimates (25).

### 2.7 Certainty of the evidence: GRADE approach

We applied the GRADE (Grading of Recommendations, Assessment, Development, and Evaluation) framework to evaluate the certainty of the evidence (26). The assessment was conducted using GRADEPro GDT software (version 2022). This systematic approach evaluates the overall certainty of evidence for each outcome based on five key domains: risk of bias, imprecision, inconsistency, indirectness, and publication bias. Evidence certainty was categorized into four levels: high, moderate, low, or very low, reflecting the confidence that the reported effect is close to the true effect.

## 3 Results

### 3.1 Search results and reported quality

**Figure 1:**
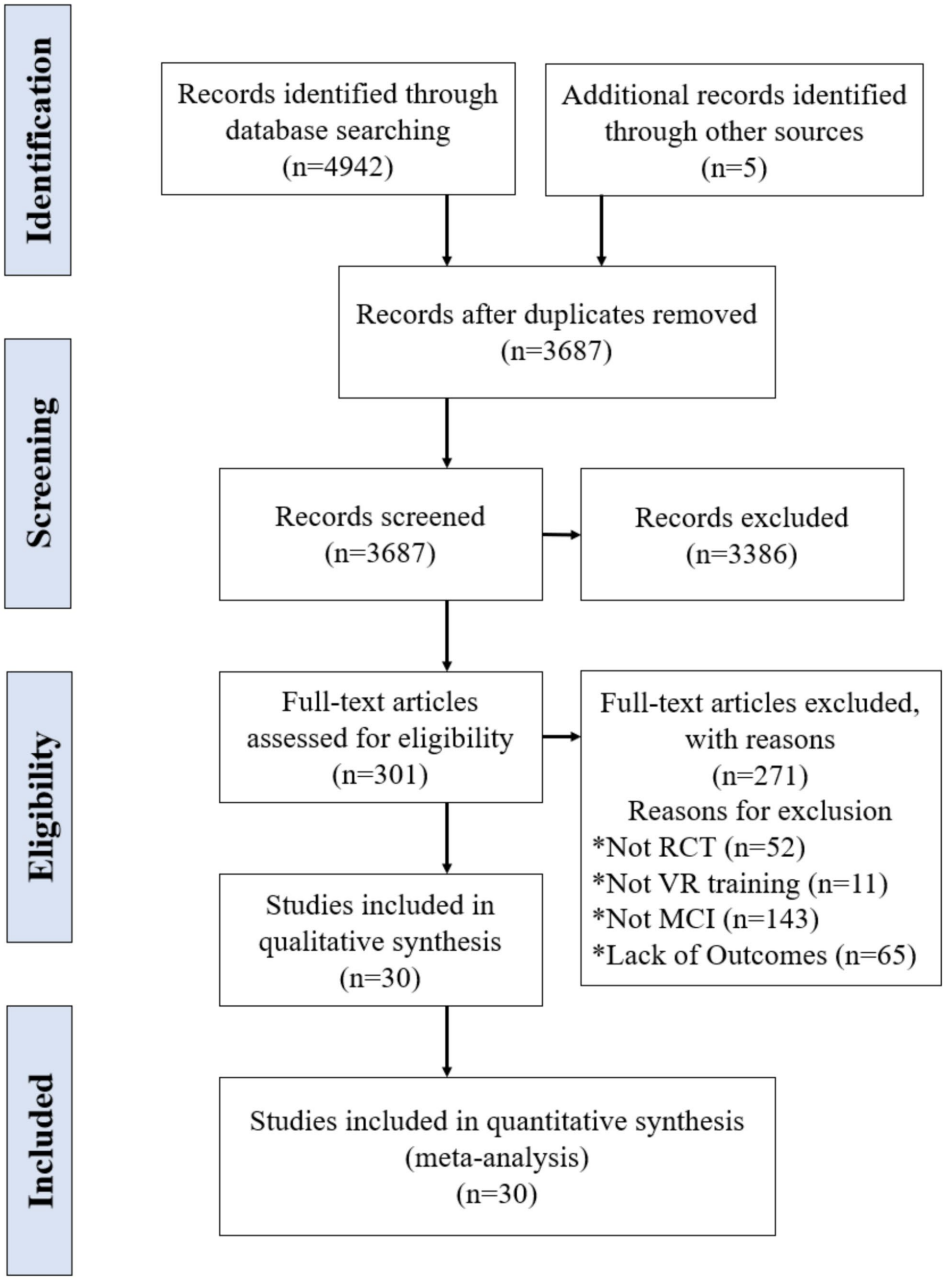
Flow chart of the literature search.

**Figure 2:**
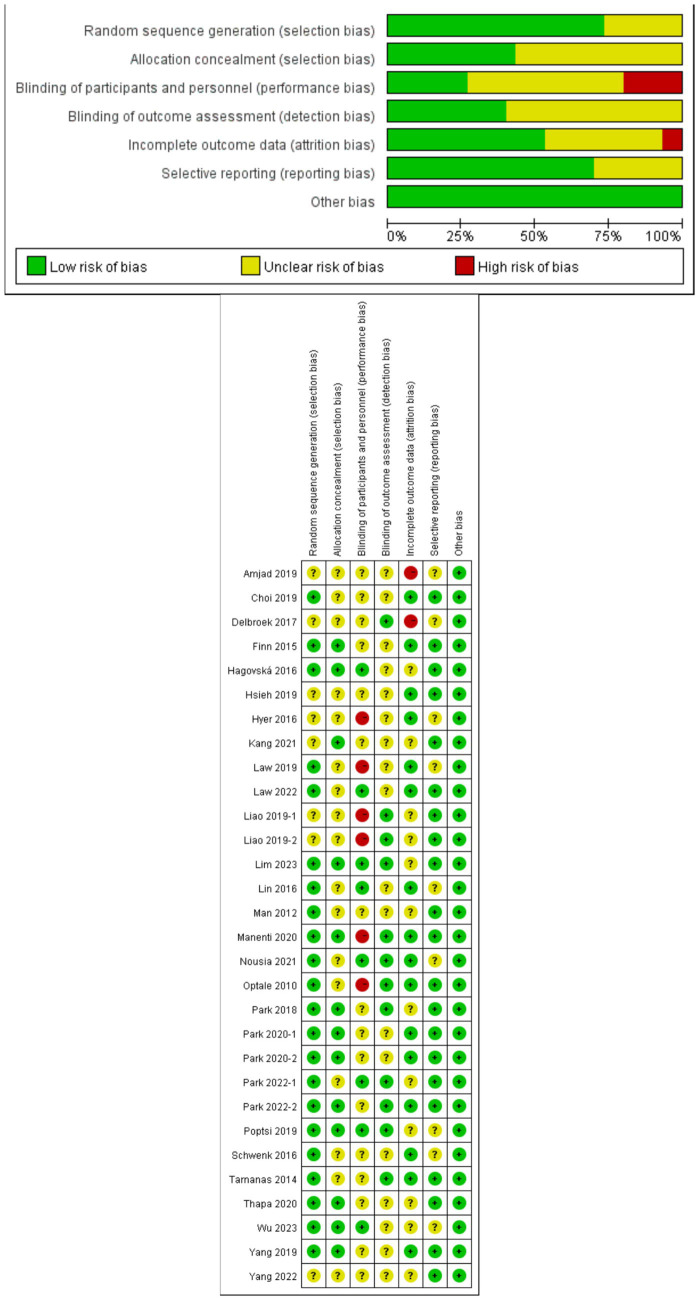
Literature quality and bias risk assessment.

Figure 1 shows the flow diagram of study selection. The initial search generated 4,947 articles of which 1,260 were duplicates. 3,386 articles were excluded by screening titles and abstracts. Out of the remaining 301 articles screened by full text. Finally, 30 original research articles were selected for further analysis. Results of the literature quality and publication bias risk assessment are presented in Figure 2.

### 3.2 Study characteristics

Table 2 summarizes the characteristics of the included studies. All 30 studies were RCTs, and all the included studies were related to elderly people with cognitive disorders or MCI. The 30 studies had a total of 1,365 participants in 9 countries across 4 continents, including 489 male and 876 female. In terms of immersive level, nine studies reported full-immersive VR, seven reported semi-immersive VR, and the remaining fourteen reported non-immersive VR. Training frequency varied from 2 to 5 times per week and the duration per session varied from 18 to 100 min. The duration of interventions ranged from brief 4 weeks programs to 24 weeks of training.

**Table 2:** Characteristics of included studies and interventions of interest.

Author, Year, Country	Sample size (M/F), Age (years), Education (years)	MMSE (score), MoCA (score), IADL (score)	Immersive level	EG intervention	Session (min), Frequency (times/week), Duration (weeks)	CG intervention	Outcome
Liao et al., 2019–1, China (38)	**EG:** 7/11, 75.5 ± 5.2, 9.3 ± 3.8 **CG:** 4/12, 73.1 ± 6.8, 9.9 ± 2.1	**EG:** 27.2 ± 1.9, 22.84 ± 2.69, NC **CG:** 27.2 ± 1.6, 23.15 ± 2.96, NC	Full	Physical tasks: simplified 24-form Yang-style Tai Chi, resistance exercise, aerobic exercise, other functional VR daily activities; Cognitive tasks: IADL based scenarios (take mass rapid transit, looking for a store, kitchen chef, and convenience store clerk)	60 min, 3 times/week, 12 weeks	Combined Physical (Resistance, aerobic and balance exercises) and Cognitive Training (different tasks in ecological scenarios)	TMT-A, TMT-B
Liao et al., 2019–2, China (39)	**EG:** 7/11, 75.5 ± 5.2, 9.3 ± 3.8 **CG:** 4/12, 73.1 ± 6.8, 9.9 ± 2.1	**EG:** 27.2 ± 1.9, 23.0 ± 2.67, 18.16 ± 2.33 **CG:** 27.2 ± 1.6, 23.68 ± 2.65, 18.16 ± 2.33	Full	Physical tasks: simplified 24-form Yang-style Tai Chi, resistance exercise, aerobic exercise, other functional VR daily activities; Cognitive tasks: IADL based scenarios (take mass rapid transit, looking for a store, kitchen chef, and convenience store clerk)	60 min, 3 times/week, 12 weeks	Combined Physical (Resistance, aerobic and balance exercises) and Cognitive Training (different tasks in ecological scenarios)	MoCA, CVVLT-IR, CVVLT-DR, IADL
Park et al., 2018, Korea (40)	**EG:** 20/19, 66.95 ± 4.10, 8.54 ± 4.25 **CG:** 22/17, 67.64 ± 4.55, 8.74 ± 4.51	**EG:** 26.41 ± 1.94, NC, NC **CG:** 26.67 ± 1.68, NC, NC	Non	Nintendo Wii: table tennis, sword play, and archery	30 min, 3 times/week, 10 weeks	CoTras program	TMT-B, WAIS-BDT, RAVLT-IR, DSB, DSF
Park et al., 2020–1, Korea (41)	**EG:** 3/7, 71.80 ± 6.61, 7.20 ± 3.61 **CG:** 4/7 69.45 ± 7.45, 8.00 ± 2.90	**EG:** 25.30 ± 2.41, NC, NC **CG:** 26.18 ± 1.78, NC, NC	Full	Multicomponent restorative cognitive function training: “Crows and Seagulls” game, “Janggu” game, “Automated Teller Machine” game, “Shopping in the Mart” game, “Fireworks Party” game, and “Fruit Cocktail” game	30 min, 2 times/week, 12 weeks	Maintain normal daily activities	GDS-15, MMSE, DSB, DSF, Animal Word, “ㅅ” Word
Park et al., 2020–2, Korea (42)	**EG:** 10/8, 75.8 ± 8.5 NC **CG:** 7/10, 77.2 ± 7.2, NC	**EG:** NC, 17.7 ± 3.4, NC **CG:** NC, 17.8 ± 2.4, NC	Semi	MOTOCOG® system: performing driving, bathing, cooking, and shopping activities	30 min, 5 times/week, 6 weeks	Tabletop activities: including puzzles, wood blocks, card play, stick construction activity, maze and pencil–paper	MoCA, TMT-A, TMT-B, DSB, DSF
Park et al., 2022–1, Korea (43)	**EG:** 9/7, 72.25 ± 5.13, 7.56 ± 3.93 **CG:** 6/10, 70.88 ± 4.51, 7.50 ± 2.89	**EG:** 26.06 ± 1.34, NC, 16.69 ± 3.86 **CG:** 25.50 ± 1.31, NC, 17.81 ± 2.68	Non	Virtual shopping training	30 min, 2 times/week, 8 weeks	NC	IADL
Park et al., 2022–2, Korea (44)	**EG:** 12/16, 71.93 ± 3.11, 8.42 ± 4.23 **CG:** 11/17, 72.04 ± 2.42, 8.78 ± 4.13	**EG:** 26.7 ± 1.2, NC, NC **CG:** 26.4 ± 1.5, NC, NC	Non	Unity game engine	45 min, 3 times/week, 8 weeks	NC	WAIS-BDT
Kang et al., 2021, Korea (45)	**EG:** 6/17, 75.48 ± 4.67, 7.70 ± 4.10 **CG:** 6/12, 73.28 ± 6.96, 8.56 ± 4.83	**EG:** 26.22 ± 2.91, NC, NC **CG:** 26.28 ± 2.87, NC, NC	Full	Multidomain cognitive tasks: find differences, select items needed to perform certain tasks, prepare an exact amount of money, prepare an exact amount of money, spatially place furniture exactly based on a memorized drawing, remember certain words, remember specific flags and symbols, and catch animals in a certain order	20–30 min, 2 times/week, 4 weeks	Usual therapy: pharmacotherapy	MMSE, GDS-30, QoL-AD
Schwenk et al., 2016, America (46)	**EG:** 5/7, 77.8 ± 6.9, 14.2 ± 2.3 **CG:** 5/5, 79.0 ± 10.4, 15.9 ± 2.7	**EG:** NC, 23.3 ± 3.1, NC **CG:** NC, 22.4 ± 3.0, NC	Non	Ankle point-to-point reaching tasks, virtual obstacle crossing tasks	45 min, 2 times/week, 4 weeks	NC	MoCA, TMT-A, TMT-B
Delbroek et al., 2017, Belgium (47)	**EG:** 2/8, 86.9 ± 5.6, NC **CG:** 5/5, 87.5 ± 6.6, NC	**EG:** NC, 17.7 ± 5.3, NC **CG:** NC, 16.8 ± 5.8, NC	Semi	BioRescue training: memory exercise, avoidance whilst walking, hot air balloon, blackboard, spaceshuttle, simple Maze, tortoise, rally, downhill ski	18–30 min, 2 times/week, 6 weeks	Usual care	MoCA, TUG
Tarnanas et al., 2014, Greece (48)	**EG:** 12/20, 70.5 ± 4.3, NC **CG:** 16/23, 69.7 ± 4.5, NC	**EG:** 26.8 ± 3.6, NC, NC **CG:** 26.2 ± 3.6, NC, NC	Semi	Virtual reality museum cognitive training	90 min, 2 times/week, 20 weeks	Learning-based memory training	MMSE, DSB, DSF, GDS-30, TMT-B, RAVLT-IR, RAVLT-DR
Yang et al., 2022, Korea (49)	**EG:** 13/20, 72.5 ± 5.0, 9.5 ± 3.7 **CG:** 3/30, 67.9 ± 3.6, 8.5 ± 3.9	**EG:** 27.21 ± 1.9, NC, NC **CG:** 26.9 ± 1.7, NC, NC	Full	Juice making, crow shooting, find the fireworks number, memory object at the house	100 min, 3 times/week, 8 weeks	Education seminars	MMSE, TMT-A, SDST
Thapa et al., 2020, Korea (50)	**EG:** 6/28, 72.6 ± 5.4, 9.3 ± 4.0 **CG:** 10/24, 72.7 ± 5.6, 8.4 ± 3.5	**EG:** 26.0 ± 1.8, NC, NC **CG:** 26.3 ± 3.3, NC, NC	Full	Juice making, crow shooting, find the fireworks number, memory object at the house	100 min, 3 times/week, 8 weeks	Education seminars	MMSE, TMT-A, TMT-B, SDST
Choi et al., 2019, Korea (51)	**EG:** 5/25, 77.27 ± 4.37, NC **CG:** 4/26, 75.37 ± 3.97, NC	**EG:** NC, 21.10 ± 4.93, NC **CG:** NC, 20.03 ± 3.80, NC	Semi	Virtual kayak paddling exercise	60 min, 2 times/week, 6 weeks	Home exercises	MoCA, TUG, BBS
Hagovská et al., 2016, Slovak Republic (52)	**EG:** 22/18, 68.0 ± 4.4, NC **CG:** 19/21, 65.9 ± 6.2, NC	**EG:** 25.97 ± 2.57, NC, NC **CG:** 26.02 ± 1.47, NC, NC	Semi	CogniPlus training program: “Alert,” “Pland,” “Names,” “N-back,” “Vismo”; Balance training: Walk over obstacles, walk with direction change and walk with speed change, walk with load, and walk up and down the stairs	30 min, 2 times/week, 10 weeks	Balance training: Walk over obstacles, walk with direction change and walk with speed change, walk with load, and walk up and down the stairs	MMSE, TUG
Hsieh et al., 2019, China (53)	**EG:** 7/24, 76.4 ± 7.6, NC **CG:** 10/19, 80.0 ± 7.5, NC	**EG:** NC, NC, NC **CG:** NC, NC, NC	Full	Xbox 360 Kinect: “Your Shape Fitness Evolved 2012”	60 min, 2 times/week, 24 weeks	Maintain usual daily physical activities	TUG, GDS-15
Amjad et al., 2019, Pakistan (54)	**EG:** 22, 62.8 ± 5.1, NC **CG:** 22, 65.6 ± 5.0, NC	**EG:** 22.47 ± 0.36, 22 ± 0.36, NC **CG:** 22.74 ± 0.54, 21.27 ± 0.6, NC	Full	Xbox 360 Kinect: “Body and Brain Exercises”	25–30 min, 5 times/week, 6 weeks	Range of motion exercises	MMSE, MoCA
Law et al., 2019, China (55)	**EG:** 7/8, 76.93 ± 6.79, NC **CG:** 5/9, 75.14 ± 8.53, NC	**EG:** NC, NC, 17.93 ± 5.71 **CG:** NC, NC, 18.64 ± 4.94	Non	Center-based computer cognitive training program	60 min, NC, 8 weeks	Usual medical care	TMT-A, TMT-B, IADL, CVVLT-IR, CVVLT-DR
Law et al., 2022, China (56)	**EG:** 13/25, 76.32 ± 7.21, NC **CG:** 12/24, 74.14 ± 7.53, NC	**EG:** NC, NC, 18.76 ± 5.11 **CG:** NC, NC, 18.44 ± 4.16	Non	Center-based computer cognitive training program	60 min, NC, 8 weeks	Maintained normal activities and exercise practice	Animal word, BBS, IADL
Lin et al., 2016, America (57)	**EG:** 5/5, 72.9 ± 8.2, NC **CG:** 6/5, 73.1 ± 9.6, NC	**EG:** NC, 24.4 ± 2.6, 19.8 ± 6.6 **CG:** NC, 25.6 ± 1.6, 14.2 ± 4.6	Semi	INSIGHT online program: eye for detail, peripheral challenge, visual sweeps, double decision, and target tracker	60 min, 4 times/week, 6 weeks	Mental leisure activities	IADL
Optale et al., 2010, Italy (58)	**EG:** 5/10, 78.5 ± 10.9, 5.3 ± 2.4 **CG:** 5/11, 81.6 ± 5.0, 6.0 ± 3.5	**EG:** 22.9 ± 5.0, NC, NC **CG:** 20.99 ± 4.75, NC, NC	Full	Auditory and VR experience	30 min, 2–3 times/week, 24 weeks	Music therapy	GDS-15, CDT, MMSE
Yang et al., 2019, China (59)	**EG:** 8/25, 75.4 ± 6.6, NC **CG:** 6/27, 81.7 ± 7.2, NC	**EG:** 27.4 ± 2.1, 24.6 ± 4.1, 7.6 ± 1.2 **CG:** 26.6 ± 2.4, 23.2 ± 4.0, 8.0 ± 0.2	Semi	CogniPlus interaction system: updating—visual memory task, spatial encoding memory task, rehearsal—visuospatial training task, and updating—spatial memory task	45 min, 3 times/week, 12 weeks	Reading online e-books and playing online games	MMSE, MoCA, DSB
Lim et al., 2023, Korea (60)	**EG:** 3/9, 75.42 ± 5.74, NC **CG:** 4/8, 73.33 ± 17.52, NC	**EG:** 24.42 ± 1.98, 20.33 ± 4.70, NC **CG:** 23.83 ± 2.89, 19.25 ± 4.71, NC	Non	Brain Talk™ program: pizza, fish, catch a ball, and Xylophone	30 min, 3 times/week, 4 weeks	Performed daily activities	MMSE, MoCA, TMT-B, Animal word, “ㅅ” word
Man et al., 2012, China (61)	**EG:** 3/17, 80.3 ± 1.21 NC **CG:** 2/22, 80.28 ± 1.31, NC	**EG:** 21.05 ± 3.79, NC, 23.70 ± 3.48 **CG:** 23.00 ± 3.96, NC, 21.85 ± 4.72	Non	Home setting and convenience shop management	30 min, 2–3 times/week, 4–5 weeks	Therapist-led training	IADL
Wu et al., 2023, China (62)	**EG:** 7/20, 68.0 ± 8.16, 9 ± 2.23 **CG:** 5/21, 65.5 ± 5.19, 9 ± 2.97	**EG:** NC, NC, NC **CG:** NC, NC, NC	Non	Computerized cognitive training: Warm Up, Visuospatial Function, Auditory Discrimination, Visual Search, Attention – Sustained, Attention – Selection, Attention – Divided, Working Memory Planning, Response Inhibition (Go/No Go), and Mental Rotation	60 min, 3 times/week, 8 weeks	Health education program	CVVLT-IR, CVVLT-DR
Nousia et al., 2021, Greece (63)	**EG:** 6/19, 71.20 ± 5.07, 8.92 ± 3.37 **CG:** 5/16, 71.90 ± 6.24, 8.43 ± 3.06	**EG:** NC, 21.80 ± 1.38, 8.20 ± 0.50 **CG:** NC, 21.86 ± 1.85, 8.29 ± 0.46	Non	RehaCom software	60 min, 2 times/week, 15 weeks	Standard clinical care	TMT-A, TMT-B, DSB, DSF
Manenti et al., 2020, Italy (64)	**EG:** 13/5, 75.3 ± 3.3, 11.8 ± 4.8 **CG:** 7/10, 78.1 ± 4.1, 9.8 ± 3.7	**EG:** NC, NC, NC **CG:** NC, NC, NC	Non	Clinic-VR and Tele@H-VR rehabilitation system	60 min, 3 times/week, 16 weeks	Face-to-face cognitive conventional rehabilitation	CDT, TMT-A, TMT-B, QoL-AD, RAVLT-IR, RAVLT-DR
Poptsi et al., 2019, Greece (65)	**EG:** 5/9, 67.86 ± 9.85, 12.14 ± 3.25 **CG:** 4/10, 68.14 ± 6.90, 10.36 ± 4.81	**EG:** 28.07 ± 1.63, NC, NC **CG:** 26.07 ± 3.05, NC, NC	Non	Computer-based program of language tasks	60 min, 2 times/week, 24 weeks	Without any kind of pharmaceutical or cognitive intervention	MMSE, RAVLT-IR, RAVLT-DR
Hyer et al., 2016, America (66)	**EG:** 17/17, 75.1 ± 7.4, NC **CG:** 15/19, 75.2 ± 7.8, NC	**EG:** NC, NC, NC **CG:** NC, NC, NC	Non	Cogmed computer training program	40 min, 4–5 times/week, 5–7 weeks	Sham training	TMT-B, CFQ
Finn et al., 2015, Australia (67)	**EG:** 8/4, 72.83 ± 5.7, 13.75 ± 2.8 **CG:** 9/3, 75.08 ± 7.5, 13.67 ± 3.8	**EG:** 27.75 ± 1.3, NC, NC **CG:** 27.83 ± 1.9, NC, NC	Non	Repetition-lag training	40 min, 2 times/week, 4 weeks	NC	CFQ

BBS, Berg Balance Scale; CDT, Clock Drawing Test; CFQ, Cognitive Failure Questionnaire; CG, Control Group; CVVLT-DR, Chinese Version Verbal Learning Test-Delayed Recall; CVVLT-IR, Chinese Version Verbal Learning Test-Immediate Recall; DSB, Digit Span Backward; DSF, Digit Span Forward; EG, Experiment Group; GDS-15, Geriatric Depression Scale-15; GDS-30, Geriatric Depression Scale-30; IADL, Instrumental Activity of Daily Living; M/F, Male/Female; MMSE, Mini-mental State Examination; MoCA, Montreal Cognitive Assessment; NC, Unrecorded; QoL-AD, Quality of Life-Alzheimer Disease; RAVLT-DR, Rey Auditory Verbal Learning Test-Delayed Recall; RAVLT-IR, Rey Auditory Verbal Learning Test-Immediate Recall; SDST, Symbol Digit Substitution Test; TMT-A, Trail Making Test–Part A; TMT-B, Trail Making Test–Part B; TUG, Timed Up-and-Go Test; VR, Virtual reality; WAIS-BDT, Wechsler Adult Intelligence Scale-Block Design Test.

### 3.3 Primary outcomes and subgroup analysis

#### 3.3.1 Global cognition

MoCA, MMSE, SDST, and CFQ were used to evaluate global cognition in the studies. Eleven, fourteen, two, and three studies reported MoCA, MMSE, SDST, and CFQ results for 466, 661, 134, and 160 participants, respectively. The meta-analysis revealed that the MoCA (SMD = 0.82, 95% CI: 0.27 to 1.38, I^2^ = 86%, *p* = 0.003, GRADE: moderate) and MMSE (SMD = 0.83, 95% CI: 0.40 to 1.26, I^2^ = 84%, *p* = 0.0001, GRADE: low) in the EG than in the CG. There was no difference in SDST (SMD = 1.14, 95% CI: −0.50 to 2.78, I^2^ = 95%, *p* = 0.17, GRADE: very low) and CFQ (SMD = −0.09, 95% CI: −0.40 to 0.22, I^2^ = 7%, *p* = 0.59, GRADE: low) between groups (Table 3; Supplementary Figure 1).

**Table 3:** Meta-analysis results.

Outcome	Study quantity	Sample size (*n*)		SMD (95% CI)	Heterogeneity effect		Overall effect		GRADE
		EG	CG		I^2^	*P*	Z	*P*	
Global cognition									
MoCA	11	232	234	0.82 (0.27, 1.38)	86%	< 0.0001	2.92	0.003	⨁⨁⨁◯ Moderate
MMSE	14	328	333	0.83 (0.40, 1.26)	84%	< 0.0001	3.82	0.0001	⨁⨁◯◯ Low
SDST	2	67	67	1.14 (−0.50, 2.78)	95%	< 0.0001	1.36	0.17	⨁◯◯◯ Very Low
CFQ	3	80	80	−0.09 (−0.40, 0.22)	7%	0.34	0.54	0.59	⨁⨁◯◯ Low
Execution function									
TMT-A	10	208	197	−0.26 (−0.55, −0.03)	52%	0.03	1.73	0.08	⨁⨁⨁◯ Moderate
TMT-B	15	338	334	−0.29 (−0.64, 0.07)	80%	< 0.0001	1.58	0.11	⨁⨁⨁◯ Moderate
Attention									
DSB	7	189	194	0.61 (0.21, 1.02)	72%	0.001	2.95	0.003	⨁⨁◯◯ Low
DSF	5	123	128	0.89 (0.34, 1.45)	75%	0.003	3.15	0.002	⨁⨁◯◯ Low
Memory									
RAVLT-IR	6	139	143	−0.01 (−0.38, 0.36)	57%	0.04	0.06	0.95	⨁⨁◯◯ Low
RAVLT-DR	5	100	104	0.13 (−0.35, 0.62)	66%	0.02	0.53	0.59	⨁⨁◯◯ Low
CVVLT-IR	3	60	56	0.13 (−0.24, 0.50)	0%	0.39	0.69	0.49	⨁⨁◯◯ Low
CVVLT-DR	3	60	56	0.41 (−0.58, 1.39)	85%	0.002	0.81	0.42	⨁◯◯◯ Very Low
Verbal fluency									
Animal word	5	110	107	0.20 (−0.06, 0.47)	0%	0.50	1.49	0.14	⨁⨁⨁◯ Moderate
“ㅅ” word	3	34	35	−0.00 (−0.48, 0.47)	0%	0.42	0.00	1.00	⨁⨁◯◯ Low
Visual ability									
WAIS-BDT	2	67	67	0.44 (−0.37, 1.24)	81%	0.02	1.06	0.29	⨁◯◯◯ Very Low
CDT	5	84	83	0.21 (−0.30, 0.72)	63%	0.03	0.82	0.41	⨁◯◯◯ Very Low
Emotional status									
GDS-15	5	102	101	−0.40 (−1.17, 0.37)	85%	< 0.0001	1.02	0.31	⨁⨁◯◯ Low
GDS-30	2	55	57	−1.38 (−4.51, 1.76)	98%	< 0.0001	0.86	0.39	⨁◯◯◯ Very Low
Quality of life									
IADL	7	155	151	0.22 (0.00, 0.45)	0%	0.68	1.94	0.049	⨁⨁⨁◯ Moderate
QoL-AD	4	77	69	−0.06 (−0.39, 0.26)	0%	0.96	0.38	0.71	⨁⨁◯◯ Low
Dynamic balance									
TUG	5	142	138	0.05 (−0.45, 0.56)	76%	0.002	0.21	0.83	⨁⨁◯◯ Low
BBS	3	106	102	0.45 (−0.51, 1.41)	91%	< 0.0001	0.93	0.35	⨁⨁◯◯ Low

BBS, Berg Balance Scale; CDT, Clock Drawing Test; CFQ, Cognitive Failure Questionnaire; CG, Control Group; CI, Confidence Interval; CVVLT-DR, Chinese Version Verbal Learning Test-Delayed Recall; CVVLT-IR, Chinese Version Verbal Learning Test-Immediate Recall; DSB, Digit Span Backward; DSF, Digit Span Forward; EG, Experiment Group; GDS-15, Geriatric Depression Scale-15; GDS-30, Geriatric Depression Scale-30; GRADE, Grading of Recommendations, Assessment, Development, and Evaluation; IADL, Instrumental Activity of Daily Living; MMSE, Mini-Mental State Examination; MoCA, Montreal Cognitive Assessment; QoL-AD, Quality of Life-Alzheimer Disease; RAVLT-DR, Rey Auditory Verbal Learning Test-Delayed Recall; RAVLT-IR, Rey Auditory Verbal Learning Test-Immediate Recall; SDST, Symbol Digit Substitution Test; SMD, Standardized Mean Difference; TMT-A, Trail Making Test–Part A; TMT-B, Trail Making Test–Part B; TUG, Timed Up-and-Go Test; WAIS-BDT, Wechsler Adult Intelligence Scale-Block Design Test.

The subgroup analysis of MoCA scores showed that the following factors were significantly associated with better outcomes: semi-immersive level (*p* < 0.0001), duration ≤16 weeks (*p* < 0.05), session ≤60 min (*p* < 0.05), frequency > 2 times/week (*p* = 0.005), participants from Asia and Europe (*p* < 0.05), and male proportion ≤ 40% (*p* < 0.0001) (Table 4; Supplementary Figure 2). Meta-regression analysis further validated these results, indicating that immersive level (*p* = 0.08), duration (*p* = 0.011), session (*p* = 0.012), frequency (*p* = 0.011), geographic region (*p* = 0.010), and education level (*p* = 0.007) were significant factors affecting the improvement in MoCA scores (Table 5).

**Table 4:** Subgroup analysis results.

Subgroup		Study quantity	Sample size (*n*)		SMD (95% CI)	Overall effect		Subgroup difference
Topics	Categories		EG	CG		Z	*P*	
MoCA								
Immersive level	Full	2	40	38	4.29 (−3.98, 12.57)	1.02	0.31	0.42
	Semi	6	156	162	0.58 (0.36, 0.81)	5.08	< 0.0001	
	Non	3	36	34	0.22 (−0.46, 0.91)	0.65	0.52	
Duration (weeks)	≤ 8	7	116	113	1.32 (0.26, 2.38)	2.44	0.01	0.28
	8 < duration ≤16	3	84	82	0.42 (0.11, 0.73)	2.66	0.008	
	> 16	1	32	39	0.46 (−0.01, 0.94)	1.92	0.05	
Session (min)	≤ 30	5	74	73	1.95 (0.36, 3.54)	2.40	0.02	0.17
	30 < session ≤60	5	126	122	0.37 (0.03, 0.72)	2.12	0.03	
	> 60	1	32	39	0.46 (−0.01, 0.94)	1.92	0.05	
Frequency (times/week)	≤ 2	4	84	89	0.37 (−0.09, 0.82)	1.57	0.12	0.07
	> 2	7	148	145	1.28 (0.39, 2.17)	2.82	0.005	
Geographic region	Asia	6	154	151	1.42 (0.47, 2.38)	2.92	0.003	0.01
	Europe	4	66	73	0.49 (0.15, 0.83)	2.83	0.005	
	America	1	12	10	−0.49 (−1.34, 0.37)	1.12	0.26	
Education level (years)	> 9	2	30	26	−0.12 (−0.71, 0.46)	0.41	0.68	0.007
	NC	9	202	208	1.06 (0.43, 1.69)	3.30	0.001	
Male proportion (%)	≤ 40%	8	180	185	0.50 (0.29, 0.71)	4.68	< 0.0001	< 0.0001
	> 40%	2	30	27	0.29 (−1.19, 1.77)	0.38	0.70	
	NC	1	22	22	8.57 (6.60, 10.53)	8.54	< 0.0001	
MMSE								
Immersive level	Full	7	152	150	1.08 (0.13, 2.03)	2.23	0.03	0.40
	Semi	4	138	145	0.57 (0.33, 0.81)	4.68	< 0.0001	
	Non	3	38	38	0.84 (0.36, 1.31)	3.47	0.005	
Duration (weeks)	≤ 8	6	136	131	0.92 (0.02, 1.82)	2.01	0.04	0.008
	8 < duration ≤16	4	116	117	0.37 (0.08, 0.66)	2.47	0.01	
	> 16	4	76	85	1.39 (0.79, 1.98)	4.58	< 0.0001	
Session (min)	≤ 30	8	149	147	1.16 (0.29, 2.02)	2.61	0.009	0.36
	30 < session ≤60	3	80	80	0.56 (0.24, 0.87)	3.44	0.0006	
	> 60	3	99	106	0.44 (−0.04, 0.92)	1.79	0.07	
Frequency (times/week)	≤ 2	5	119	122	0.35 (−0.15, 0.86)	1.37	0.17	0.05
	> 2	9	209	211	1.15 (0.52, 1.78)	3.60	0.0003	
Geographic region	Asia	7	188	184	0.61 (−0.07, 1.30)	1.76	0.08	0.29
	Europe	7	140	149	1.05 (0.61, 1.49)	4.71	< 0.0001	
Education level (years)	≤ 9	4	63	61	0.81 (−0.49, 2.11)	1.23	0.22	0.13
	> 9	3	81	81	0.33 (−0.04, 0.70)	1.75	0.08	
	NC	7	184	191	1.05 (0.43, 1.67)	3.33	0.0009	
Male proportion (%)	≤ 40%	12	266	271	2.13 (0.93, 3.33)	3.48	0.0005	0.004
	> 40%	1	40	40	0.87 (−0.05, 1.79)	1.86	0.06	
	NC	1	22	22	2.53 (2.19, 2.87)	14.65	< 0.0001	
TMT-A								
Immersive level	Full	3	85	83	−0.32 (−0.62, −0.01)	2.02	0.04	0.10
	Semi	1	17	18	−1.01 (−1.72, −0.30)	2.80	0.005	
	Non	6	106	96	−0.11 (−0.54, 0.32)	0.51	0.61	
Duration (weeks)	≤ 8	5	111	109	−0.37 (−0.72, −0.02)	2.07	0.04	0.46
	8 < duration ≤16	5	97	88	−0.14 (−0.63, 0.35)	0.57	0.57	
Session (min)	≤ 30	1	17	18	−1.01 (−1.72, −0.30)	2.80	0.005	0.07
	30 < session ≤60	7	124	112	−0.09 (−0.45, 0.28)	0.47	0.64	
	> 60	2	67	67	−0.42 (−0.76, −0.07)	2.38	0.02	
Frequency (times/week)	≤ 2	2	37	31	−0.54 (−1.63, 0.54)	0.98	0.33	0.57
	> 2	7	156	152	−0.22 (−0.53, 0.09)	1.41	0.16	
	NC	1	15	14	0.12 (−0.61, 0.85)	0.32	0.75	
Geographic region	Asia	5	117	115	−0.34 (−0.70, 0.01)	1.90	0.06	0.68
	Europe	4	79	72	−0.19 (−0.81, 0.42)	0.62	0.54	
	America	1	12	10	0.05 (−0.79, 0.89)	0.12	0.91	
Education level (years)	≤ 9	1	25	21	−1.06 (−1.68, −0.44)	3.33	0.0009	0.02
	> 9	7	151	144	−0.14 (−0.37, 0.09)	1.17	0.24	
	NC	2	32	32	−0.45 (−1.56, 0.66)	0.79	0.43	
Male proportion (%)	≤ 40%	4	110	104	−0.46 (−0.87, −0.05)	2.21	0.03	0.19
	> 40%	6	98	93	−0.09 (−0.47, 0.30)	0.44	0.66	
TMT-B								
Immersive level	Full	2	52	50	−0.32 (−0.72, 0.07)	1.60	0.11	0.09
	Semi	2	49	57	−1.46 (−2.73, −0.19)	2.25	0.02	
	Non	11	237	227	−0.08 (−0.38, 0.22)	0.51	0.61	
Duration (weeks)	≤ 8	8	170	168	−0.10 (−0.39, 0.20)	0.65	0.51	< 0.0001
	8 < duration ≤16	6	136	127	−0.27 (−0.76, 0.22)	1.06	0.29	
	> 16	1	32	39	−2.09 (−2.68, −1.51)	7.00	< 0.0001	
Session (min)	≤ 30	4	80	81	−0.08 (−0.54, 0.38)	0.33	0.74	0.37
	30 < session ≤60	9	192	180	−0.15 (−0.51, 0.21)	0.81	0.42	
	> 60	2	66	73	−1.27 (−2.86, 0.32)	1.57	0.12	
Frequency (times/week)	≤ 2	3	69	70	−1.02 (−2.52, 0.47)	1.34	0.18	0.40
	> 2	11	254	250	−0.12 (−0.29, 0.06)	1.29	0.20	
	NC	1	15	14	0.12 (−0.61, 0.85)	0.32	0.75	
Geographic region	Asia	5	123	121	−0.20 (−0.55, 0.16)	1.10	0.27	0.37
	Europe	7	135	135	−0.53 (−1.25, 0.20)	1.42	0.15	
	America	3	80	78	0.08 (−0.40, 0.56)	0.33	0.74	
Education level (years)	≤ 9	2	64	60	−0.69 (−2.35, 0.98)	0.81	0.42	0.61
	> 9	6	118	111	−0.11 (−0.38, 0.16)	0.78	0.43	
	NC	7	156	163	−0.39 (−1.03, 0.26)	1.18	0.24	
Male proportion (%)	≤ 40%	6	133	134	−0.66 (−1.42, 0.10)	1.70	0.09	0.12
	> 40%	9	205	200	−0.04 (−0.26, 0.18)	0.35	0.72	
DSB								
Immersive level	Full	1	10	11	−0.07 (−0.93, 0.78)	0.16	0.87	0.24
	Semi	4	115	123	0.49 (0.23, 0.75)	3.71	0.0002	
	Non	2	64	60	1.11 (−0.01, 2.24)	1.94	0.05	
Duration (weeks)	≤ 8	1	17	18	0.30 (−0.37, 0.97)	0.88	0.38	0.45
	8 < duration ≤16	5	140	137	0.73 (0.20, 1.26)	2.69	0.007	
	> 16	1	32	39	0.31 (−0.16, 0.78)	1.30	0.19	
Session (min)	≤ 30	3	66	68	0.67 (−0.45, 1.79)	1.18	0.24	0.56
	30 < session ≤60	3	91	87	0.61 (0.31, 0.92)	3.99	< 0.0001	
	> 60	1	32	39	0.31 (−0.16, 0.78)	1.30	0.19	
Frequency (times/week)	≤ 2	3	67	71	0.32 (−0.01, 0.66)	1.88	0.06	0.14
	> 2	4	122	123	0.83 (0.24, 1.42)	2.75	0.006	
Geographic region	Asia	5	132	134	0.68 (0.12, 1.25)	2.38	0.02	0.40
	Europe	2	57	60	0.40 (0.03, 0.77)	2.12	0.03	
Education level (years)	≤ 9	3	74	71	0.75 (−0.26, 1.76)	1.46	0.14	0.62
	NC	4	115	123	0.49 (0.23, 0.75)	3.71	0.0002	
Male proportion (%)	≤ 40%	5	133	137	0.48 (0.23, 0.72)	3.85	0.0001	0.45
	> 40%	2	56	57	1.01 (−0.34, 2.36)	1.46	0.14	
DSF								
Immersive level	Full	1	10	11	0.00 (−0.86, 0.86)	0.00	1.00	0.10
	Semi	2	49	57	1.03 (0.62, 1.44)	4.95	< 0.0001	
	Non	2	64	60	1.07 (−0.19, 2.34)	1.66	0.10	
Duration (weeks)	≤ 8	1	17	18	1.15 (0.42, 1.87)	3.11	0.002	0.82
	8 < duration ≤16	3	74	71	0.74 (−0.30, 1.79)	1.40	0.16	
	> 16	1	32	39	0.98 (0.48, 1.48)	3.87	0.0001	
Session (min)	≤ 30	3	66	68	1.00 (0.05, 1.95)	2.07	0.04	0.32
	30 < session ≤60	1	25	21	0.42 (−0.17, 1.01)	1.40	0.16	
	> 60	1	32	39	0.98 (0.48, 1.48)	3.87	0.0001	
Frequency (times/week)/Male proportion (%)	≤ 2/≤ 40%	3	67	71	0.54 (0.00, 1.09)	1.96	0.05	0.02
	> 2/> 40%	2	56	57	1.49 (0.94, 2.03)	5.35	< 0.0001	
Geographic region	Asia	3	66	68	1.00 (0.05, 1.95)	2.07	0.04	0.62
	Europe	2	57	60	0.72 (0.17, 1.27)	2.59	0.010	
Education level (years)	≤ 9	3	74	71	0.74 (−0.30, 1.79)	1.40	0.16	0.62
	NC	2	49	57	1.03 (0.62, 1.44)	4.95	< 0.0001	
RAVLT-IR								
Immersive level	Semi	1	32	39	0.68 (0.20, 1.16)	2.76	0.006	0.003
	Non	5	107	104	−0.17 (−0.44, 0.10)	1.20	0.23	
Duration (weeks)/Frequency (times/week)/Male proportion (%)	8 < duration ≤16/≤ 2/> 40%	4	93	90	−0.20 (−0.49, 0.09)	1.33	0.18	0.08
	> 16/> 2/≤ 40%	2	46	53	0.42 (−0.20, 1.04)	1.32	0.19	
Session (min)/Education level (years)	≤ 30/≤ 9	1	39	39	−0.07 (−0.51, 0.38)	0.30	0.76	0.010
	30 < session ≤60/> 9	4	68	65	−0.23 (−0.57, 0.12)	1.29	0.20	
	> 60/NC	1	32	39	0.68 (0.20, 1.16)	2.76	0.006	
Geographic region	Asia	1	39	39	−0.07 (−0.51, 0.38)	0.30	0.76	0.86
	Europe	5	100	104	−0.01 (−0.49, 0.47)	0.03	0.97	
RAVLT-DR								
Immersive level/Session (min)/Education level (years)	Semi/> 60/NC	4	68	65	−0.11 (−0.45, 0.23)	0.61	0.54	0.0009
	Non/30 < session ≤60/> 9	1	32	39	0.91 (0.42, 1.40)	3.62	0.0003	
Duration (weeks)/Frequency (times/week)/Male proportion (%)	8 < duration ≤16/> 2/> 40%	3	54	51	−0.13 (−0.51, 0.26)	0.65	0.52	0.23
	> 16/≤ 2/≤ 40%	2	46	53	0.48 (−0.43, 1.40)	1.03	0.30	
CVVLT-DR								
Immersive level/Duration (weeks)	Full/8 < duration ≤16	1	18	16	0.19 (−0.48, 0.87)	0.55	0.58	0.73
	Non/≤ 8	2	42	40	0.50 (−1.12, 2.12)	0.61	0.55	
Frequency (times/week)/Male proportion (%)	> 2/> 40%	2	45	42	0.76 (−0.34, 1.87)	1.36	0.17	0.10
	NC/≤ 40%	1	15	14	−0.34 (−1.08, 0.39)	0.91	0.36	
Education level (years)	≤ 9	1	27	26	1.32 (0.72, 1.91)	4.31	< 0.0001	0.002
	> 9	1	18	16	0.19 (−0.48, 0.87)	0.55	0.58	
	NC	1	15	14	−0.34 (−1.08, 0.39)	0.91	0.36	
CDT								
Immersive level/Duration (weeks)/Session (min)/Male proportion (%)	Full/> 16/≤ 30/≤ 40%	2	30	32	0.86 (0.33, 1.38)	3.19	0.001	0.002
	Non/8 < duration ≤16/30 < session ≤60/> 40%	3	54	51	−0.18 (−0.57, 0.20)	0.94	0.35	
GDS-15								
Duration (weeks)	8 < duration ≤16	1	10	11	0.16 (−0.70, 1.02)	0.37	0.71	0.28
	> 16	4	92	90	−0.54 (−1.46, 0.39)	1.13	0.26	
Session (min)/Education level (years)	≤ 30/≤ 9	3	40	43	−0.91 (−1.93, 0.11)	1.75	0.08	0.04
	30 < session ≤60/NC	2	62	58	0.26 (−0.14, 0.67)	1.30	0.20	
Frequency (times/week)/Geographic region	≤ 2/Asia	3	72	69	0.25 (−0.08, 0.58)	1.47	0.14	< 0.0001
	> 2/Europe	2	30	32	−1.43 (−2.00, −0.87)	4.95	< 0.0001	
TUG								
Immersive level	Full	2	62	58	0.59 (0.22, 0.96)	3.16	0.002	0.005
	Semi	3	80	80	−0.32 (−0.84, 0.20)	1.21	0.22	
Duration (weeks)	≤ 8	2	40	40	−0.62 (−1.07, −0.17)	2.69	0.007	0.0002
	8 < duration ≤16	1	40	40	0.06 (−0.38, 0.50)	0.27	0.79	
	> 16	2	62	58	0.59 (0.22, 0.96)	3.16	0.002	
Session (min)/Geographic region	≤ 30/Europe	2	50	50	−0.04 (−0.43, 0.36)	0.18	0.86	0.67
	30 < session ≤60/Asia	3	92	88	0.17 (−0.66, 1.00)	0.39	0.69	
Male proportion (%)	≤ 40%	4	102	98	0.04 (−0.65, 0.74)	0.12	0.90	0.97
	> 40%	1	40	40	0.06 (−0.38, 0.50)	0.27	0.79	
BBS								
Immersive level/Frequency (times/week)	Semi/≤ 2	1	30	30	1.55 (0.96, 2.13)	5.21	< 0.0001	< 0.0001
	Non/> 2	2	76	72	−0.07 (−0.39, 0.26)	0.40	0.69	

BBS, Berg Balance Scale; CDT, Clock Drawing Test; CG, Control Group; CI, Confidence Interval; CVVLT-DR, Chinese Version Verbal Learning Test-Delayed Recall; DSB, Digit Span Backward; DSF, Digit Span Forward; EG, Experiment Group; GDS-15, Geriatric Depression Scale-15; MMSE, Mini-mental State Examination; MoCA, Montreal Cognitive Assessment; NC, Unrecorded; RAVLT-DR, Rey Auditory Verbal Learning Test-Delayed Recall; RAVLT-IR, Rey Auditory Verbal Learning Test-Immediate Recall; SMD, Standardized Mean Difference; TMT-A, Trail Making Test–Part A; TMT-B, Trail Making Test–Part B; TUG, Timed Up-and-Go Test; VR, Virtual reality.

**Table 5:** Meta-regression results.

Covariates	Coefficient	Standard error	95% CI	*p*
MoCA				
Immersive level	−7.609	1.201	(−11.43, −3.78)	0.008
Duration (weeks)	−7.460	1.319	(−11.66, −3.26)	0.011
Session (min)	7.318	1.356	(2.99, 11.63)	0.012
Frequency (times/week)	7.366	1.303	(3.21, 11.51)	0.011
Geographic region	7.289	11.266	(3.25, 11.32)	0.010
Education level (years)	8.198	1.216	(4.32, 12.06)	0.007
Male proportion (%)	0.180	0.350	(−0.93, 1.29)	0.643
MMSE				
Immersive level	0.412	0.572	(−0.98, 1.81)	0.498
Duration (weeks)	0.510	0.299	(−0.22, 1.24)	0.140
Session (min)	0.203	0.331	(−0.60, 1.01)	0.562
Frequency (times/week)	1.435	0.448	(0.33, 2.53)	0.019
Geographic region	0.328	0.590	(−1.11, 1.77)	0.598
Education level (years)	−0.603	0.523	(−1.88, 0.67)	0.293
Male proportion (%)	2.074	0.649	(0.48, 3.66)	0.019
TMT-A				
Immersive level	0.717	0.308	(−0.80, 1.15)	0.616
Duration (weeks)	1.480	0.469	(−0.01, 2.97)	0.051
Session (min)	0.986	0.303	(0.01, 1.95)	0.048
Frequency (times/week)	−10.559	3.047	(−20.25, −0.86)	0.040
Geographic region	1.423	0.645	(−0.63, 3.47)	0.115
Education level (years)	2.918	1.199	(−0.90, 6.73)	0.093
Male proportion (%)	−1.732	1.243	(−5.69, 2.22)	0.258
TMT-B				
Immersive level	−0.355	0.380	(−1.25, 0.54)	0.382
Duration (weeks)	−0.238	0.322	(−1.00, 0.52)	0.484
Session (min)	−0.440	0.333	(−1.22, 0.34)	0.228
Frequency (times/week)	1.081	0.533	(−0.17, 2.34)	0.082
Geographic region	0.693	0.421	(−0.30, 1.68)	0.144
Education level (years)	−0.501	0.267	(−1.13, 0.13)	0.104
Male proportion (%)	0.111	0.401	(−0.83, 1.06)	0.790

CI, Confidence Interval; MMSE, Mini-Mental State Examination; MoCA, Montreal Cognitive Assessment; TMT-A, Trail Making Test–Part A; TMT-B, Trail Making Test–Part B; VR, Virtual reality.

The subgroup analysis of MMSE scores showed that the following factors were significantly associated with better outcomes: full or semi or non-immersive level (*p* < 0.05), session ≤60 min (*p* < 0.05), frequency > 2 times/week (*p* = 0.0003), participants from Europe (*p* < 0.0001), and male proportion ≤ 40% (*p* = 0.0005) (Table 4; Supplementary Figure 3). Meta-regression analysis indicating that frequency (*p* = 0.019) and male proportion (*p* = 0.019) were significant factors affecting the improvement in MMSE scores (Table 5).

#### 3.3.2 Execution cognition

Patients’ execution cognition was evaluated based on TMT-A and TMT-B. Ten studies reported TMT-A results and fifteen reported TMT-B results. No significant difference was observed between the EG and CG in TMT-A (SMD = −0.26, 95% CI: −0.55 to −0.03, I^2^ = 52%, *p* = 0.08, GRADE: moderate) and TMT-B (SMD = −0.29, 95% CI: −0.64 to 0.07, I^2^ = 80%, *p* = 0.11, GRADE: moderate) (Table 3; Supplementary Figure 4).

The subgroup analysis of TMT-A showed that the following factors were significantly associated with better outcomes: full or semi-immersive level (*p* < 0.05), duration ≤8 weeks (*p* = 0.04), session ≤30 min or > 60 min (*p* < 0.05), education level ≤ 9 years (*p* = 0.0009), and male proportion ≤ 40% (*p* = 0.03) (Table 4, Supplementary Figure 5). Meta-regression analysis indicating that session (*p* = 0.048) and frequency (*p* = 0.040) were significant factors affecting the improvement in TMT-A (Table 5).

The subgroup analysis of TMT-B demonstrated that semi-immersive level (*p* = 0.02) and duration more than 16 weeks (*p* < 0.0001) had significant positive effects on performance (Table 4; Supplementary Figure 6). However, meta-regression analysis showed that immersive level, duration, session, frequency, geographic region, education level, and male proportion were not significant factors influencing improvements in TMT-B outcomes (Table 5).

#### 3.3.3 Attention

DSB and DSF was used to evaluate patients’ attention. Seven and five studies reported DSB and DSF results for 383 and 251 participants, respectively. DSB (SMD = 0.61, 95% CI: 0.21 to 1.02, I^2^ = 72%, *p* = 0.003, GRADE: low) and DSF (SMD = 0.89, 95% CI: 0.34 to 1.45, I^2^ = 75%, *p* = 0.002, GRADE: low) was significantly higher in the EG than in the CG (Table 3; Supplementary Figure 7).

The subgroup analysis of DSB showed that the following factors were significantly associated with better outcomes: semi-immersive level (*p* = 0.0002), 8 < duration ≤16 weeks (*p* = 0.007), 30 < session ≤60 min (*p* < 0.0001), frequency > 2 times/week (*p* = 0.006), participants from Asia and Europe (*p* < 0.05), and male proportion ≤ 40% (*p* = 0.0001) (Table 4; Supplementary Figure 8).

The subgroup analysis of DSF showed that the following factors were significantly associated with better outcomes: semi-immersive level (*p* < 0.0001), duration ≤8 weeks or > 16 weeks (*p* < 0.05), session ≤30 min or > 60 min (*p* < 0.05), participants from Asia and Europe (*p* < 0.05), frequency > 2 times/week and male proportion > 40% (*p* < 0.0001) (Table 4; Supplementary Figure 9).

#### 3.3.4 Memory

Patients’ memory was evaluated based on RAVLT-IR, RAVLT-DR, CVVLT-IR, and CVVLT-DR. There was no difference in RAVLT-IR (SMD = −0.01, 95% CI: −0.38 to 0.36, I^2^ = 57%, *p* = 0.95, GRADE: low), RAVLT-DR (SMD = 0.13, 95% CI: −0.35 to 0.62, I^2^ = 66%, *p* = 0.59, GRADE: low), CVVLT-IR (SMD = 0.13, 95% CI: −0.24 to 0.50, I^2^ = 0%, *p* = 0.49, GRADE: low), and CVVLT-DR (SMD = 0.41, 95% CI: −0.58 to 1.39, I^2^ = 85%, *p* = 0.42, GRADE: very low) between groups (Table 3; Supplementary Figure 10).

Subgroup analysis of RAVLT-IR showed that semi-immersive level (*p* = 0.006) and session >60 min (*p* = 0.006) had a positive effect (Table 4; Supplementary Figure 11).

A subgroup analysis based on immersive level, session, and education level revealed that participants in the EG who engaged in non-immersive level or 30 < session ≤60 min, as well as those with an education level > 9 years, demonstrated significant positive effects on RAVLT-DR (*p* = 0.0003, Table 4; Supplementary Figure 12).

Subgroup analysis of CVVLT-DR showed that education level ≤ 9 years had a positive effect (*p* < 0.0001) (Table 4; Supplementary Figure 13).

#### 3.3.5 Verbal fluency

Animal Word and “ㅅ” Word was used to evaluate patients’ verbal fluency. The meta-analysis revealed that the animal Word (SMD = 0.20, 95% CI: −0.06 to 0.47, I^2^ = 0%, *p* = 0.14, GRADE: moderate) and “ㅅ” Word (SMD = 0.00, 95% CI: −0.48 to 0.47, I^2^ = 0%, *p* = 1.00, GRADE: low) was no difference between groups (Table 3; Supplementary Figure 14).

#### 3.3.6 Visual ability

Two and five studies reported WAIS-BDT and CDT results for 134 and 167 participants to evaluate visual ability, respectively. However, there was no difference in WAIS-BDT (SMD = 0.44, 95% CI: −0.37 to 1.24, I^2^ = 81%, *p* = 0.29, GRADE: very low) and CDT (SMD = 0.21, 95% CI: −0.30 to 0.72, I^2^ = 63%, *p* = 0.41, GRADE: very low) between groups (Table 3; Supplementary Figure 15).

A subgroup analysis based on immersive level, duration, session, and male proportion revealed that participants who engaged in full-immersive level, duration >16 weeks, or session ≤30 min, as well male proportion ≤ 40%, demonstrated significant positive effects on CDT (*p* = 0.002, Table 4; Supplementary Figure 16).

### 3.4 Secondary outcomes and subgroup analysis

#### 3.4.1 Emotional state

GDS-15 and GDS-30 was used to evaluate patients’ emotional state. There was no difference in GDS-15 (SMD = −0.40, 95% CI: −1.17 to −0.37, I^2^ = 85%, *p* = 0.31, GRADE: low) and GDS-30 (SMD = −1.38, 95% CI: −4.51 to 1.76, I^2^ = 98%, *p* = 0.39, GRADE: very low) between EG and CG (Table 3; Supplementary Figure 17).

A subgroup analysis by frequency and geographic region showed that EG of >2 times/week and participants from Europe (*p* < 0.0001) had a positive effect on GDS-15 (Table 4; Supplementary Figure 18).

#### 3.4.2 Quality of life

IADL and QoL-AD was used to evaluate patients’ quality of life. IADL (SMD = 0.22, 95% CI: 0.00 to 0.45, I^2^ = 0%, *p* = 0.049, GRADE: moderate) was significantly higher in the EG than in the CG. There was no difference in QoL-AD (SMD = −0.06, 95% CI: −0.39 to 0.26, I^2^ = 0%, *p* = 0.71, GRADE: low) between groups (Table 3; Supplementary Figure 19).

#### 3.4.3 Dynamic balance

Five and three studies reported TUG and BBS results for 280 and 208 participants to evaluate dynamic balance, respectively. There was no difference in TUG (SMD = 0.05, 95% CI: −0.45 to 0.56, I^2^ = 76%, *p* = 0.83, GRADE: low) and BBS (SMD = 0.45, 95% CI: −0.51 to 1.41, I^2^ = 91%, *p* = 0.35, GRADE: low) between groups (Table 3; Supplementary Figure 20).

Subgroup analysis of TUG showed that full-immersive level (*p* = 0.002) and duration ≤8 weeks or > 16 weeks (*p* < 0.05) had a positive effect (Table 4; Supplementary Figure 21).

A subgroup analysis by immersive level and frequency showed that EG of semi-immersive level and frequency ≤ 2 times/weeks (*p* < 0.0001) had a positive effect on BBS (Table 4; Supplementary Figure 22).

In a word, the summary of optimal VR parameter and demographic factors effects on cognitive domains and dynamic balance are shown in Table 6.

**Table 6:** Summary of optimal VR parameter and demographic factors effects on cognitive domains and dynamic balance.

Outcome	VR parameter				Demographic factors		
	Immersion level	Duration (weeks)	Session (min)	Frequency (times/week)	Geographic region	Education level (years)	Male proportion (%)
Global cognition							
MoCA	Semi	≤ 8,8 < duration ≤16	≤ 30, 30 < session ≤60	> 2	Asia, Europe	-	≤ 40%
MMSE	Full, Semi, Non	≤ 8,8 < duration ≤16, > 16	≤ 30, 30 < session ≤60	> 2	Europe	-	≤ 40%
Execution cognition							
TMT-A	Full, Semi	≤ 8	≤ 30, > 60	-	-	≤ 9	≤ 40%
TMT-B	Semi	> 16	-	-	-	-	-
Attention							
DSB	Semi	8 < duration ≤16	30 < session ≤60	> 2	Asia, Europe	-	≤ 40%
DSF	Semi	≤ 8, > 16	≤ 30, > 60	> 2	Asia, Europe	-	> 40%
Memory							
RAVLT-IR	Semi	-	> 60	-	-	-	-
RAVLT-DR	Non	-	30 < session ≤60	-	-	> 9	-
CVVLT-DR	-	-	-	-	-	≤ 9	-
Visual ability							
CDT	Full	> 16	≤ 30	-	-	-	≤ 40%
Emotional state							
GDS-15	-	-	-	> 2	Europe	-	-
Dynamic balance							
TUG	Full	≤ 8, > 16	-	-	-	-	-
BBS	Semi	-	-	≤ 2	-	-	-

BBS, Berg Balance Scale; CDT, Clock Drawing Test; CVVLT-DR, Chinese Version Verbal Learning Test-Delayed Recall; DSB, Digit Span Backward; DSF, Digit Span Forward; GDS-15, Geriatric Depression Scale-15; MMSE, Mini-Mental State Examination; MoCA, Montreal Cognitive Assessment; RAVLT-DR, Rey Auditory Verbal Learning Test-Delayed Recall; RAVLT-IR, Rey Auditory Verbal Learning Test-Immediate Recall; TMT-A, Trail Making Test–Part A; TMT-B, Trail Making Test–Part B; TUG, Timed Up-and-Go Test; VR, Virtual reality.

### 3.5 Publication bias and sensitivity analysis

**Figure 3:**
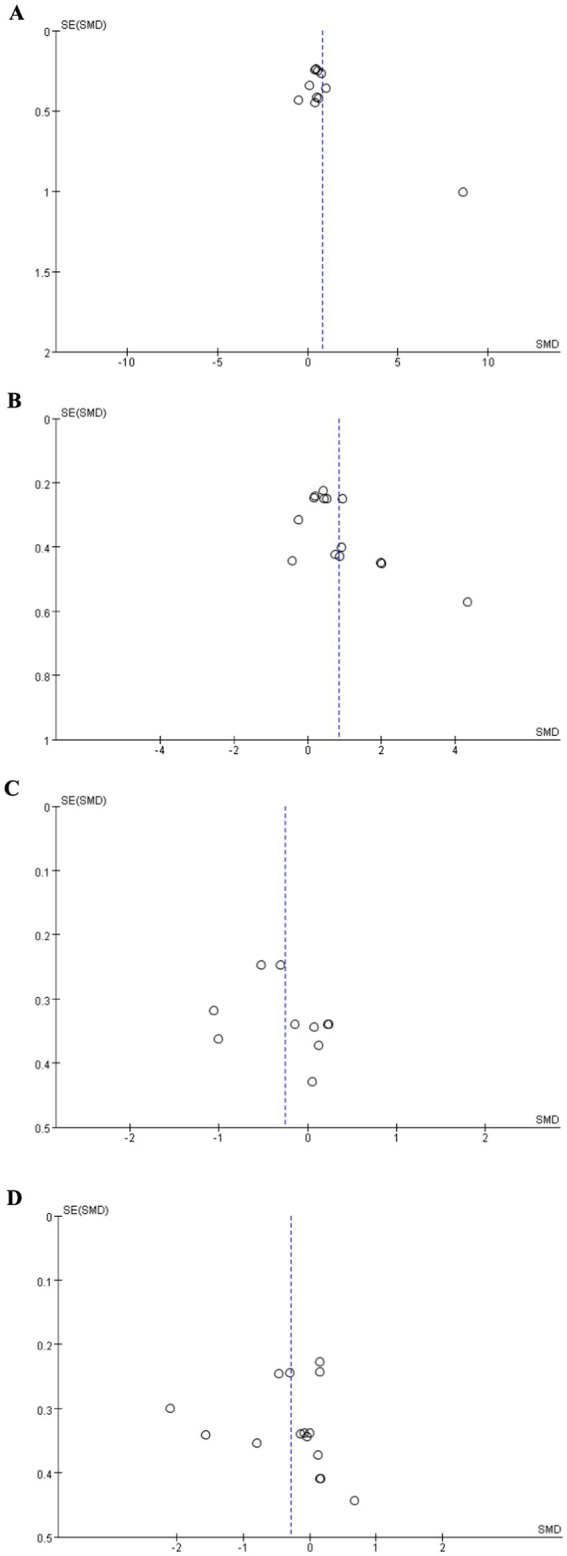
Funnel plot. **(A)** MoCA, **(B)** MMSE, **(C)** TMT-A, **(D)** TMT-B. MMSE, Mini-Mental State Examination; MoCA, Montreal Cognitive Assessment; TMT-A, Trail Making Test–Part A; TMT-B, Trail Making Test–Part B.

Figure 3 showed the funnel plot of MoCA, MMSE, TMT-A, and TMT-B. The results of Egger’s test showed that MoCA (*t* = 2.03, *p* = 0.073), TMT-A (*t* = 0.93, *p* = 0.381), and TMT-B (*t* = 0.16, *p* = 0.872) was no significant publication bias. However, Egger’s test showed that there was publication bias in the MMSE (*t* = 2.55, *p* = 0.026). Pooled results using the “trim and fill” method showed no obvious changes (SMD = 0.872, *p* < 0.0001), indicating this result was robust. Additionally, sensitivity analysis showed that all pooled estimates were not materially altered after removal of a single study.

## 4 Discussion

### 4.1 Main findings

This meta-analysis evaluated the effectiveness of VR-based interventions on cognitive function, emotional state, and quality of life in individuals with MCI. The findings indicate that VR interventions significantly improve global cognition, attention, and quality of life. However, their effects on executive function, memory, visuospatial abilities, emotional state, and dynamic balance appear to be influenced by specific VR parameters, including immersion level, duration, session, and frequency, as well as demographic factors such as geographic region, education level, and male proportion. These results align with previous research (1, 14, 27) supporting VR as a promising tool for cognitive rehabilitation in older adults with cognitive impairments.

### 4.2 Effects on cognitive function

MCI is characterized by cognitive decline and represents a crucial window for AD prevention and treatment (2, 8). This study confirms that VR-based interventions significantly enhance global cognition (MoCA and MMSE scores) and attention (DSB and DSF scores) in individuals with MCI, highlighting VR as a promising non-pharmacological therapy for this population. However, no significant improvements were observed in other cognitive domains, such as executive function, memory, and visuospatial abilities. This finding is consistent with some reviews suggesting that VR interventions do not significantly enhance these cognitive functions (8, 28). In contrast, other studies have indicated that VR, when combined with additional interventions, can improve these domains (17, 29). This discrepancy may stem from differences in study populations—while our analysis focused exclusively on individuals with MCI, previous studies included both MCI and dementia patients, potentially influencing outcomes. Additionally, differences in study design may contribute to these inconsistencies. Our meta-analysis included only RCTs, ensuring higher methodological rigor, whereas other reviews incorporated non-RCTs, increasing the risk of bias. Further research is needed to determine whether VR interventions can effectively enhance executive function, memory, and other cognitive domains in MCI populations.

Subgroup analysis revealed that VR intervention efficacy is influenced by VR parameters such as immersion level, duration, session, and frequency. Among different immersion levels, semi-immersive VR demonstrated the most significant benefits across multiple cognitive outcomes (MoCA, MMSE, TMT-A, TMT-B, DSB, DSF, and RAVLT-IR), consistent with previous findings (14, 21, 28). From a human-computer interaction perspective, its superior effectiveness can be attributed to an optimal balance between engagement and cognitive load (30). Prior research emphasizes that user acceptance and cognitive workload are crucial factors in determining VR intervention efficacy (30). Non-immersive VR may lack sufficient sensory stimulation for meaningful cognitive engagement, whereas fully immersive VR, while providing an enriched environment, may induce discomfort, fatigue, and cybersickness, leading to cognitive overload (31, 32). Semi-immersive VR strikes a balance, maximizing engagement while minimizing adverse effects, making it a promising approach for cognitive rehabilitation in individuals with MCI.

Regarding intervention duration, interventions lasting ≤16 weeks demonstrated the most significant improvements in several cognitive outcomes (e.g., MoCA, TMT-A, DSB). However, longer interventions (> 16 weeks) were more effective for tasks requiring higher-order cognitive functions, such as executive function and visuospatial abilities (e.g., TMT-B, CDT). This may be due to the different cognitive processes targeted by short- and long-term interventions. Shorter programs may enhance general cognitive function through repeated stimulation, while longer interventions allow for the gradual reinforcement of complex cognitive skills (33). However, prolonged interventions may also lead to participant fatigue or reduced adherence, potentially affecting outcomes. Future research should explore the optimal intervention duration to balance effectiveness and user engagement.

When categorized by session and frequency, the most effective interventions involved sessions lasting ≤60 min and occurring more than twice per week, yielding significant improvements across multiple cognitive outcomes (e.g., MoCA, MMSE, DSB). From a cognitive psychology perspective, shorter, more frequent sessions optimize learning by preventing cognitive fatigue and sustaining attention, which enhances memory consolidation and cognitive engagement (34). Frequent training reinforces neural plasticity, strengthens synaptic connections, and aligns with the principles of spaced learning, improving long-term retention and skill transfer (35). This structured approach may be particularly beneficial for individuals with MCI, as consistent cognitive stimulation helps build cognitive resilience while minimizing the risk of mental fatigue, a common concern in aging populations. Prolonged or infrequent sessions, by contrast, may lead to disengagement and suboptimal cognitive gains. These findings suggest that VR interventions should prioritize an optimal balance between session and frequency to maximize cognitive benefits while mitigating the risk of overexertion.

Session and frequency also influenced intervention effectiveness, with the most beneficial interventions involving session lengths of ≤60 min and a frequency of more than twice per week. From a cognitive psychology perspective, shorter, more frequent sessions optimize learning by preventing cognitive fatigue and maintaining attention, thereby improving memory consolidation and engagement (34). Frequent training reinforces neural plasticity and strengthens synaptic connections, aligning with the principles of spaced learning to enhance long-term retention and skill transfer (35). This approach may be particularly advantageous for individuals with MCI, as consistent cognitive stimulation fosters resilience while minimizing mental fatigue. In contrast, prolonged or infrequent sessions may lead to disengagement and suboptimal cognitive gains. These findings suggest that VR interventions should prioritize an optimal balance between session duration and frequency to maximize cognitive benefits while mitigating fatigue.

Subgroup analysis further demonstrated that VR intervention effectiveness varies by demographic factors. Notably, VR-based interventions showed greater efficacy in studies conducted in Asia and Europe and in populations with ≤40% male participants. Regional differences may be influenced by socio-cultural factors such as digital literacy, attitudes toward technology, and access to digital health interventions. Both Asia and Europe have implemented policies and investments that actively support VR development, fostering advancements in hardware and software applications (36). The integration of VR into education and healthcare in these regions has led to greater familiarity and acceptance, potentially enhancing user engagement and intervention effectiveness (36). Additionally, the well-established VR industry in these areas has improved accessibility to high-quality VR systems, further supporting their application in cognitive rehabilitation. Gender differences in VR effectiveness may stem from variations in psychophysiological engagement and behavioral tendencies. Women tend to exhibit greater emotional responsiveness and immersion in virtual environments, which may enhance the impact of VR-based interventions, particularly those utilizing emotional stimuli to reinforce learning. This heightened engagement may lead to stronger cognitive gains. Additionally, behavioral tendencies may play a role—men often prioritize efficiency and task completion speed, whereas women are more likely to immerse themselves in the experience. This aligns well with the design principles of VR-based cognitive training, which emphasize emotional stimulation and deep cognitive processing rather than rapid execution.

### 4.3 Effects on emotional state, quality of life, and dynamic balance

The secondary outcomes revealed varied effects of VR interventions on emotional state, quality of life, and dynamic balance in MCI patients. Significant improvements in emotional state were observed in the EG when using higher frequency (>2 times/week) and in European studies, as reflected by GDS-15 scores. This suggests that regional factors and intervention frequency may influence emotional outcomes. However, no differences were found in GDS-30 scores, suggesting that longer assessments may not fully capture the emotional benefits of VR. In terms of quality of life, significant improvements in IADL scores indicate that VR interventions may enhance daily living activities. These findings align with those of Son et al., who reported similar improvements in MCI and AD patients following VR-based cognitive training (37). However, no significant changes were detected in QoL-AD scores, suggesting that while VR interventions may promote functional independence, their impact on overall quality-of-life perceptions may be limited. Regarding dynamic balance, no significant overall differences were found in TUG or BBS scores. However, subgroup analyses revealed that full-immersive VR interventions and durations of ≤8 weeks or > 16 weeks positively influenced TUG performance. Similarly, semi-immersive VR interventions with lower frequency (≤ 2 times per week) improved BBS scores. These findings suggest that both immersion level and intervention duration play critical roles in enhancing dynamic balance. Future studies should further investigate these factors to optimize intervention strategies.

### 4.4 Limitations

Even though this study has clinical implications, there were several limitations. First, it only included English-language publications, potentially overlooking relevant non-English studies, particularly from East Asia, a major contributor to VR research. Future reviews should incorporate multilingual studies for a more comprehensive perspective. Second, many outcomes relied on subjective self-reported measures (e.g., MoCA, MMSE), which are prone to bias and may not accurately reflect objective cognitive or functional improvements. Future studies should integrate standardized objective assessments, such as biomarkers or physiological metrics, to enhance reliability. Third, most studies lacked long-term follow-up, making it unclear whether VR’s benefits are sustained. Lastly, confounding factors—such as baseline cognitive function, comorbidities, and technology familiarity—were often unaddressed, potentially influencing results. Standardized participant selection and rigorous statistical controls are needed to mitigate these effects. Additionally, publication bias may have influenced our results, as positive findings are more likely to be published, potentially inflating effect sizes. Although we assessed publication bias using funnel plots and Egger’s test, the limited number of studies may have affected the reliability of these analyses. Future research should focus on developing standardized VR intervention protocols to enhance replicability and comparability across studies.

## 5 Conclusion

The findings indicate that VR interventions can significantly improve global cognition, attention, and quality of life in individuals with MCI. Subgroup analyses further revealed that optimal cognitive outcomes were associated with semi-immersive VR, session durations of ≤60 min, intervention frequencies exceeding twice per week, studies conducted in Asia and Europe, and participant groups with a male proportion of ≤40%. Moreover, the study provides valuable insights into secondary outcomes, suggesting that VR interventions may positively impact emotional state and dynamic balance when appropriately tailored to factors such as immersion level, duration, frequency, and other relevant parameters.

## Glossary

AD: Alzheimer’s disease
BBS: Berg Balance Scale
CDT: Clock Drawing Test
CG: Control group
CFQ: Cognitive Failure Questionnaire
CI: Confidence intervals
CVVLT-DR: Chinese Version Verbal Learning Test-Delayed Recall
CVVLT-IR: Chinese Version Verbal Learning Test-Immediate Recall
DSB: Digit Span Backward
DSF: Digit Span Forward
EG: Experimental group
GDS-15: Geriatric Depression Scale-15
GDS-30: Geriatric Depression Scale-30
GRADE: Grading of Recommendations, Assessment, Development, and Evaluation
IADL: Instrumental activities of daily living
MCI: Mild cognitive impairment
M/F: Male/Female
MMSE: Mini-Mental State Examination
MoCA: Montreal Cognitive Assessment
NC: Unrecorded
PRISMA: Preferred Reporting Items for Systematic Reviews and Meta-Analyses
QoL-AD: Quality of life for Alzheimer’s disease
RAVLT-DR: Rey Auditory Verbal Learning Test-Delayed Recall
RAVLT-IR: Rey Auditory Verbal Learning Test-Immediate Recall
RCTs: Randomized controlled trials
SDST: Symbol Digit Substitution Test
SMD: Standardized mean difference
TMT-A: Trail Making Test Part A
TMT-B: Trail Making Test Part B
TUG: Timed Up-and-Go Test
VR: Virtual reality
WAIS-BDT: Wechsler Adult Intelligence Scale-Block Design Test
