# Supplementary material for: Effects of virtual reality-based interventions on cognitive function, emotional state, and quality of life in patients with mild cognitive impairment: a meta-analysis
Source: Front Neurol. 2025 Apr 2;16:1496382. doi: 10.3389/fneur.2025.1496382 (PMC12000106; doi:10.3389/fneur.2025.1496382)
Supplement: Supplementary file 1 [file Supplementary_file_1.docx]

Supplementary Material

Effects of Virtual Reality-Based Interventions on Cognitive Function, Emotional State, and Quality of Life in Patients with Mild Cognitive Impairment: A Meta-Analysis

**Supplementary Table 1.** PRISMA 2020 Main Checklist.

| **Section and Topic** | **Item #** | **Checklist item** |
| --- | --- | --- |
| **TITLE** | | |
| Title | 1 | Identify the report as a systematic review. |
| **ABSTRACT** | | |
| Abstract | 2 | See the PRISMA 2020 for Abstracts checklist. |
| **INTRODUCTION** | | |
| Rationale | 3 | Describe the rationale for the review in the context of existing knowledge. |
| Objectives | 4 | Provide an explicit statement of the objective(s) or question(s) the review addresses. |
| **METHODS** | | |
| Eligibility criteria | 5 | Specify the inclusion and exclusion criteria for the review and how studies were grouped for the syntheses. |
| Information sources | 6 | Specify all databases, registers, websites, organisations, reference lists and other sources searched or consulted to identify studies. Specify the date when each source was last searched or consulted. |
| Search strategy | 7 | Present the full search strategies for all databases, registers and websites, including any filters and limits used. |
| Selection process | 8 | Specify the methods used to decide whether a study met the inclusion criteria of the review, including how many reviewers screened each record and each report retrieved, whether they worked independently, and if applicable, details of automation tools used in the process. |
| Data collection process | 9 | Specify the methods used to collect data from reports, including how many reviewers collected data from each report, whether they worked independently, any processes for obtaining or confirming data from study investigators, and if applicable, details of automation tools used in the process. |
| Data items | 10a | List and define all outcomes for which data were sought. Specify whether all results that were compatible with each outcome domain in each study were sought (e.g. for all measures, time points, analyses), and if not, the methods used to decide which results to collect. |
|  | 10b | List and define all other variables for which data were sought (e.g. participant and intervention characteristics, funding sources). Describe any assumptions made about any missing or unclear information. |
| Study risk of bias assessment | 11 | Specify the methods used to assess risk of bias in the included studies, including details of the tool(s) used, how many reviewers assessed each study and whether they worked independently, and if applicable, details of automation tools used in the process. |
| Effect measures | 12 | Specify for each outcome the effect measure(s) (e.g. risk ratio, mean difference) used in the synthesis or presentation of results. |
| Synthesis methods | 13a | Describe the processes used to decide which studies were eligible for each synthesis (e.g. tabulating the study intervention characteristics and comparing against the planned groups for each synthesis (item #5)). |
|  | 13b | Describe any methods required to prepare the data for presentation or synthesis, such as handling of missing summary statistics, or data conversions. |
|  | 13c | Describe any methods used to tabulate or visually display results of individual studies and syntheses. |
|  | 13d | Describe any methods used to synthesize results and provide a rationale for the choice(s). If meta-analysis was performed, describe the model(s), method(s) to identify the presence and extent of statistical heterogeneity, and software package(s) used. |
|  | 13e | Describe any methods used to explore possible causes of heterogeneity among study results (e.g. subgroup analysis, meta-regression). |
|  | 13f | Describe any sensitivity analyses conducted to assess robustness of the synthesized results. |
| Reporting bias assessment | 14 | Describe any methods used to assess risk of bias due to missing results in a synthesis (arising from reporting biases). |
| Certainty assessment | 15 | Describe any methods used to assess certainty (or confidence) in the body of evidence for an outcome. |
| **RESULTS** | | |
| Study selection | 16a | Describe the results of the search and selection process, from the number of records identified in the search to the number of studies included in the review, ideally using a flow diagram. |
|  | 16b | Cite studies that might appear to meet the inclusion criteria, but which were excluded, and explain why they were excluded. |
| Study characteristics | 17 | Cite each included study and present its characteristics. |
| Risk of bias in studies | 18 | Present assessments of risk of bias for each included study. |
| Results of individual studies | 19 | For all outcomes, present, for each study: (a) summary statistics for each group (where appropriate) and (b) an effect estimate and its precision (e.g. confidence/credible interval), ideally using structured tables or plots. |
| Results of syntheses | 20a | For each synthesis, briefly summarise the characteristics and risk of bias among contributing studies. |
|  | 20b | Present results of all statistical syntheses conducted. If meta-analysis was done, present for each the summary estimate and its precision (e.g. confidence/credible interval) and measures of statistical heterogeneity. If comparing groups, describe the direction of the effect. |
|  | 20c | Present results of all investigations of possible causes of heterogeneity among study results. |
|  | 20d | Present results of all sensitivity analyses conducted to assess the robustness of the synthesized results. |
| Reporting biases | 21 | Present assessments of risk of bias due to missing results (arising from reporting biases) for each synthesis assessed. |
| Certainty of evidence | 22 | Present assessments of certainty (or confidence) in the body of evidence for each outcome assessed. |
| **DISCUSSION** | | |
| Discussion | 23a | Provide a general interpretation of the results in the context of other evidence. |
|  | 23b | Discuss any limitations of the evidence included in the review. |
|  | 23c | Discuss any limitations of the review processes used. |
|  | 23d | Discuss implications of the results for practice, policy, and future research. |
| **OTHER INFORMATION** | | |
| Registration and protocol | 24a | Provide registration information for the review, including register name and registration number, or state that the review was not registered. |
|  | 24b | Indicate where the review protocol can be accessed, or state that a protocol was not prepared. |
|  | 24c | Describe and explain any amendments to information provided at registration or in the protocol. |
| Support | 25 | Describe sources of financial or non-financial support for the review, and the role of the funders or sponsors in the review. |
| Competing interests | 26 | Declare any competing interests of review authors. |
| Availability of data, code and other materials | 27 | Report which of the following are publicly available and where they can be found: template data collection forms; data extracted from included studies; data used for all analyses; analytic code; any other materials used in the review. |

**Supplementary Table 2.** Search strategy on PubMed, Embase, Elsevier, Web of Science, and SciELO

| **Search** | **Item** |
| --- | --- |
| #1 | (mild cognitive impairment or patients with mild cognitive impairment or mild cognition impairment or mild cognitive impairment identification or early mild cognitive impairment or lightly cognitive impediment or mild cognitive disorders or mild cognitive or Cognitive Dysfunctions or Cognitive Impairments or Cognitive Impairment or Cognitive Disorder or Cognitive Disorders or Mild Cognitive Impairment or Mild Cognitive Impairments or Cognitive Decline or Cognitive Declines or Mental Deterioration or Mental Deteriorations or memory disorder or memory impairment) |
| #2 | (Virtual Reality or immersive virtual reality or Non-immersive virtual reality or Virtual reality or virtual reality modeling or visual reality or virtual reality technology or virtual realization or virtual or virtue reality or virtual environment or educational Virtual Realities or Educational Virtual Reality or Instructional Virtual Realities or Instructional Virtual Reality or Virtual Reality Immersion Therapy or Virtual Reality Therapy or Virtual Reality Therapies or immersive virtual environment) |
| #3 | (cognitive function or global cognition or execution function or attention or memory or verbal fluency or visual ability or emotional state or quality of life or dynamic balance or Montreal Cognitive Assessment or MoCA or Mini-mental State Examination or MMSE or Symbol Digit Substitution Test or Cognitive Failure Questionnaire or Trail Making Test–Part A or TMT-A or Trail Making Test–Part B or TMT-B or Digit Span Backward or Digit Span Forward or Rey Auditory Verbal Learning Test-Immediate Recall or Rey Auditory Verbal Learning Test-Delayed Recall or Chinese Version Verbal Learning Test-Immediate Recall or Chinese Version Verbal Learning Test-Delayed Recall or Animal Word or “ㅅ” Word or Wechsler Adult Intelligence Scale-Block Design Test or Clock Drawing Test or Geriatric Depression Scale or Instrumental Activity of Daily Living or IADL or Quality of Life-Alzheimer Disease or Timed Up-and-Go Test or TUG or Berg Balance Scale or BBS) |
| #4 | 1 AND 2 AND 3 |

**Supplementary Table 3.** Grading of Recommendations, Assessments, Developments and Evaluations (GRADE) approach for certainty in evidence

| **Certainty assessment** | | | | | | | **№ of patients** | | **Effect** | | **Certainty** | **Importance** |
| --- | --- | --- | --- | --- | --- | --- | --- | --- | --- | --- | --- | --- |
| **№ of studies** | **Study design** | **Risk of bias** | **Inconsistency** | **Indirectness** | **Imprecision** | **Other considerations** | **EG** | **CG** | **Relative (95% CI)** | **SMD (95% CI)** |  |  |
| **MoCA** | | | | | | | | | | | | |
| 11 | randomised trials | not serious | serious | not serious | not serious | none | 232 | 234 | - | SMD 0.82 (0.27 to 1.38) | ⨁⨁⨁◯ Moderate | CRITICAL |
| **MMSE** | | | | | | | | | | | | |
| 14 | randomised trials | serious | serious | not serious | not serious | none | 328 | 333 | - | SMD 0.83 (0.40 to 1.26) | ⨁⨁◯◯ Low | CRITICAL |
| **SDST** | | | | | | | | | | | | |
| 2 | randomised trials | serious | serious | not serious | serious | none | 67 | 67 | - | SMD 1.14 (-0.50 to 2.78) | ⨁◯◯◯ Very Low | CRITICAL |
| **CFQ** | | | | | | | | | | | | |
| 3 | randomised trials | serious | not serious | not serious | serious | none | 80 | 80 | - | SMD -0.09 (-0.40 to 0.22) | ⨁⨁◯◯ Low | CRITICAL |
| **TMT-A** | | | | | | | | | | | | |
| 10 | randomised trials | not serious | serious | not serious | not serious | none | 208 | 197 | - | SMD -0.26 (-0.55 to -0.03) | ⨁⨁⨁◯ Moderate | CRITICAL |
| **TMT-B** | | | | | | | | | | | | |
| 15 | randomised trials | not serious | serious | not serious | not serious | none | 338 | 334 | - | SMD -0.29 (-0.64 to 0.07) | ⨁⨁⨁◯ Moderate | CRITICAL |
| **DSB** | | | | | | | | | | | | |
| 7 | randomised trials | serious | serious | not serious | not serious | none | 189 | 194 | - | SMD 0.61 (0.21 to 1.02) | ⨁⨁◯◯ Low | CRITICAL |
| **DSF** | | | | | | | | | | | | |
| 5 | randomised trials | serious | serious | not serious | not serious | none | 123 | 128 | - | SMD 0.89 (0.34 to 1.45) | ⨁⨁◯◯ Low | CRITICAL |
| **RAVLT-IR** | | | | | | | | | | | | |
| 6 | randomised trials | serious | serious | not serious | not serious | none | 139 | 143 | - | SMD -0.01 (-0.38 to 0.36) | ⨁⨁◯◯ Low | CRITICAL |
| **RAVLT-DR** | | | | | | | | | | | | |
| 5 | randomised trials | serious | serious | not serious | not serious | none | 100 | 104 | - | SMD 0.13 (-0.35 to 0.62) | ⨁⨁◯◯ Low | CRITICAL |
| **CVVLT-IR** | | | | | | | | | | | | |
| 3 | randomised trials | serious | not serious | not serious | serious | none | 60 | 56 | - | SMD 0.13 (-0.24 to 0.50) | ⨁⨁◯◯ Low | CRITICAL |
| **CVVLT-DR** | | | | | | | | | | | | |
| 3 | randomised trials | serious | serious | not serious | serious | none | 60 | 56 | - | SMD 0.41 (-0.58 to 1.39) | ⨁◯◯◯ Very Low | CRITICAL |
| **Animal word** | | | | | | | | | | | | |
| 5 | randomised trials | serious | not serious | not serious | not serious | none | 110 | 107 | - | SMD 0.20 (-0.06 to 0.47) | ⨁⨁⨁◯ Moderate | CRITICAL |
| **“ㅅ” word** | | | | | | | | | | | | |
| 3 | randomised trials | serious | not serious | not serious | serious | none | 34 | 35 | - | SMD -0.00 (-0.48 to 0.47) | ⨁⨁◯◯ Low | CRITICAL |
| **WAIS-BDT** | | | | | | | | | | | | |
| 2 | randomised trials | serious | serious | not serious | serious | none | 67 | 67 | - | SMD 0.44 (-0.37 to 1.24) | ⨁◯◯◯ Very Low | CRITICAL |
| **CDT** | | | | | | | | | | | | |
| 5 | randomised trials | serious | serious | not serious | serious | none | 84 | 83 | - | SMD 0.21 (-0.30 to 0.72) | ⨁◯◯◯ Very Low | CRITICAL |
| **GDS-15** | | | | | | | | | | | | |
| 5 | randomised trials | serious | serious | not serious | not serious | none | 102 | 101 | - | SMD -0.40 (-1.17 to 0.37) | ⨁⨁◯◯ Low | IMPORTANT |
| **GDS-30** | | | | | | | | | | | | |
| 2 | randomised trials | serious | serious | not serious | serious | none | 55 | 57 | - | SMD -1.38 (-4.51 to 1.76) | ⨁◯◯◯ Very Low | IMPORTANT |
| **IADL** | | | | | | | | | | | | |
| 7 | randomised trials | serious | not serious | not serious | not serious | none | 155 | 151 | - | SMD 0.22 (-0.00 to 0.45) | ⨁⨁⨁◯ Moderate | IMPORTANT |
| **QoL-AD** | | | | | | | | | | | | |
| 4 | randomised trials | serious | not serious | not serious | serious | none | 77 | 69 | - | SMD -0.06 (-0.39 to 0.26) | ⨁⨁◯◯ Low | IMPORTANT |
| **TUG** | | | | | | | | | | | | |
| 5 | randomised trials | serious | serious | not serious | not serious | none | 142 | 138 | - | SMD 0.05 (-0.45 to 0.56) | ⨁⨁◯◯ Low | IMPORTANT |
| **BBS** | | | | | | | | | | | | |
| 3 | randomised trials | serious | serious | not serious | not serious | none | 106 | 102 | - | SMD 0.45 (-0.51 to 1.41) | ⨁⨁◯◯ Low | IMPORTANT |


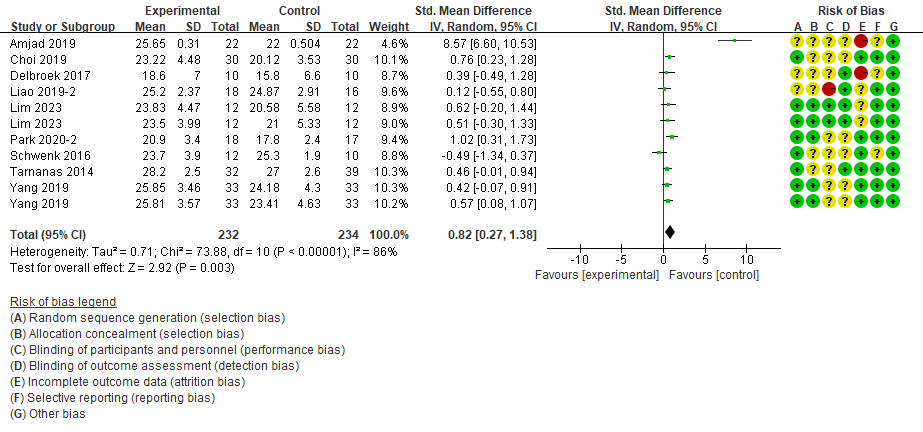


**A -** **MoCA**


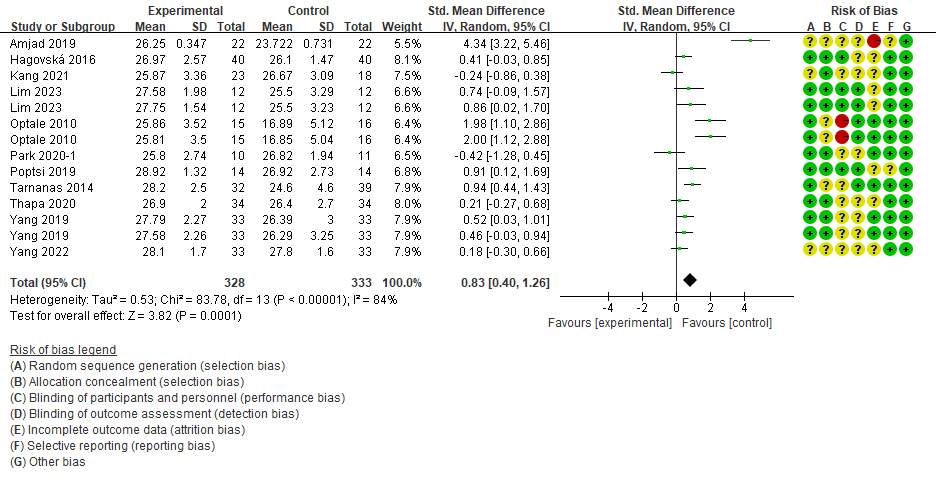


**B -** **MMSE**


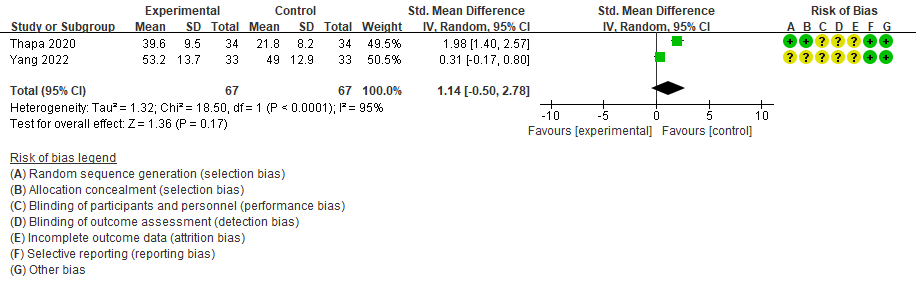


**C –** **SDST**


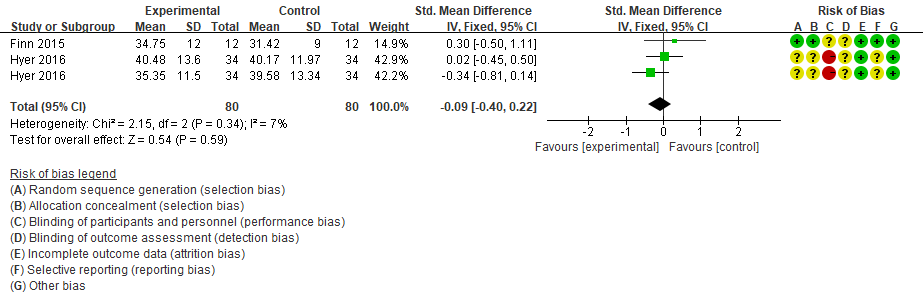


**D –** **CFQ**

**Supplementary Figure 1.** Forest plot of global cognition.


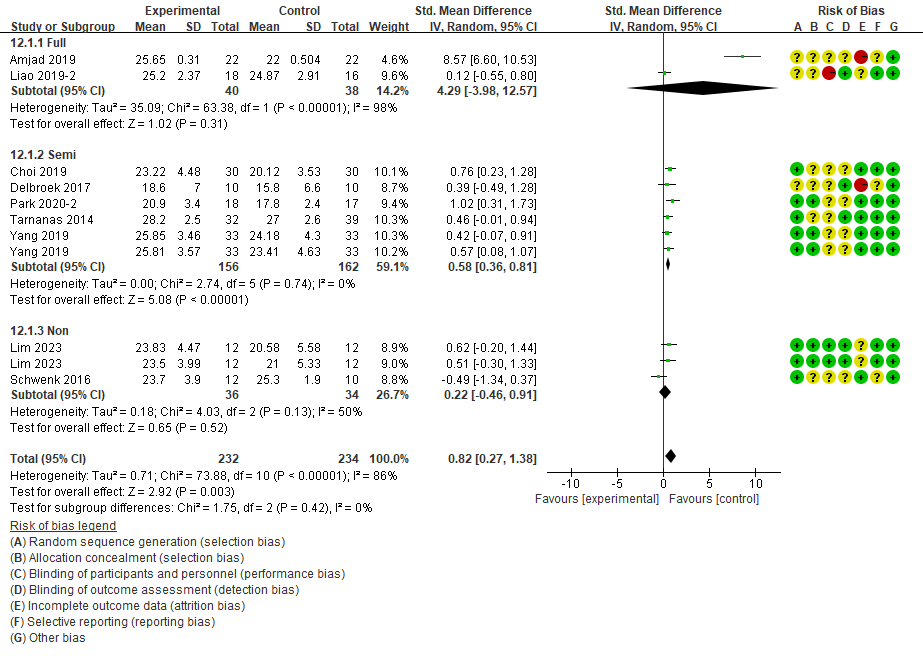


**A – Subgroup analysis by Immersion Level**

**
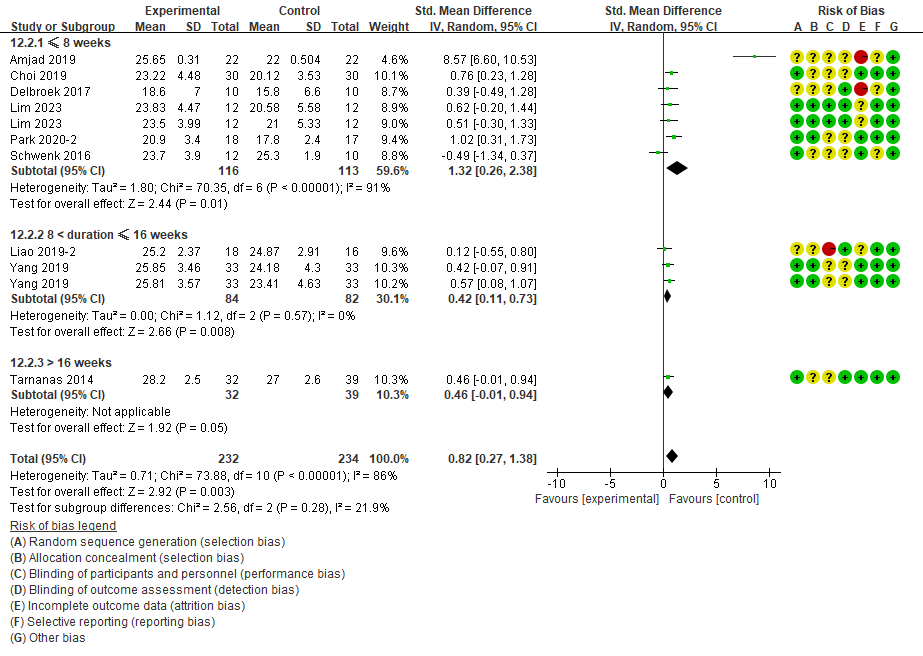
**

**B – Subgroup analysis by Duration**

**
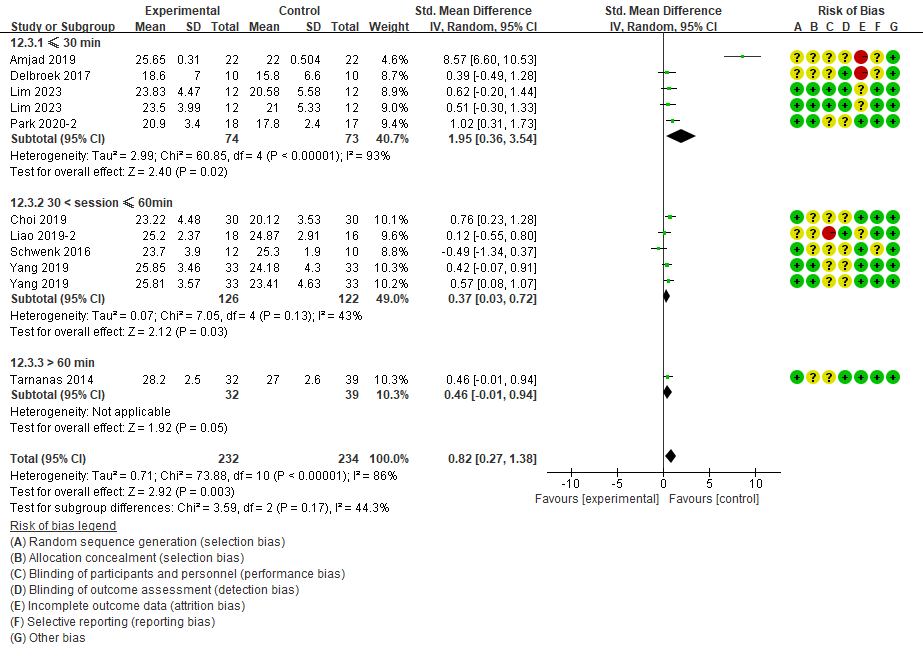
**

**C – Subgroup analysis by Session**

**
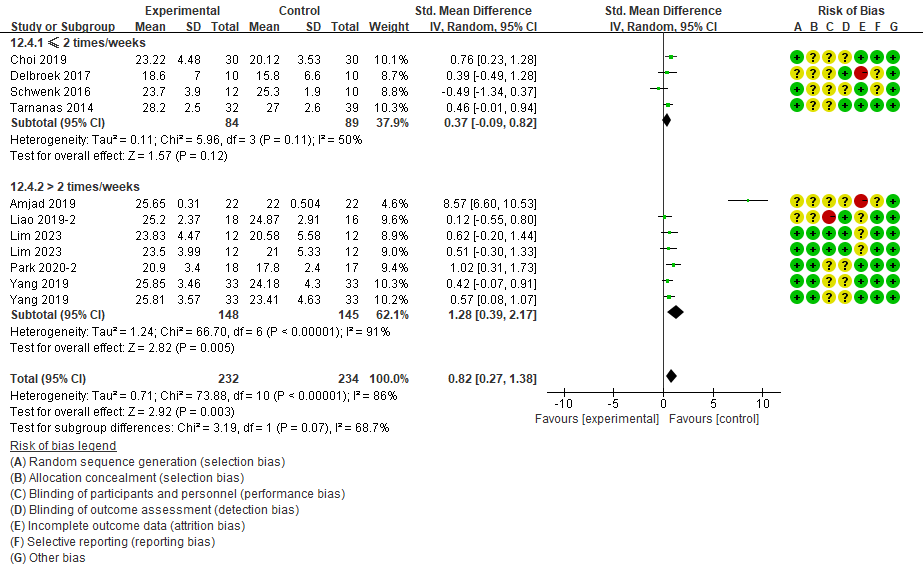
**

**D – Subgroup analysis by Frequency**

**
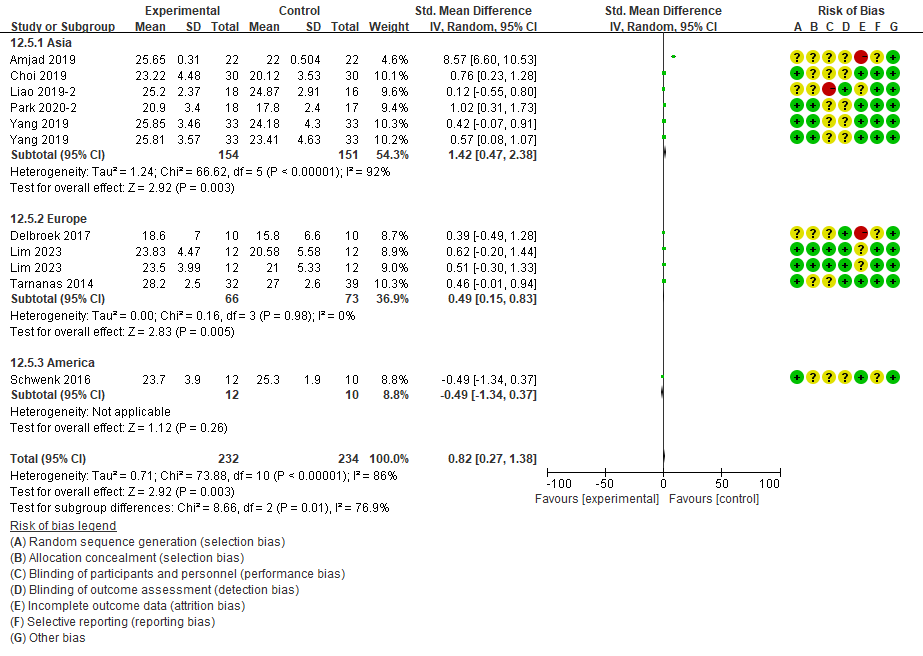
**

**E – Subgroup analysis by Geographic Region**

**
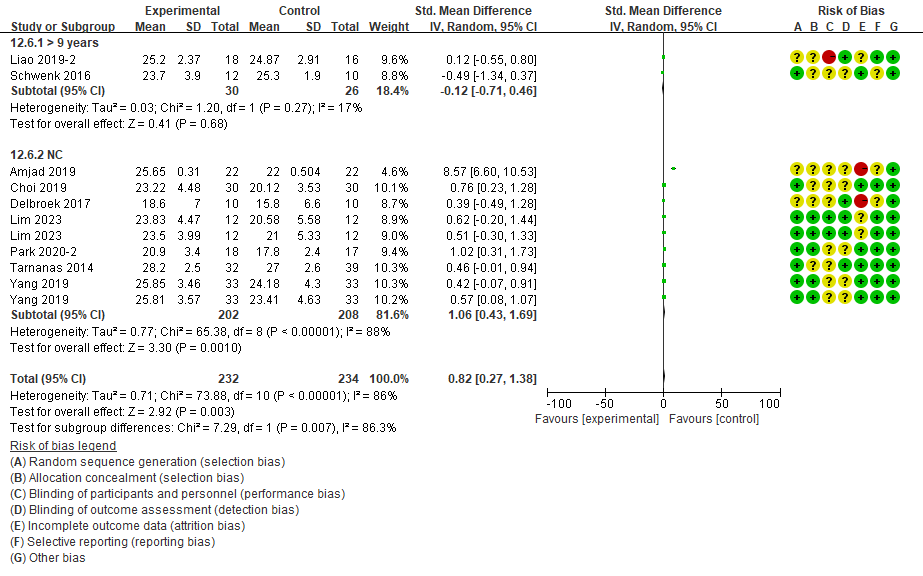
**

**F – Subgroup analysis by Education Level**

**
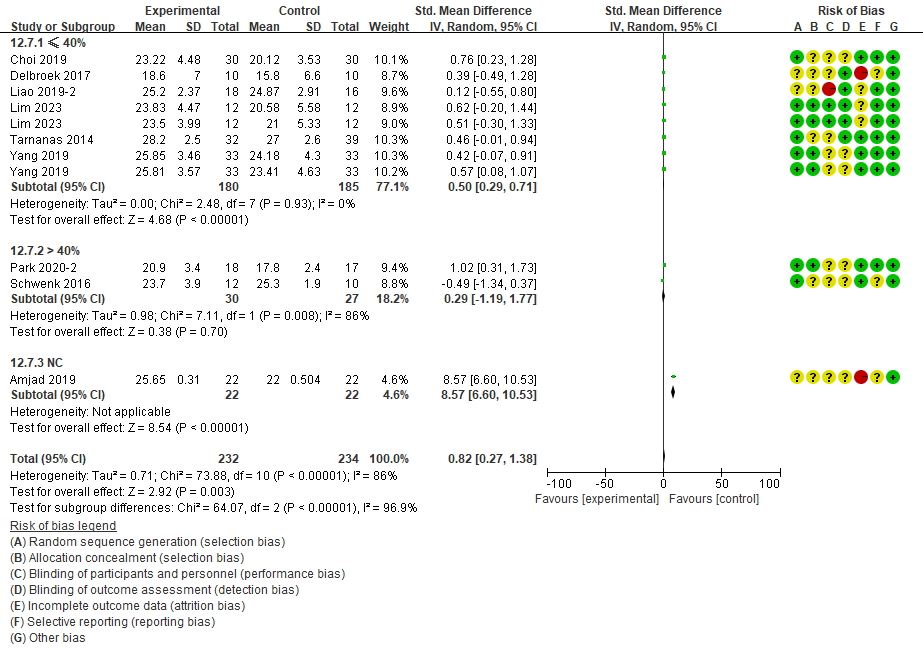
**

**G – Subgroup analysis by** **Male Proportion**

**Supplementary Figure 2.** Forest plot of subgroup analysis of MoCA.

**
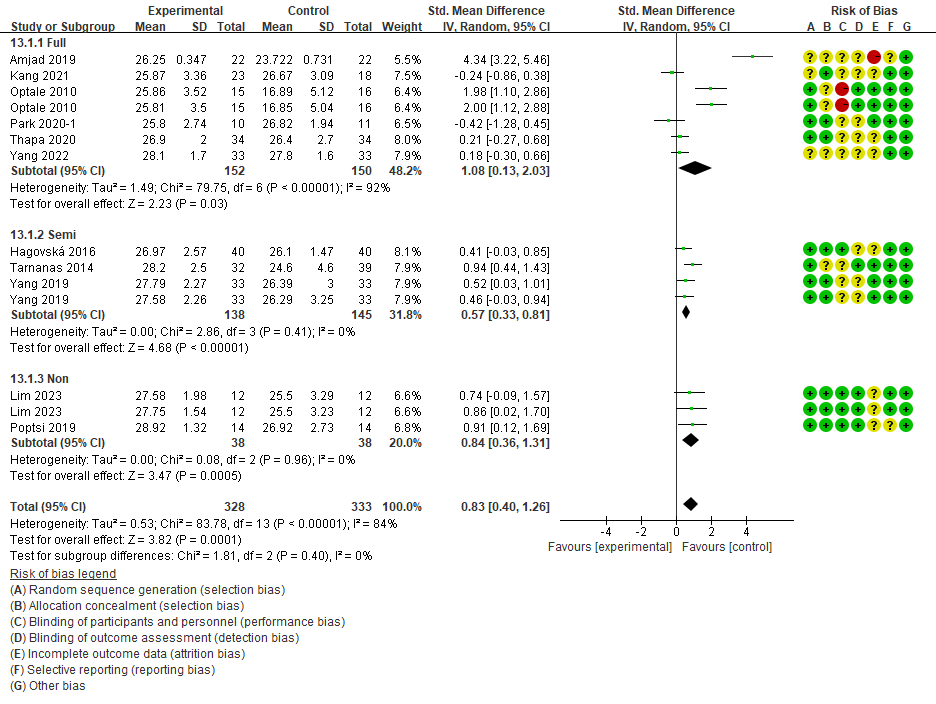
**

**A – Subgroup analysis by Immersion Level**

**
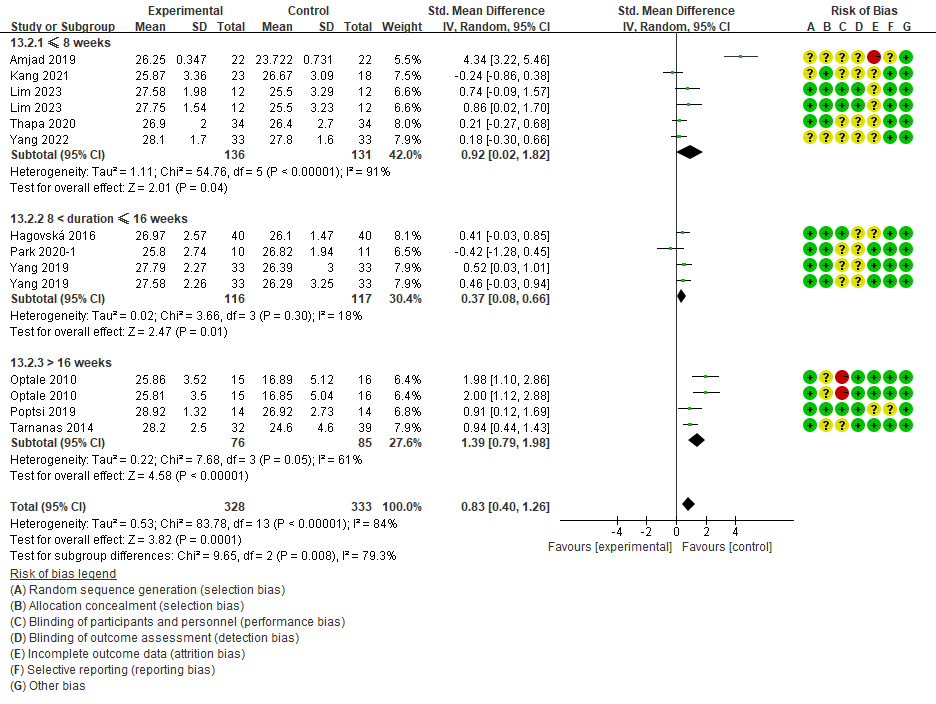
**

**B – Subgroup analysis by Duration**

**
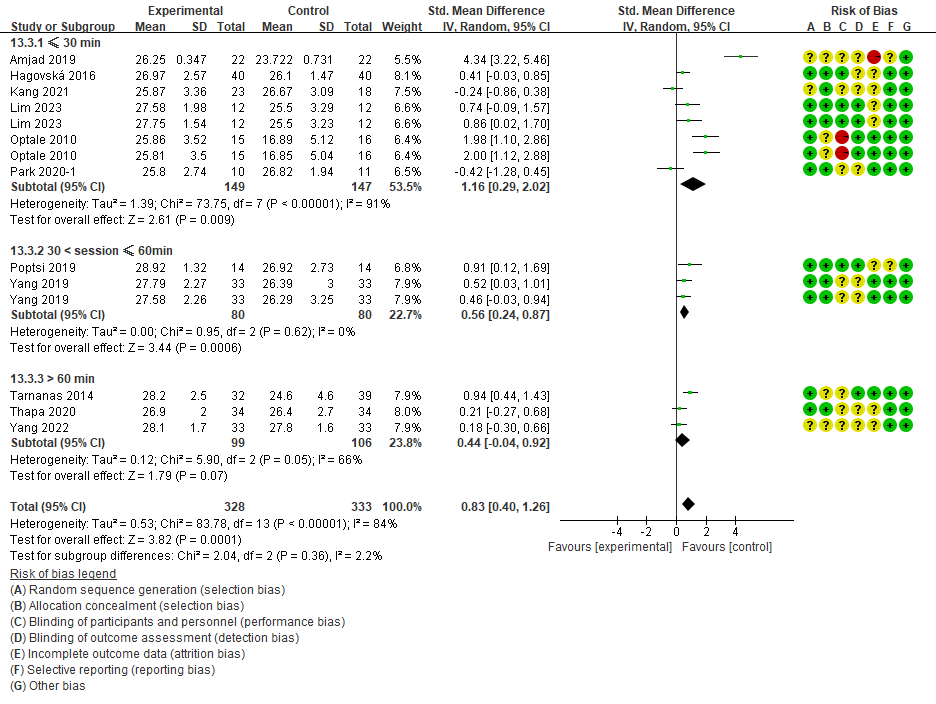
**

**C – Subgroup analysis by Session**


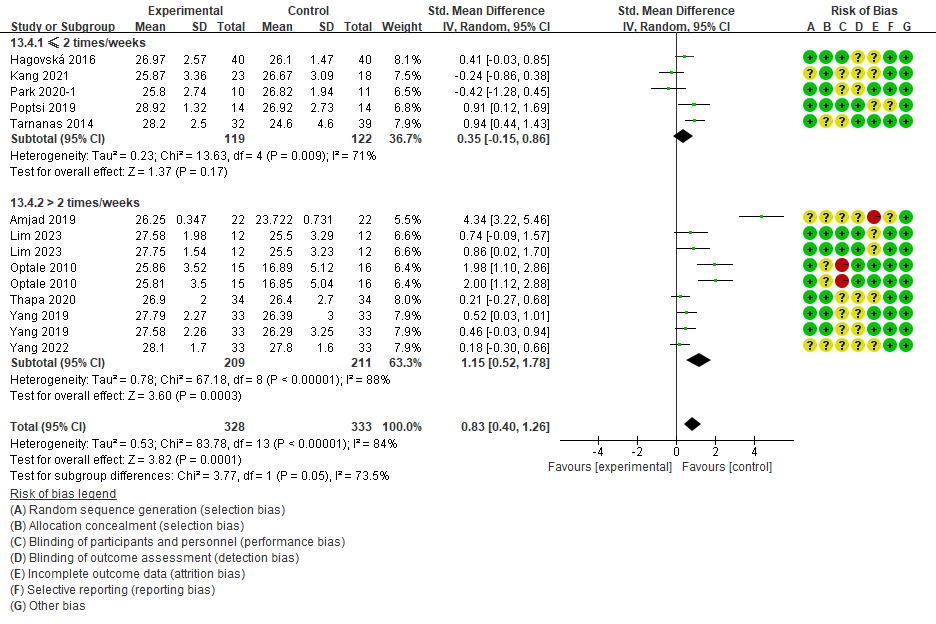


**D – Subgroup analysis by Frequency**

**
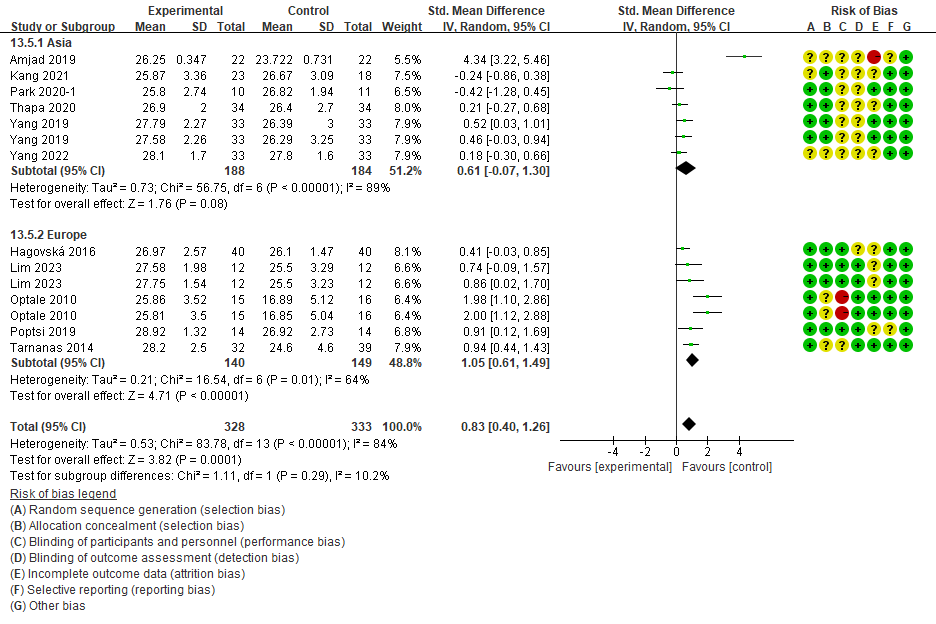
**

**E – Subgroup analysis by Geographic Region**

**
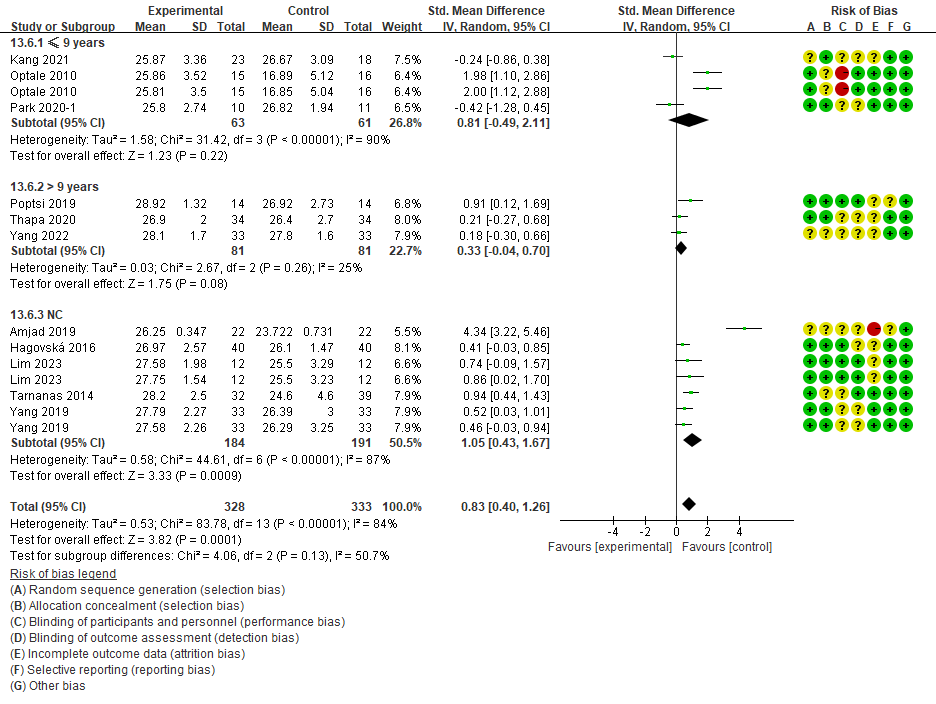
**

**F – Subgroup analysis by Education Level**

**
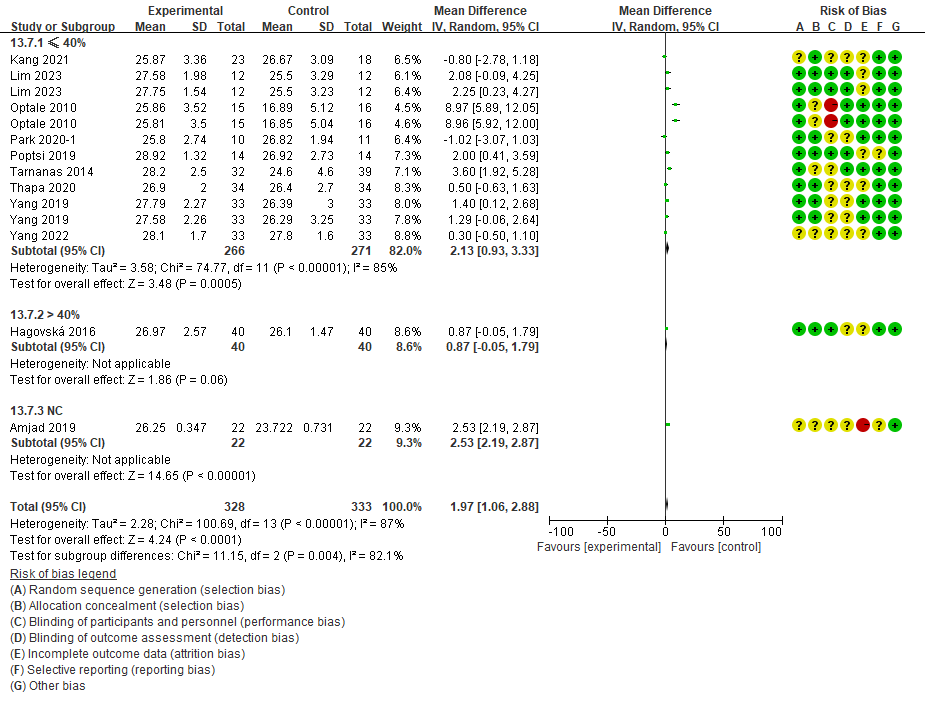
**

**G – Subgroup analysis by Male Proportion**

**Supplementary Figure 3.** Forest plot of subgroup analysis of MMSE.

**
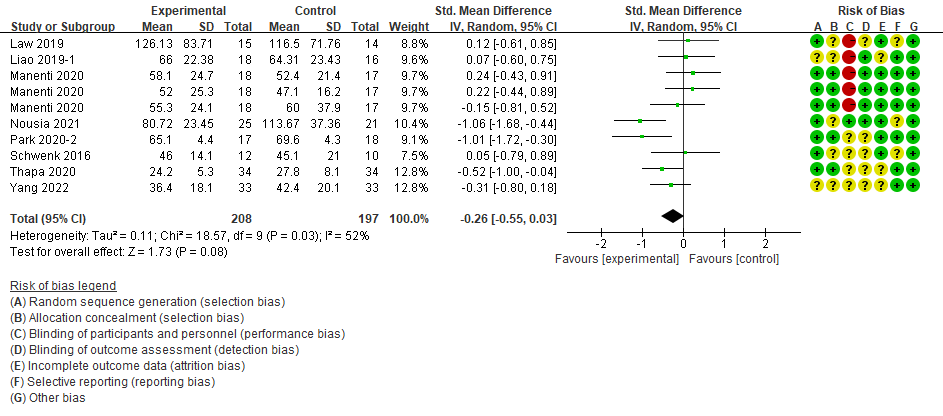
**

**A –** **TMT-A**

**
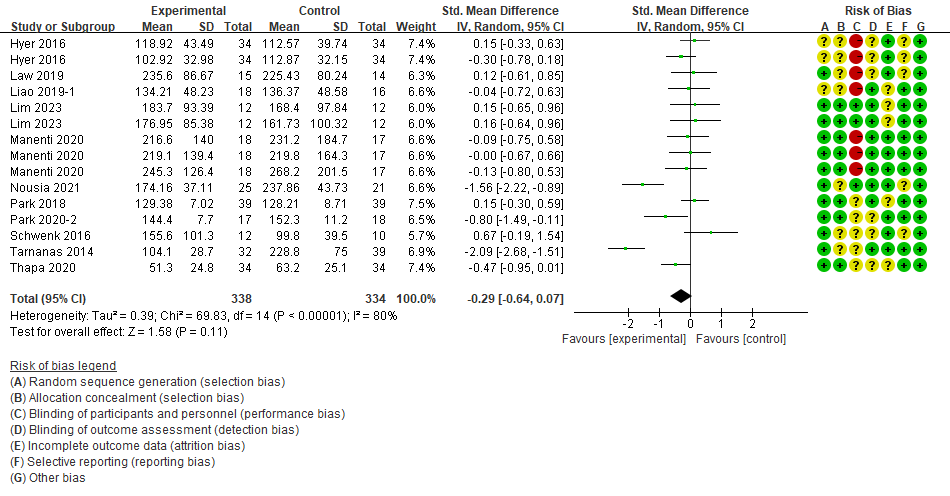
**

**B –** **TMT-B**

**Supplementary Figure 4.** Forest plot of execution function.


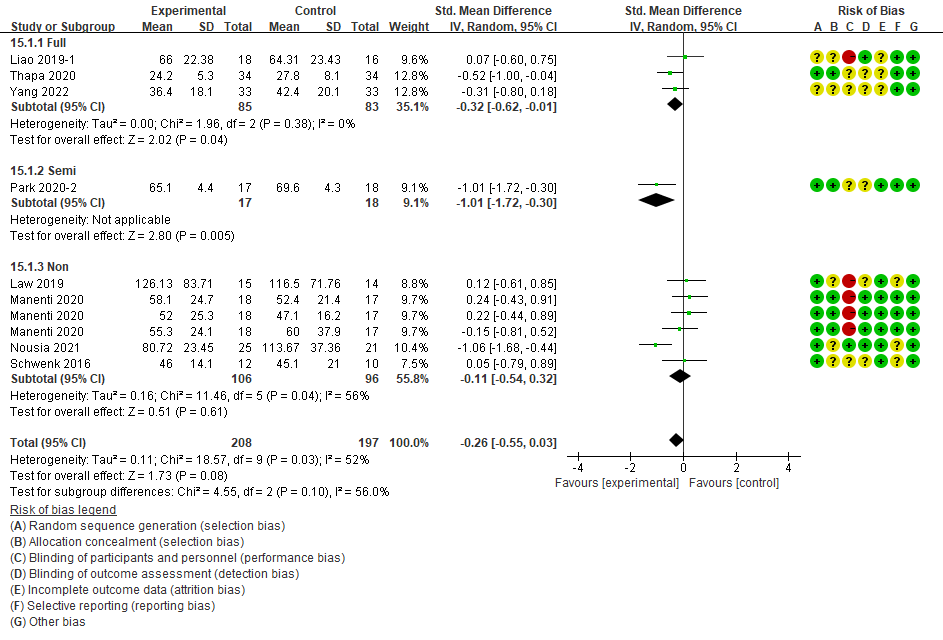


**A – Subgroup analysis by Immersion Level**


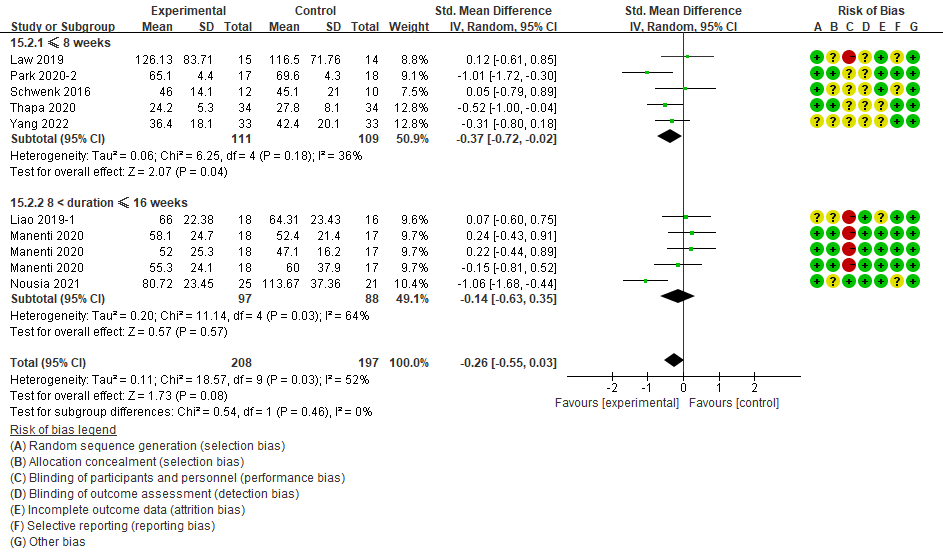


**B – Subgroup analysis by Duration**


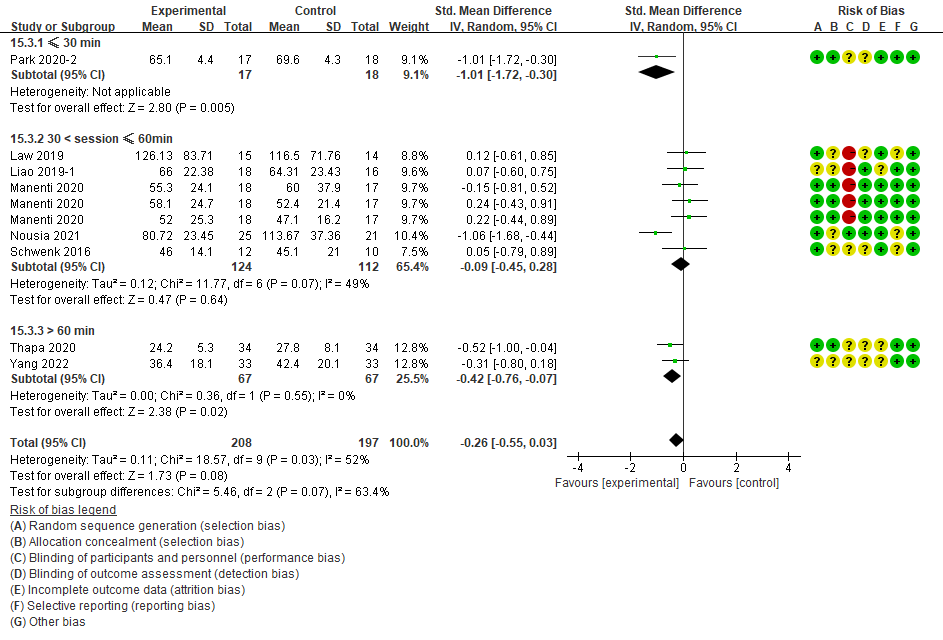


**C – Subgroup analysis by Session**


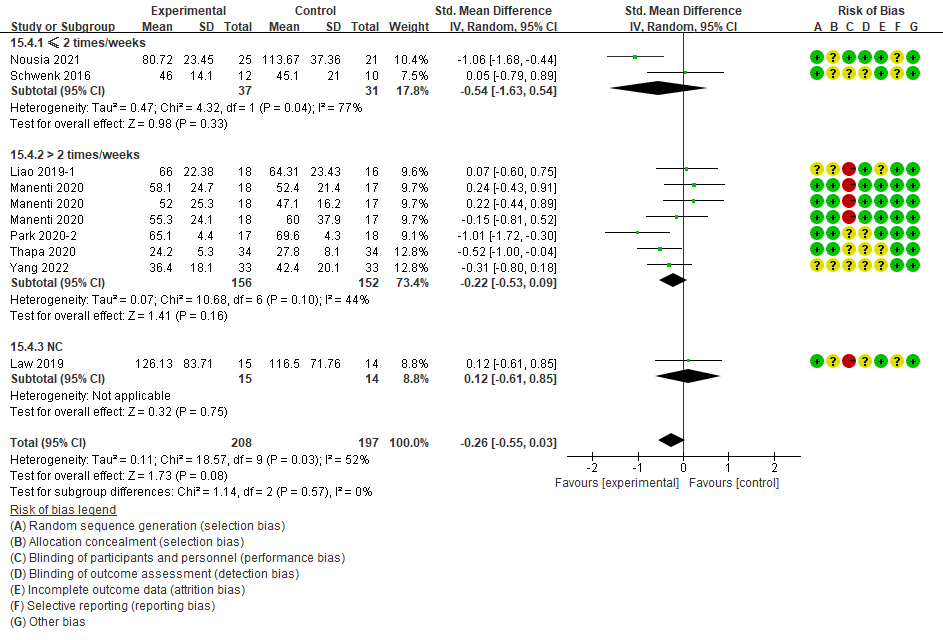


**D – Subgroup analysis by Frequency**


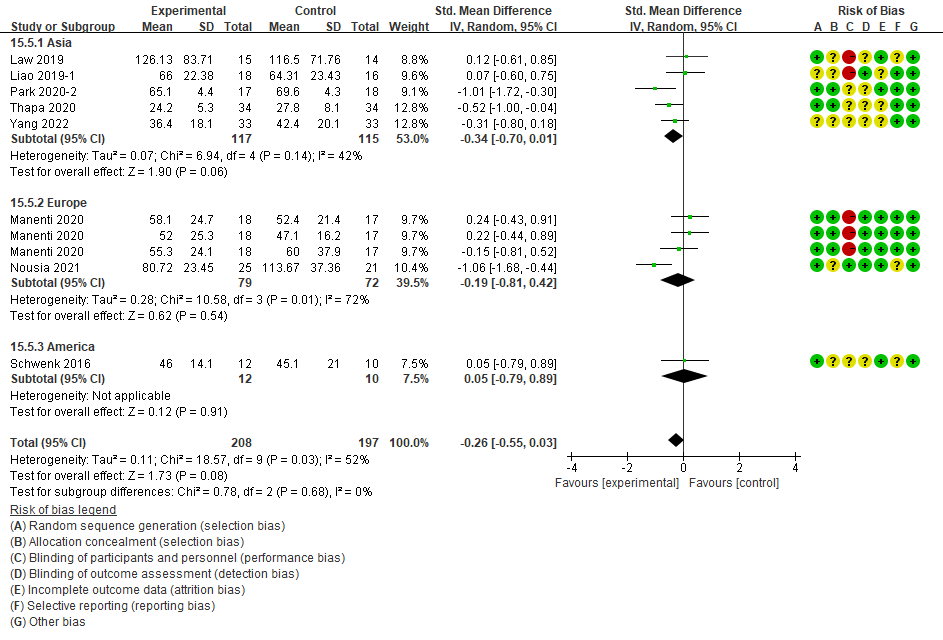


**E – Subgroup analysis by Geographic Region**


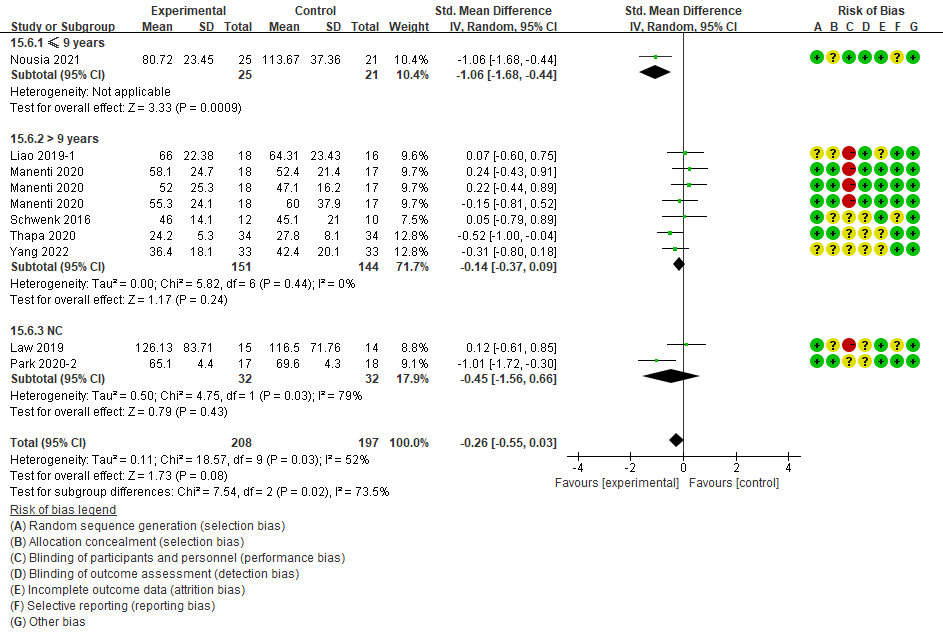


**F – Subgroup analysis by Education Level**


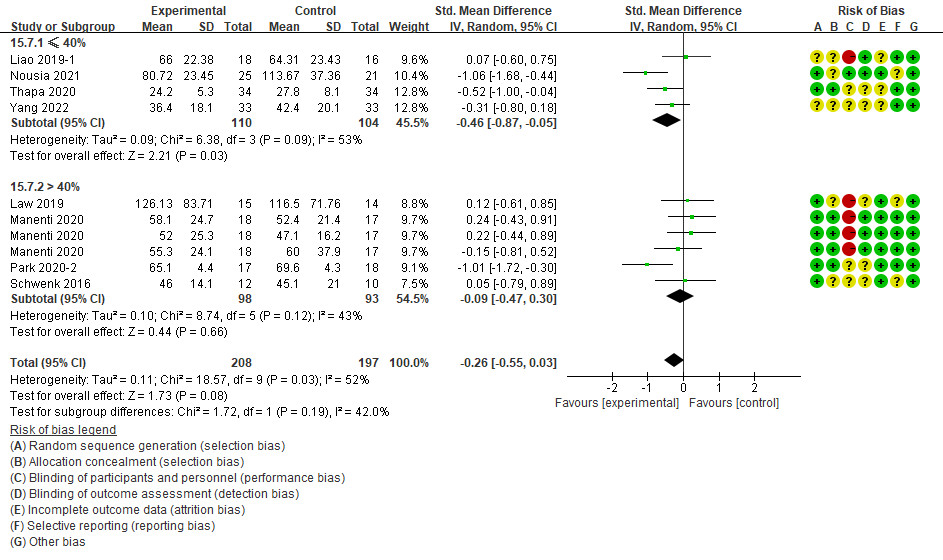


**G – Subgroup analysis by Male Proportion**

**Supplementary Figure 5.** Forest plot of subgroup analysis of TMT-A.

**
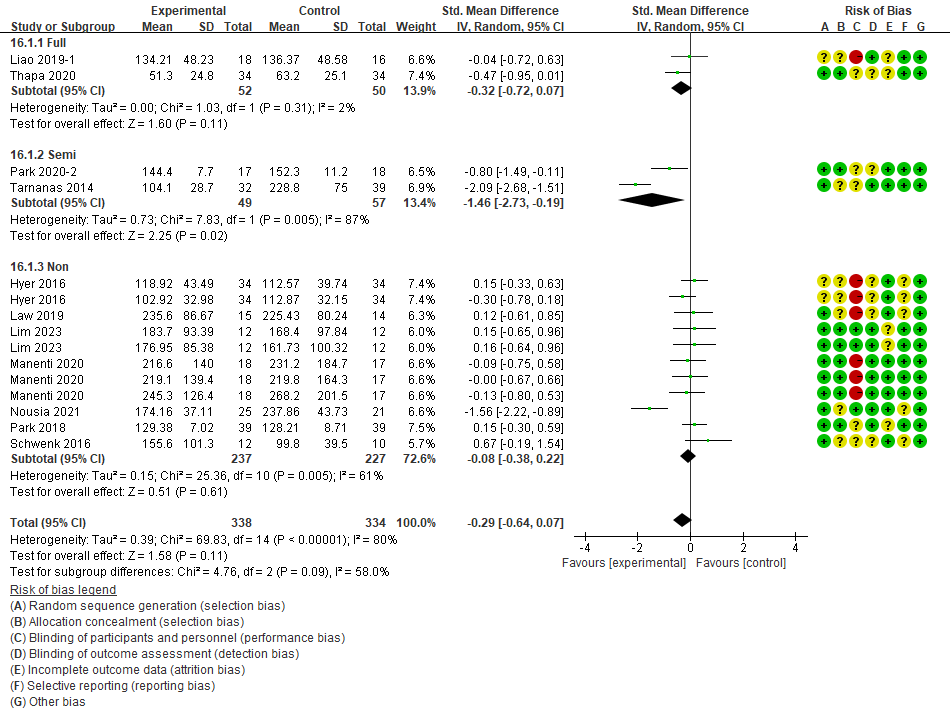
**

**A – Subgroup analysis by Immersion Level**

**
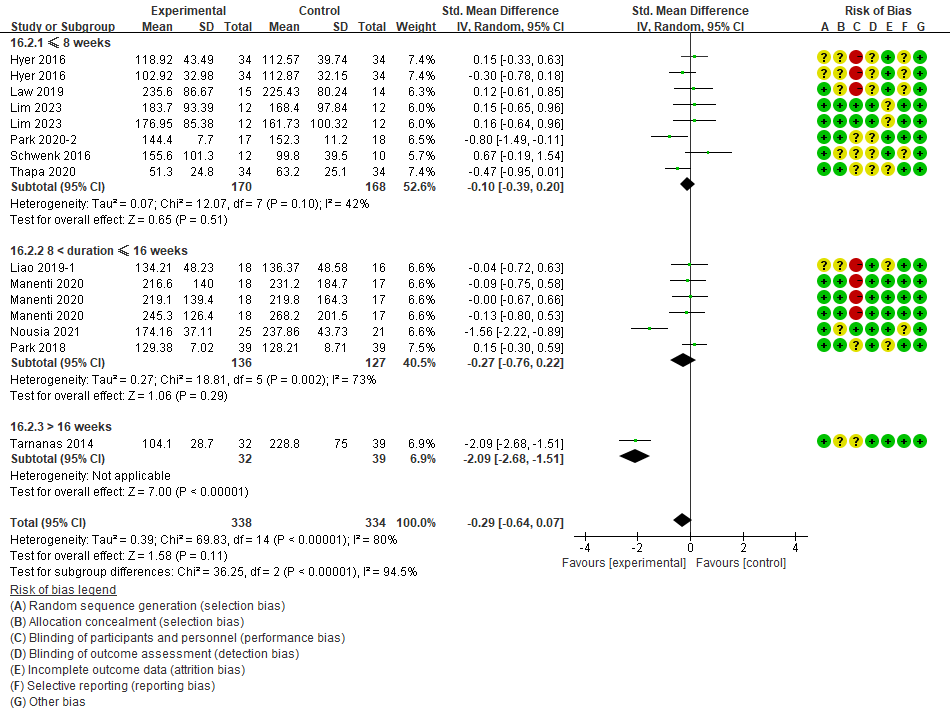
**

**B – Subgroup analysis by Duration**

**
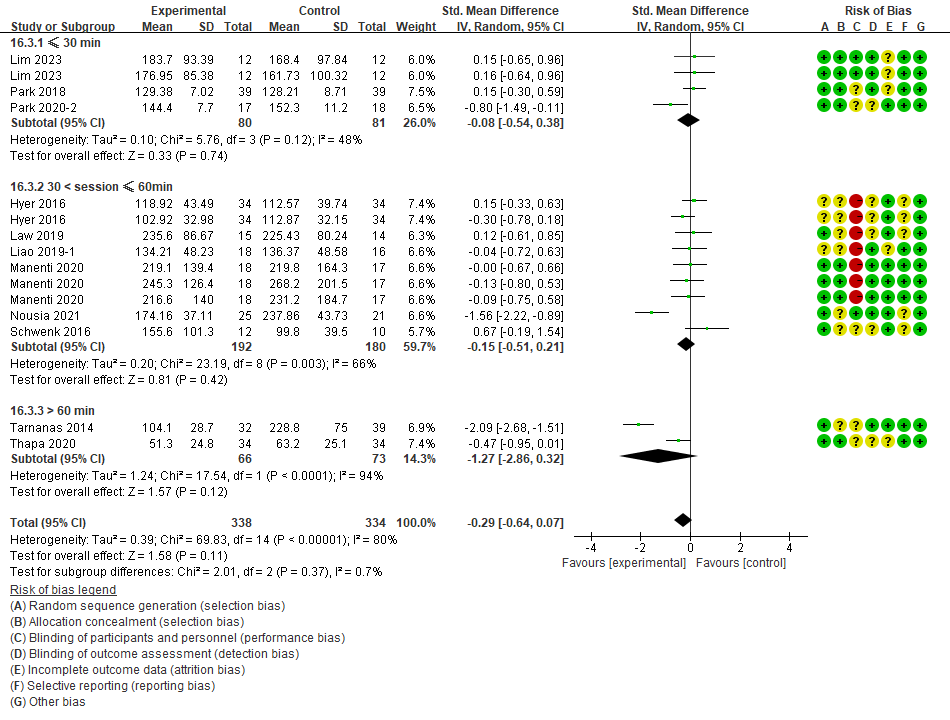
**

**C – Subgroup analysis by Session**

**
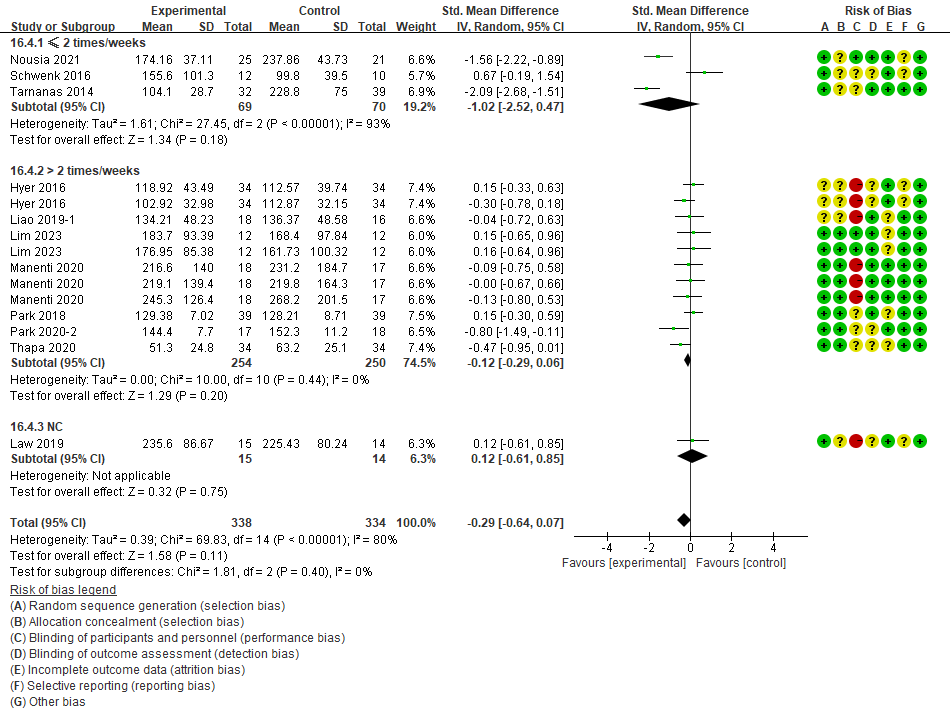
**

**D – Subgroup analysis by Frequency**

**
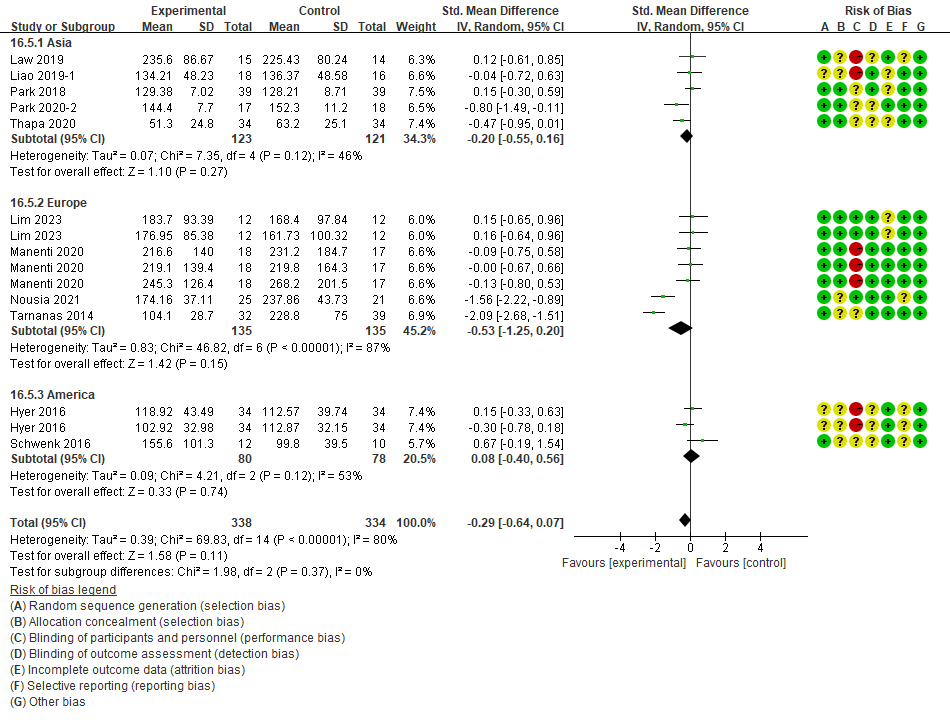
**

**E – Subgroup analysis by Geographic Region**

**
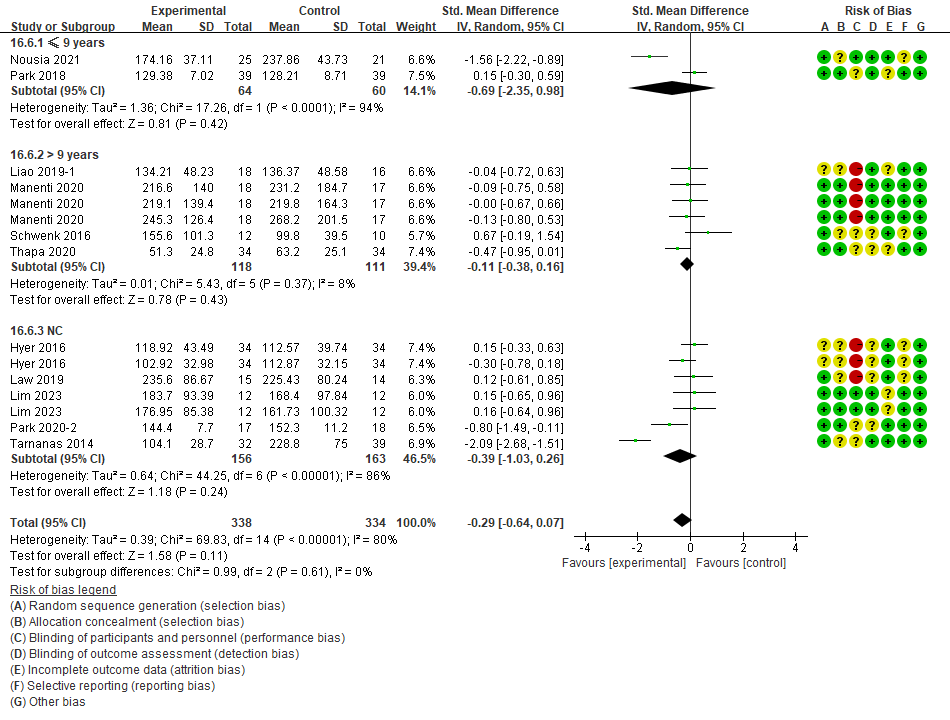
**

**F – Subgroup analysis by Education Level**

**
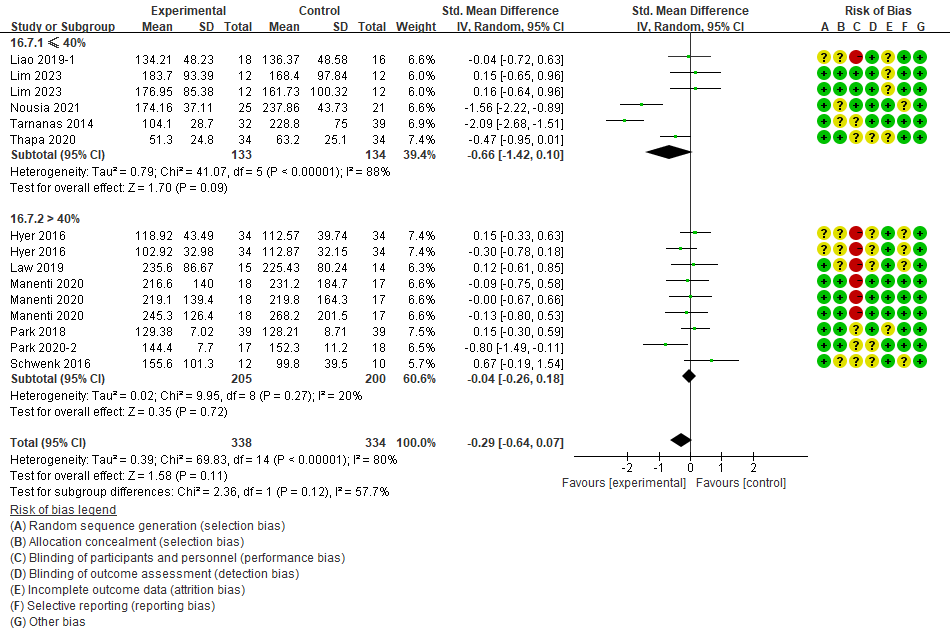
**

**G – Subgroup analysis by Male Proportion**

**Supplementary Figure 6.** Forest plot of subgroup analysis of TMT-B.


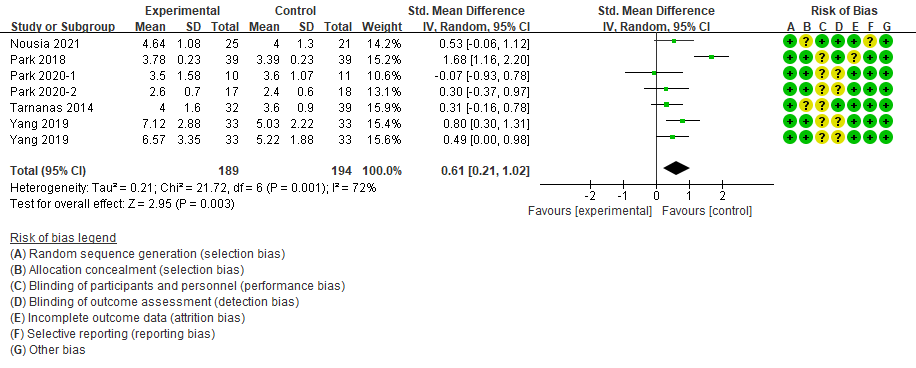


**A –** **DSB**


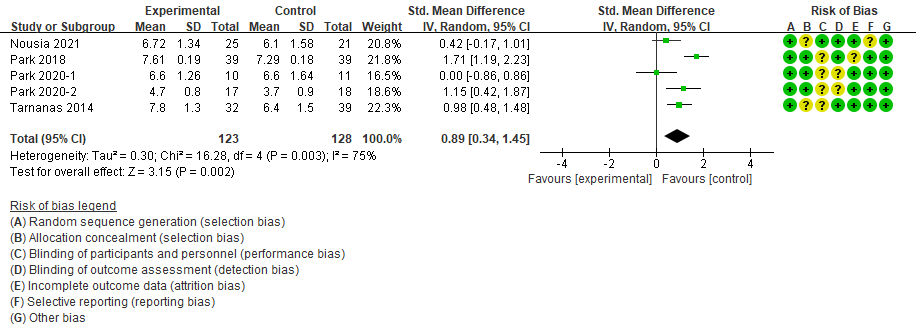


**B –** **DSF**

**Supplementary Figure 7.** Forest plot of attention.

**
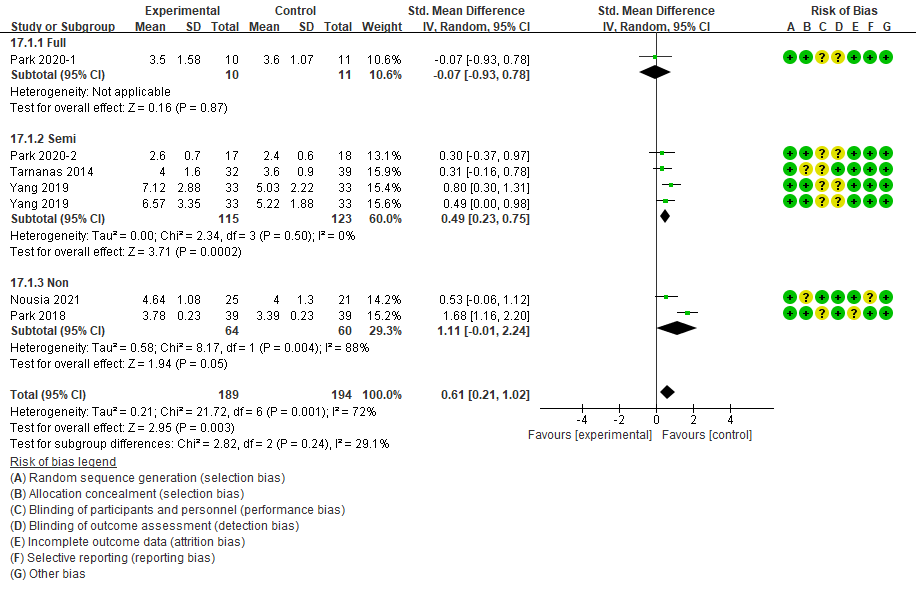
**

**A – Subgroup analysis by Immersion Level**

**
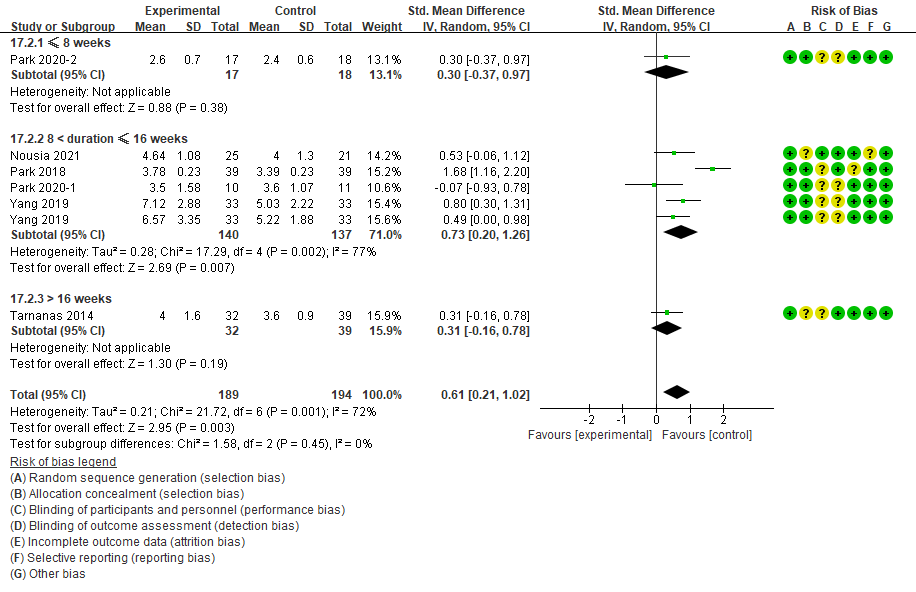
**

**B – Subgroup analysis by Duration**

**
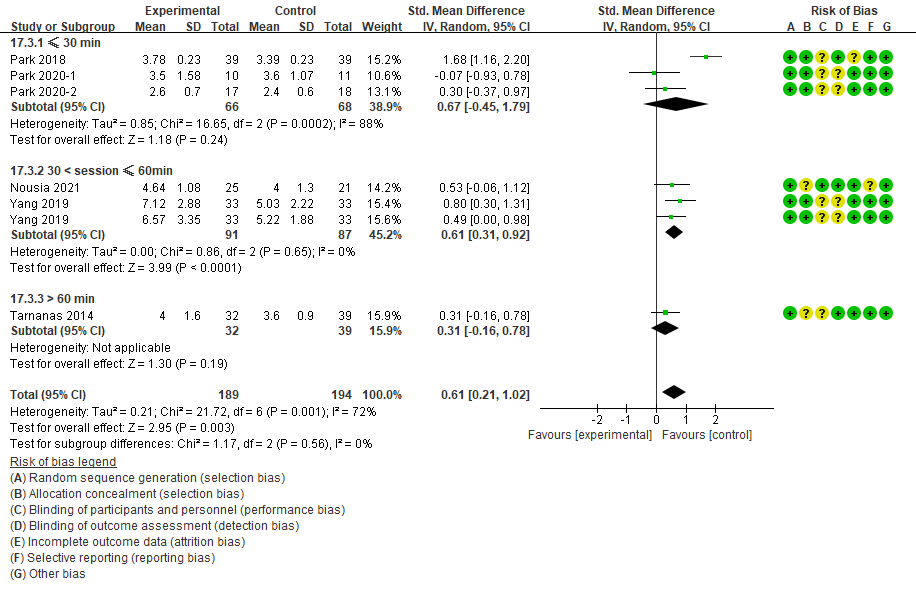
**

**C – Subgroup analysis by Session**

**
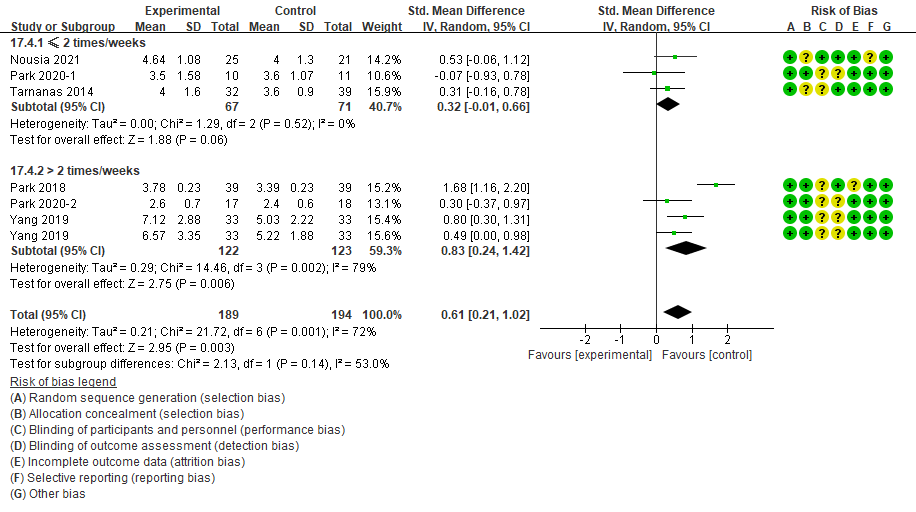
**

**D – Subgroup analysis by Frequency**

**
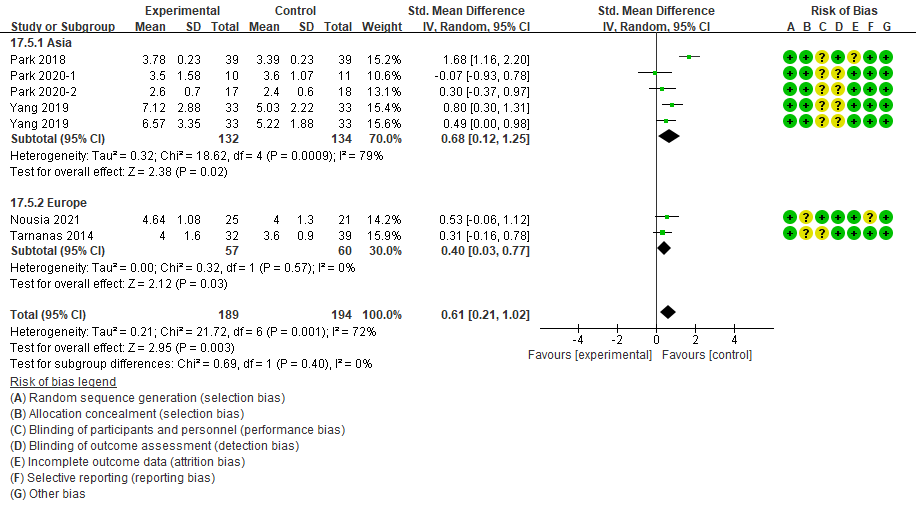
**

**E – Subgroup analysis by Geographic Region**

**
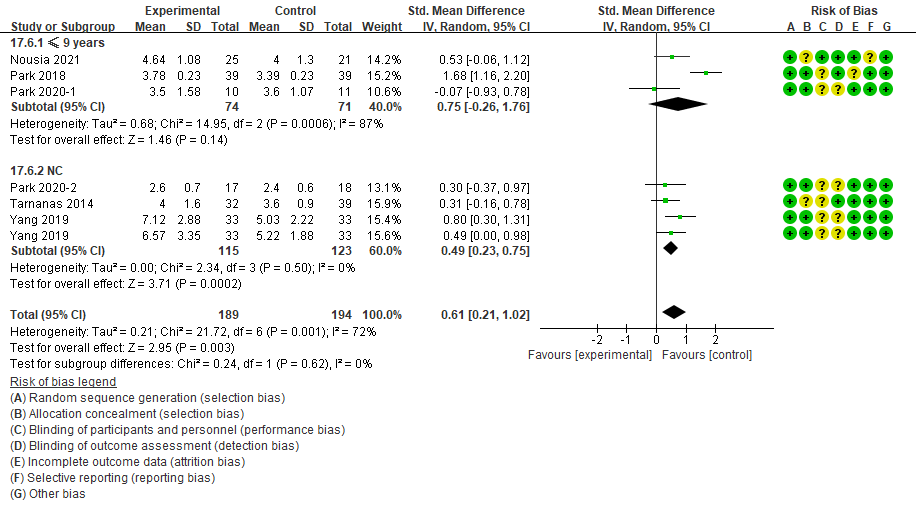
**

**F – Subgroup analysis by Education** **Level**

**
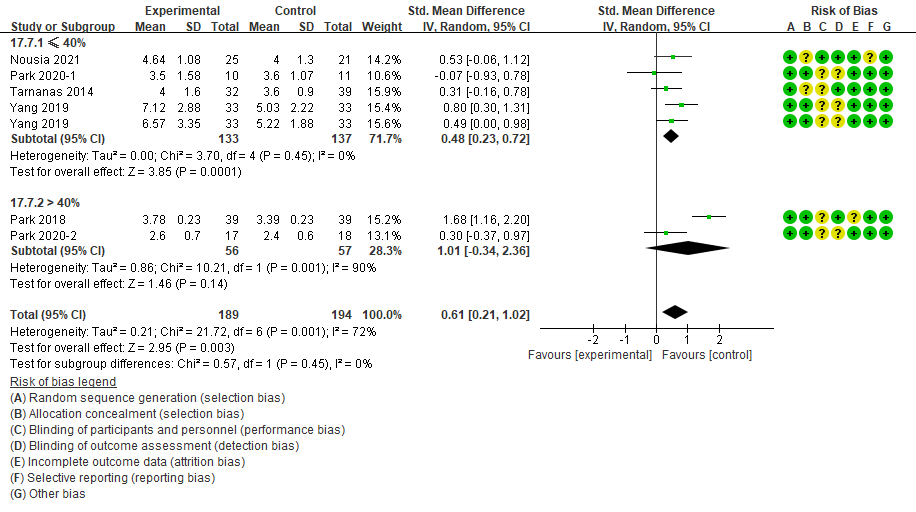
**

**G – Subgroup analysis by Male Proportion**

**Supplementary Figure 8.** Forest plot of subgroup analysis of DSB.

**
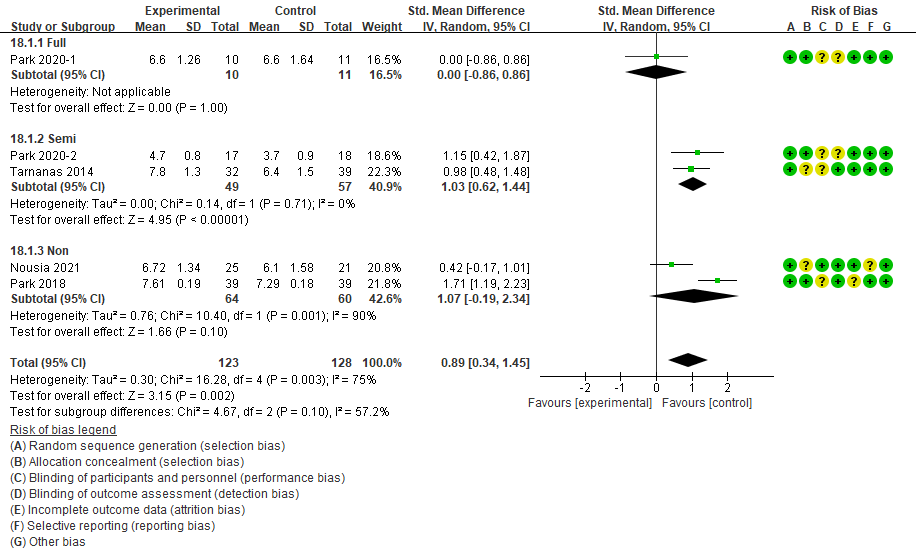
**

**A – Subgroup analysis by Immersion Level**

**
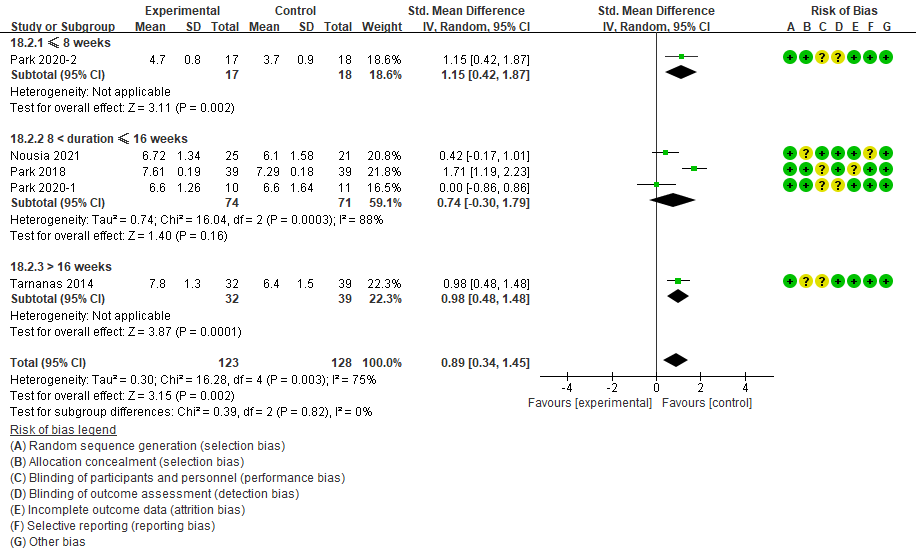
**

**B – Subgroup analysis by Duration**

**
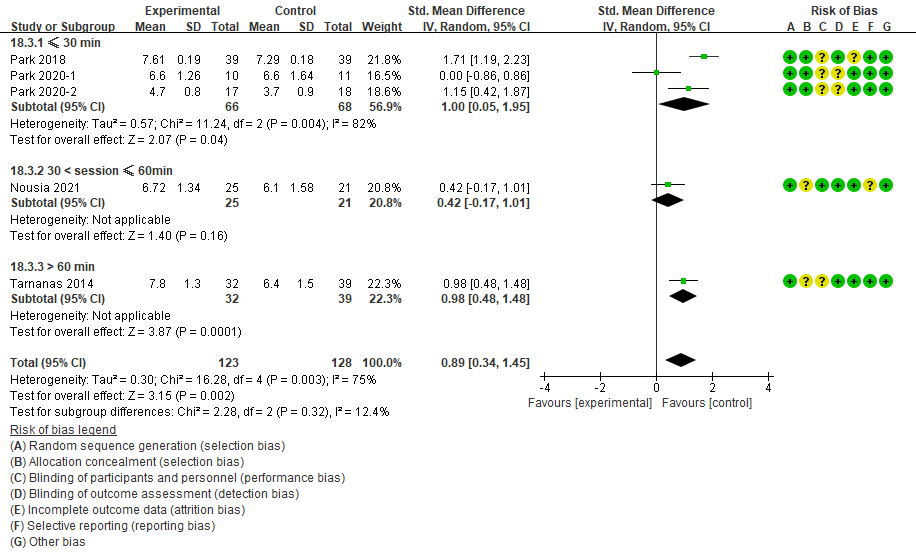
**

**C – Subgroup analysis by Session**

**
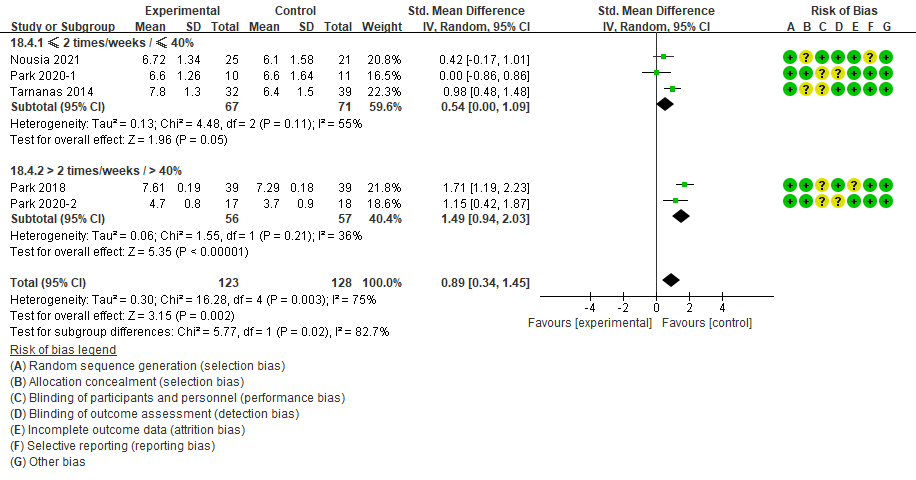
**

**D – Subgroup analysis by Frequency / Male Proportion**

**
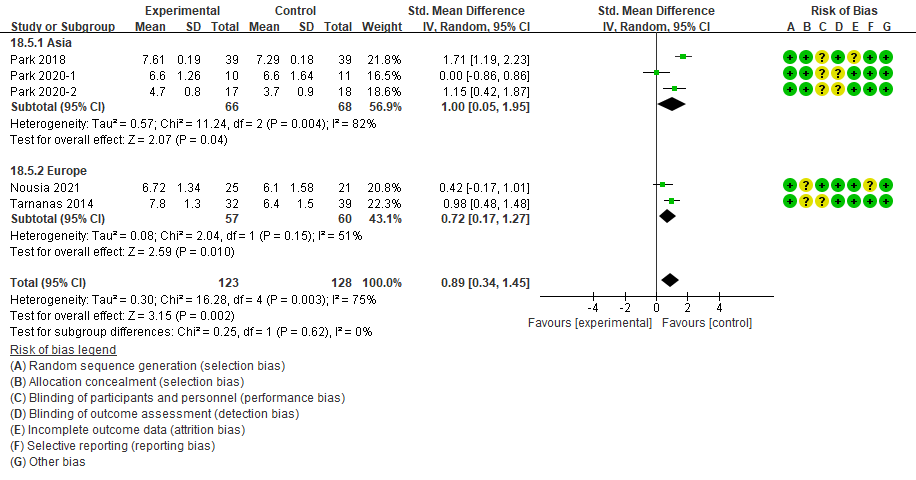
**

**E – Subgroup analysis by Geographic Region**

**
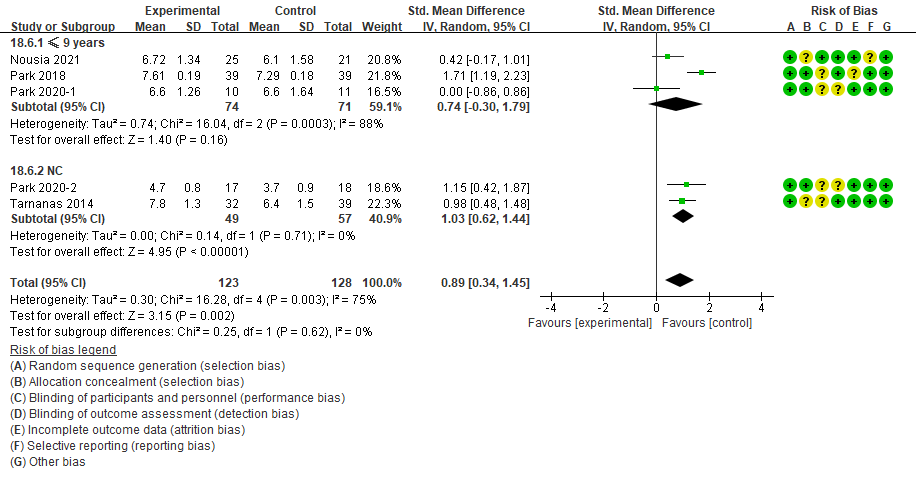
**

**F – Subgroup analysis by Education Level**

**Supplementary Figure 9.** Forest plot of subgroup analysis of DSF.


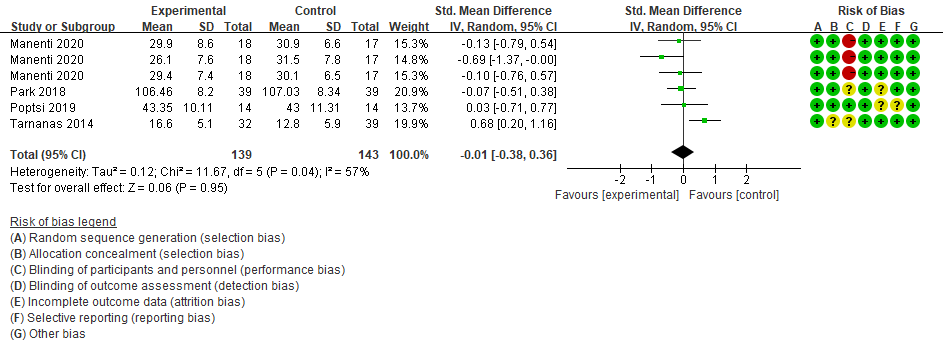


**A –** **RAVLT-IR**

**
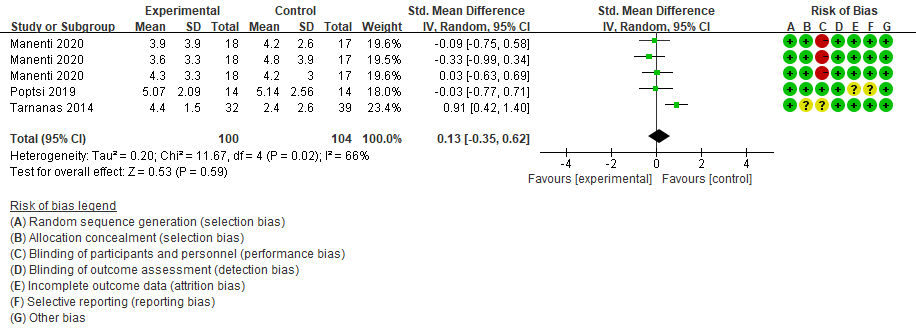
**

**B –** **RAVLT-DR**


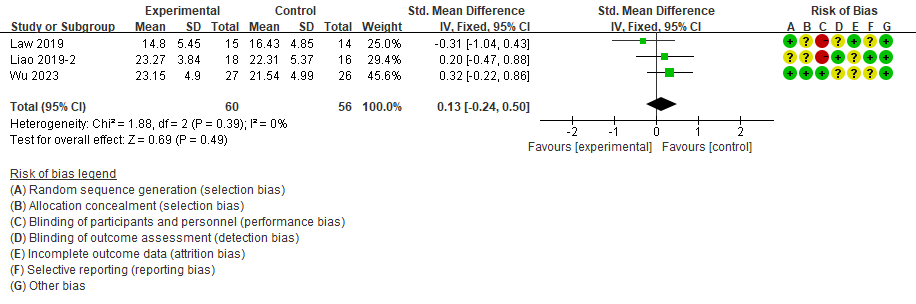


**C –** **CVVLT-IR**

**
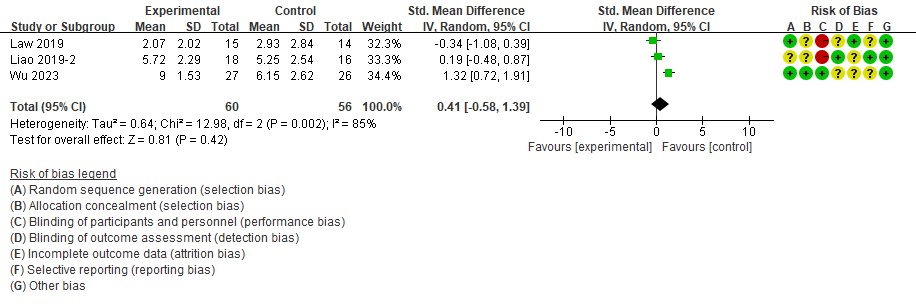
**

**D –** **CVVLT-DR**

**Supplementary Figure 10.** Forest plot of memory.

**
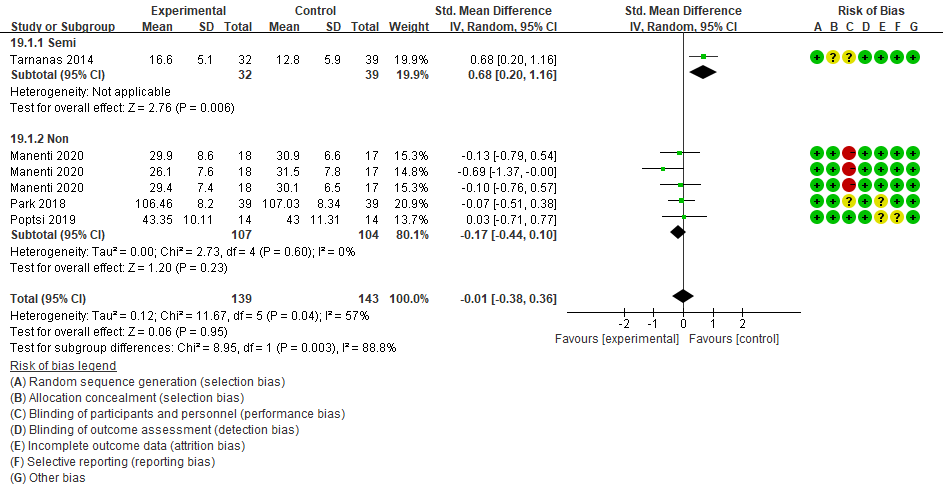
**

**A – Subgroup analysis by Immersion Level**

**
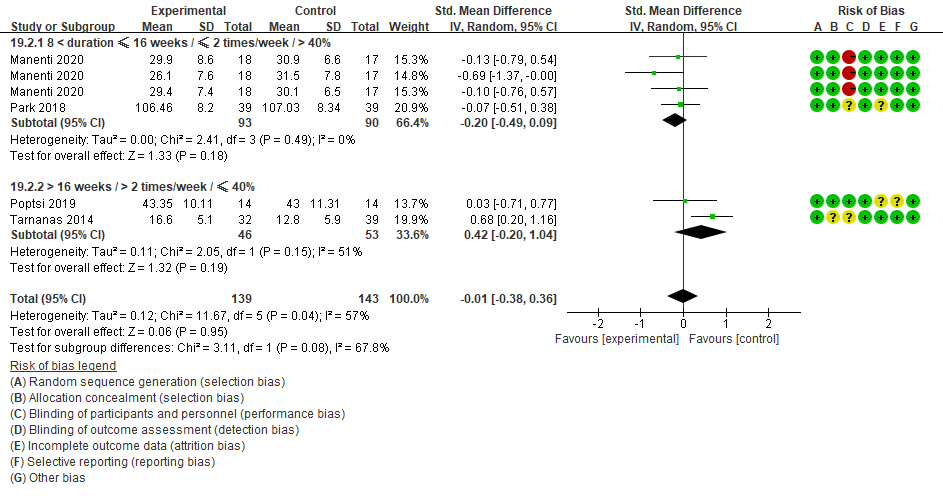
**

**B – Subgroup analysis by Duration / Frequency / Male Proportion**

**
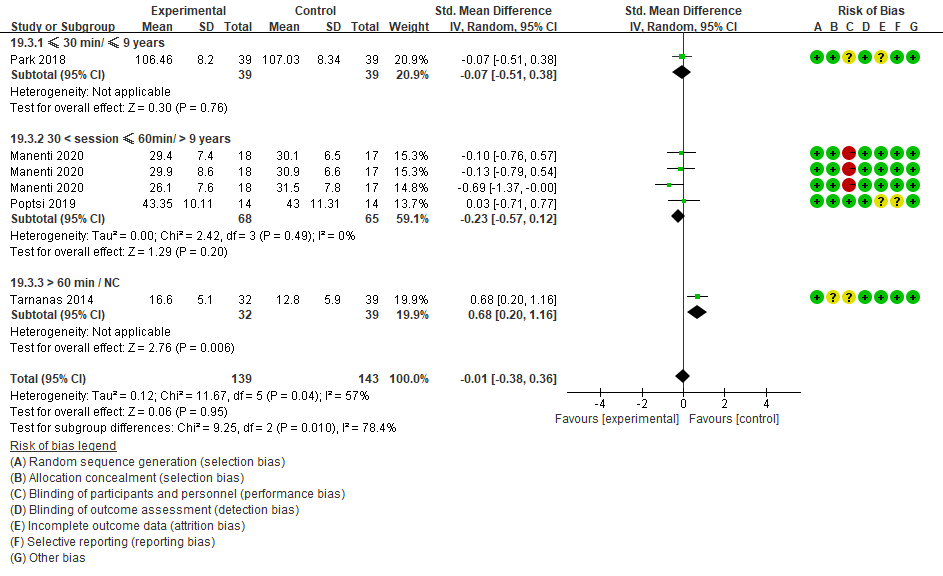
**

**C – Subgroup analysis by Session**

**
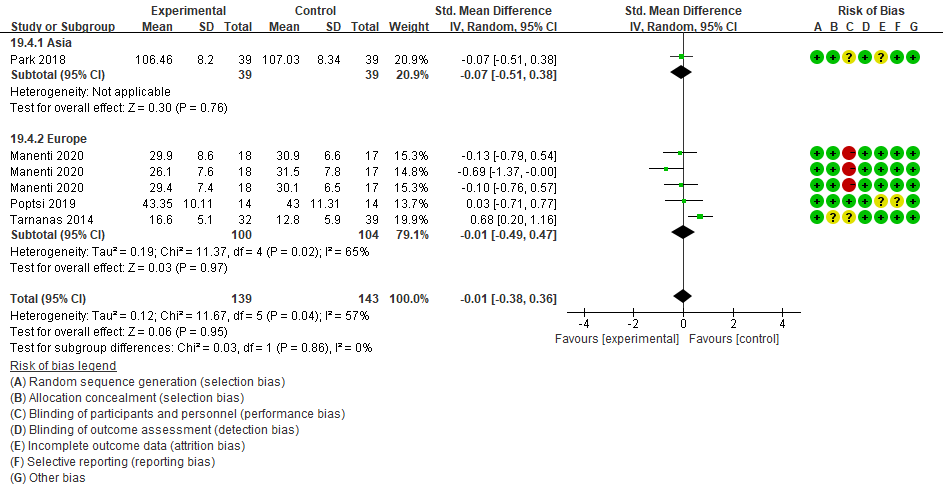
**

**E – Subgroup analysis by Geographic Region**

**Supplementary Figure 11.** Forest plot of subgroup analysis of RAVLT-IR.

**
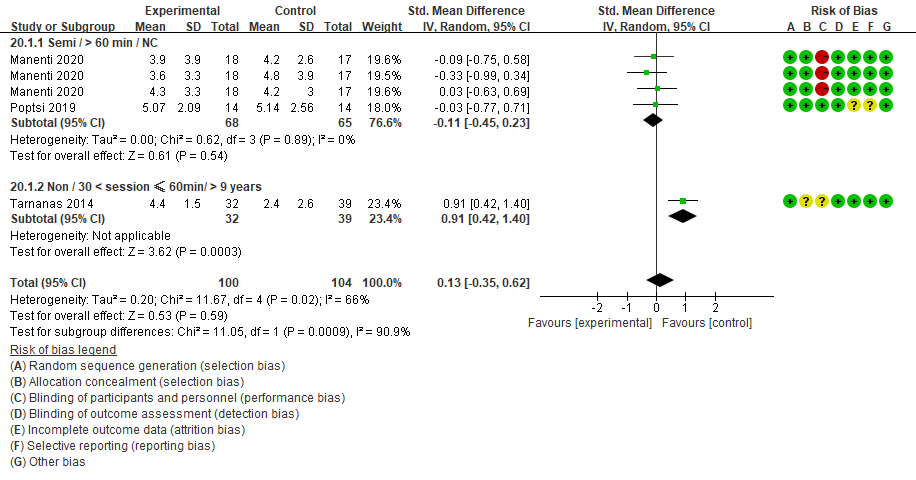
**

**A – Subgroup analysis by Immersion Level** **/ Session / Education Level**

**
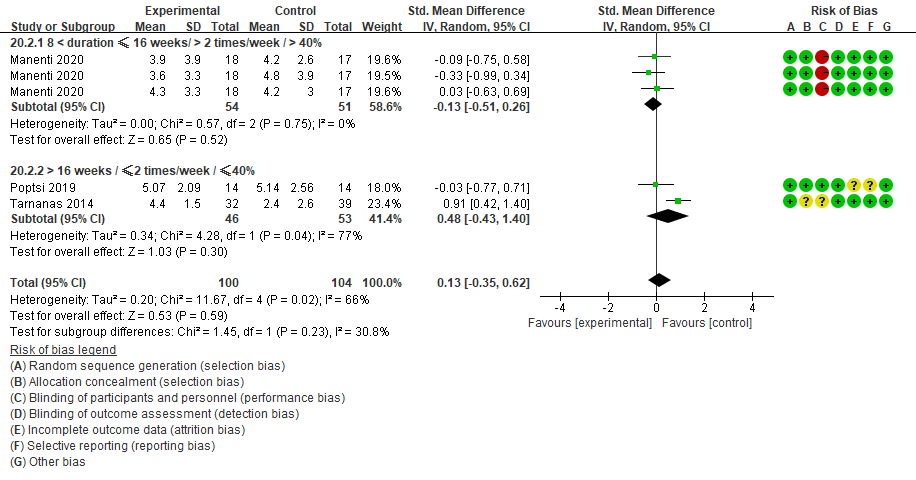
**

**B – Subgroup analysis by Duration / Frequency / Male Proportion**

**Supplementary Figure 12.** Forest plot of subgroup analysis of RAVLT-DR.

**
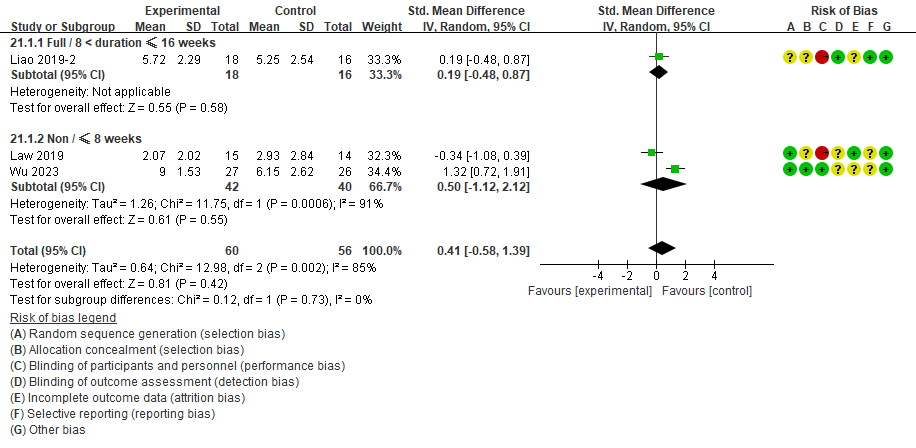
**

**A – Subgroup analysis by Immersion Level / Duration**

**
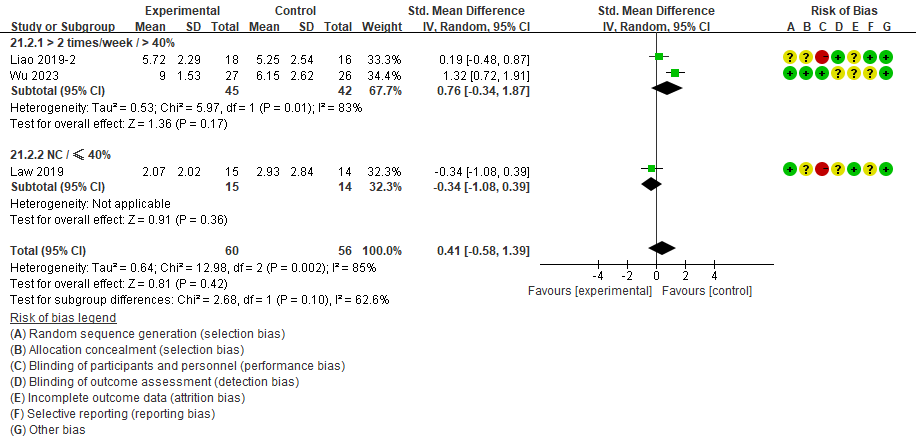
**

**B – Subgroup analysis by Frequency / Male Proportion**

**
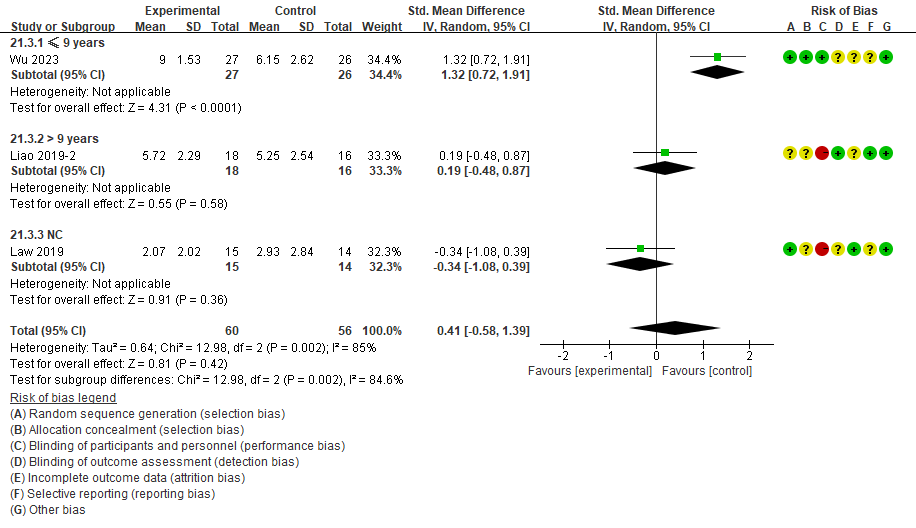
**

**C – Subgroup analysis by Education Level**

**Supplementary Figure 13.** Forest plot of subgroup analysis of CVVLT-DR.


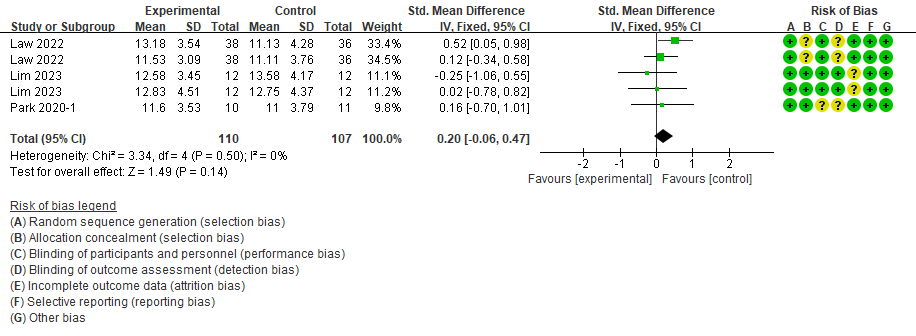


**A –** **Animal word**

**
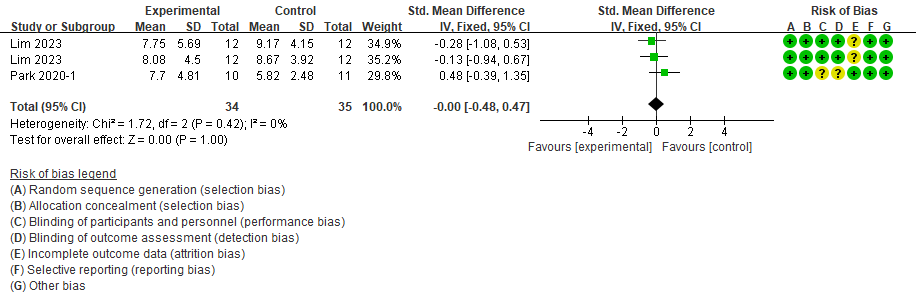
**

**B –** **“ㅅ” word**

**Supplementary Figure 14.** Forest plot of verbal fluency.


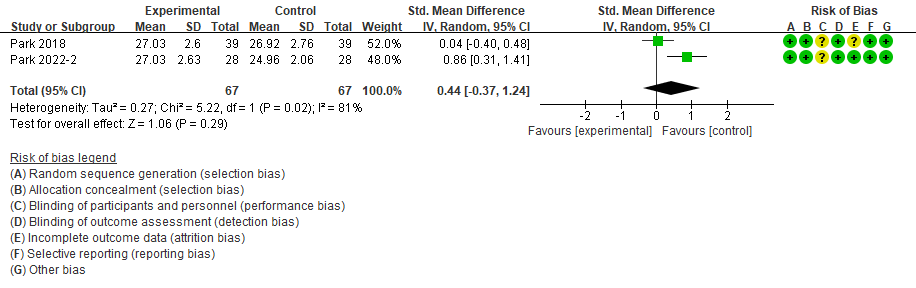


**A –** **WAIS-BDT**

**
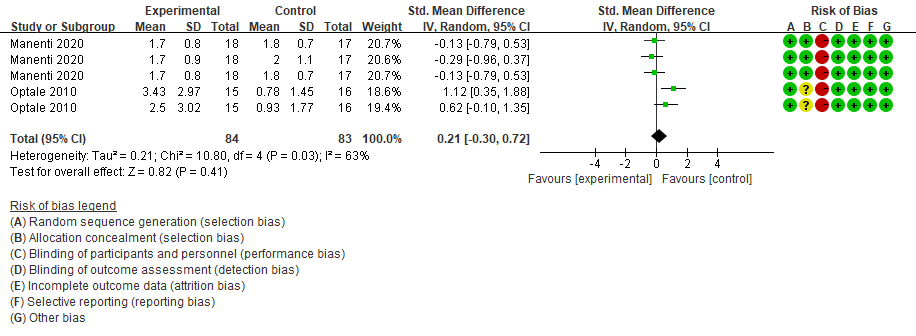
**

**B –** **CDT**

**Supplementary Figure 15.** Forest plot of visual ability.

**
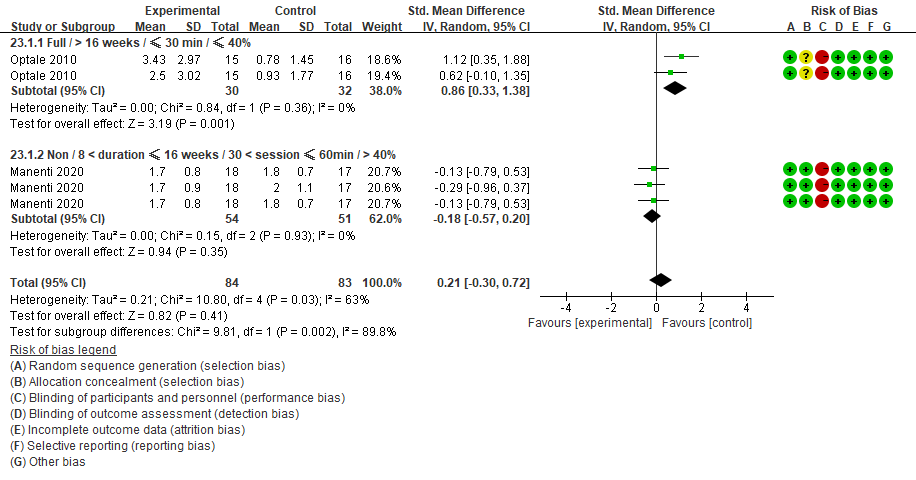
**

**A – Subgroup analysis by Immersion Level / Duration / Session / Male Proportion**

**Supplementary Figure 16.** Forest plot of subgroup analysis of CDT.


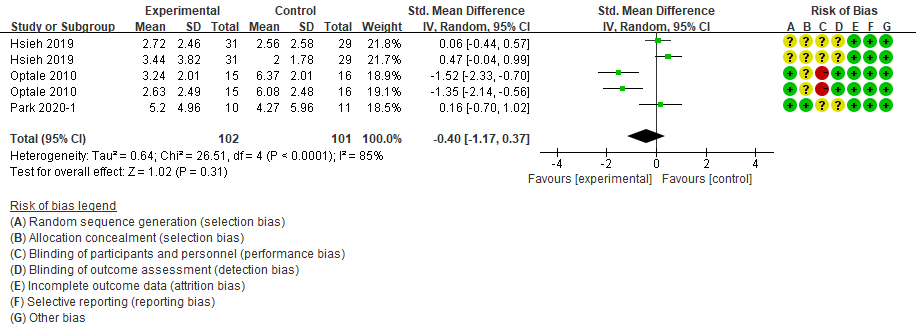


**A –** **GDS-15**

**
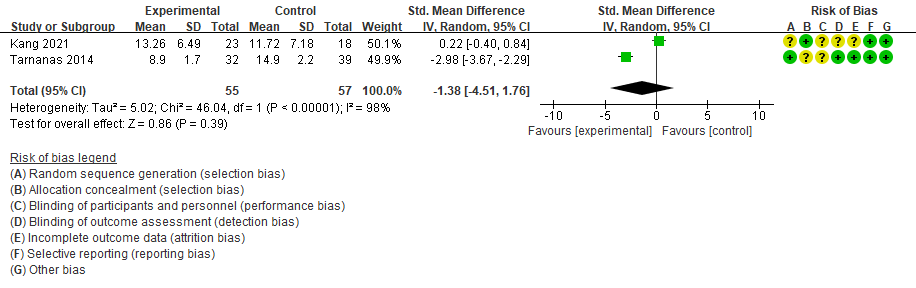
**

**B –** **GDS-30**

**Supplementary Figure 17.** Forest plot of emotional status.

**
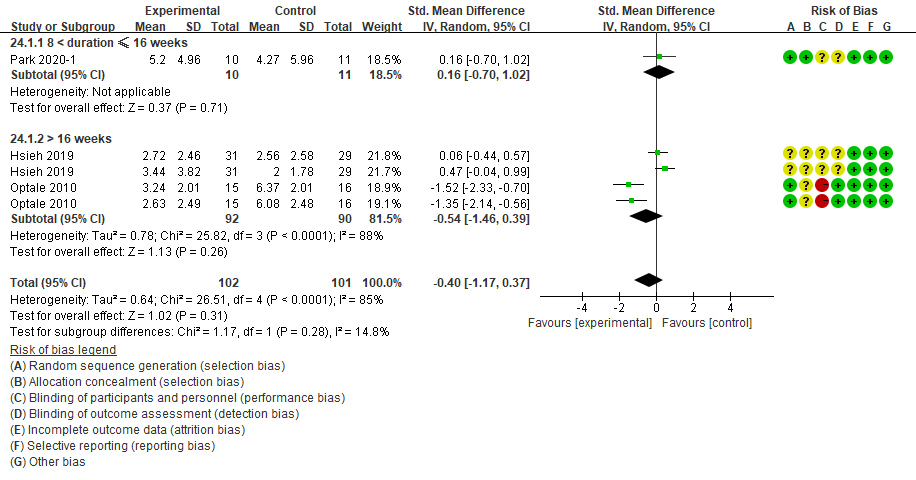
**

**A – Subgroup analysis by Duration**

**
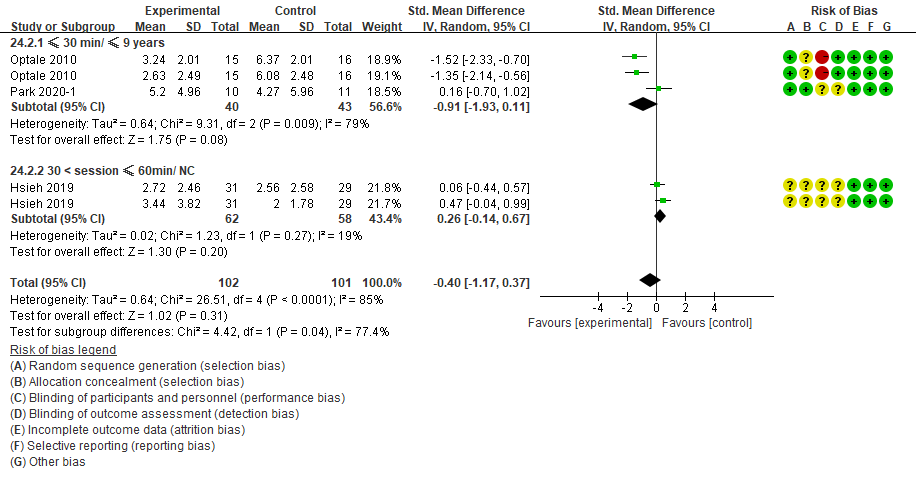
**

**B – Subgroup analysis by Session / Education Level**

**
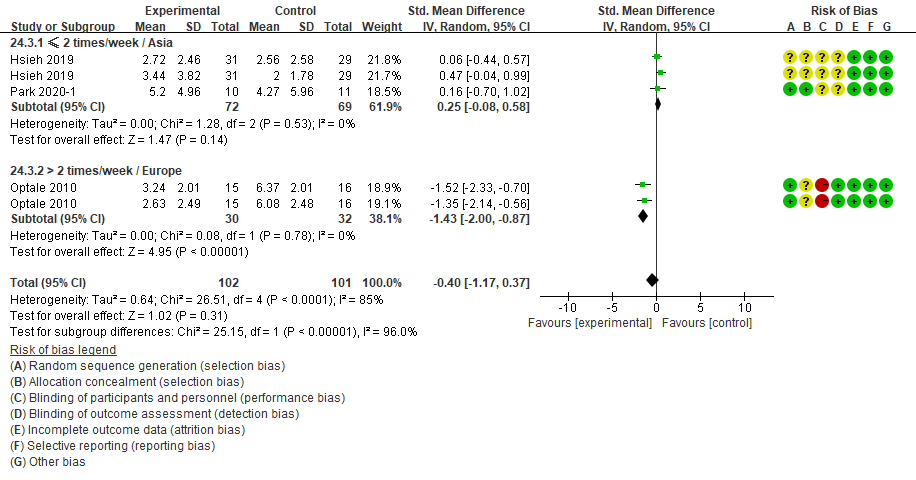
**

**C – Subgroup analysis by Frequency / Geographic Region**

**Supplementary Figure 18.** Forest plot of subgroup analysis of GDS-15.


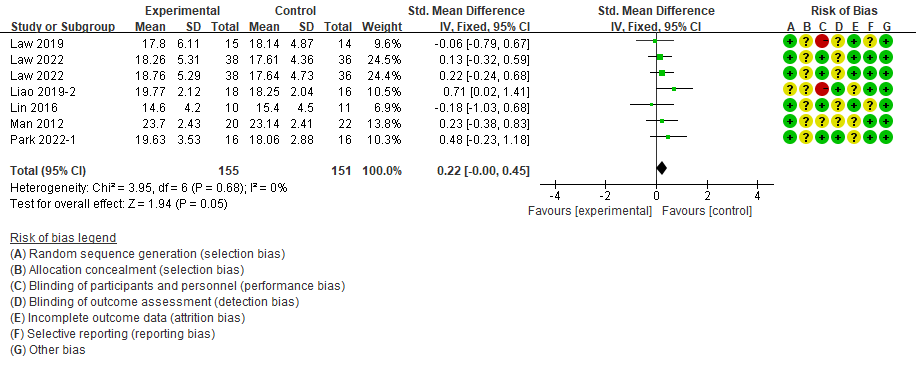


**A –** **IADL**

**
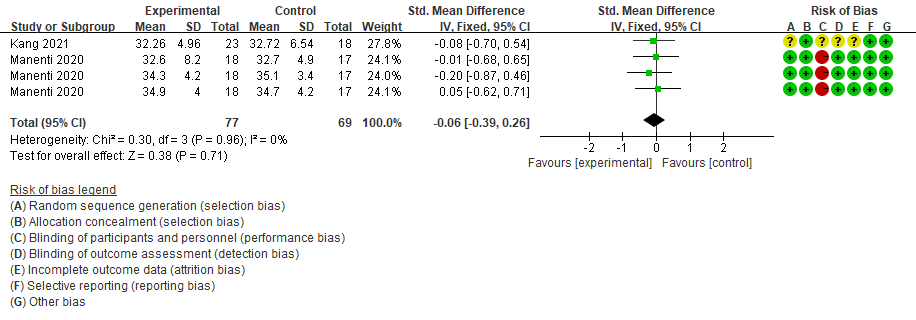
**

**B –** **QoL-AD**

**Supplementary Figure 19.** Forest plot of quality of life.


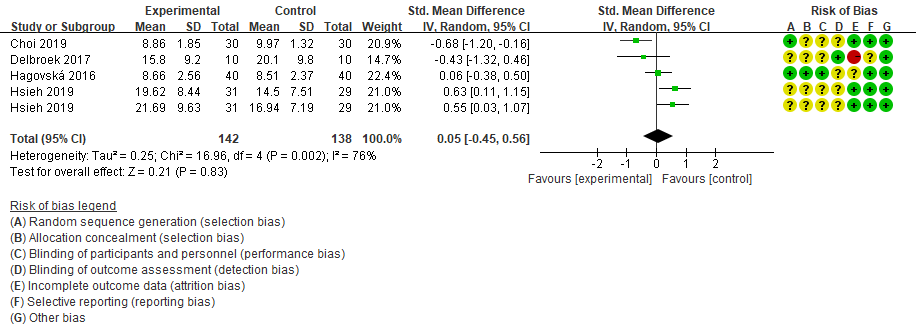


**A –** **TUG**

**
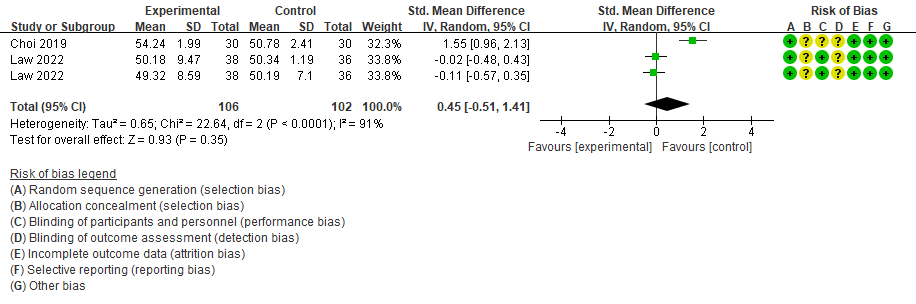
**

**B –** **BBS**

**Supplementary Figure 20.** Forest plot of dynamic balance.

**
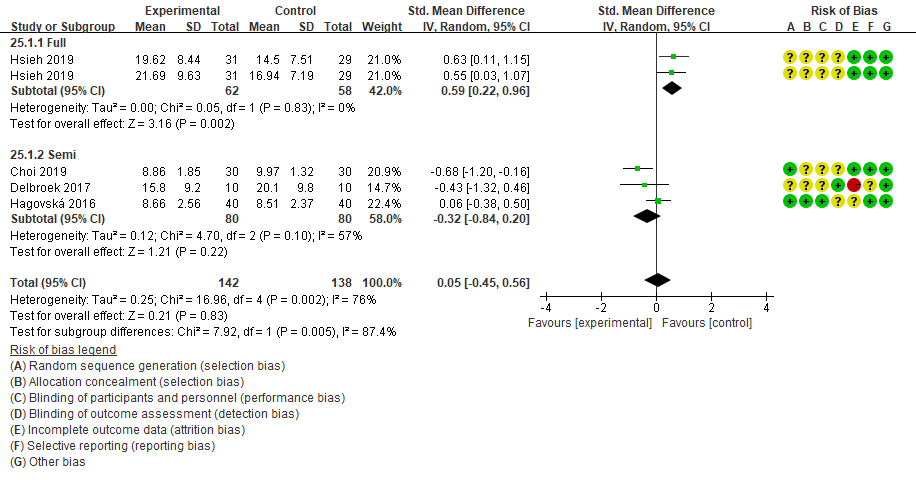
**

**A – Subgroup analysis by Immersion Level**

**
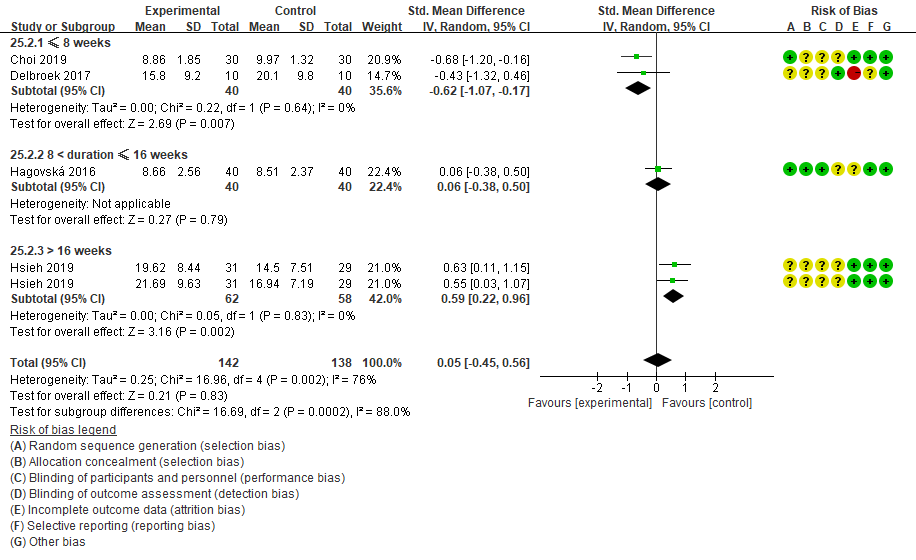
**

**B – Subgroup analysis by Duration**

**
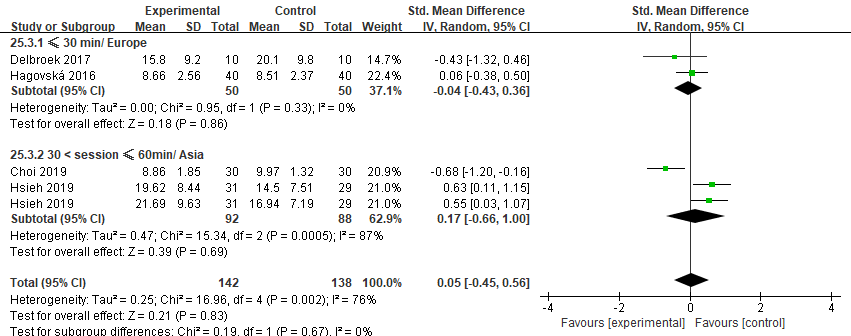
**

**C – Subgroup analysis by Session / Geographic Region**

**
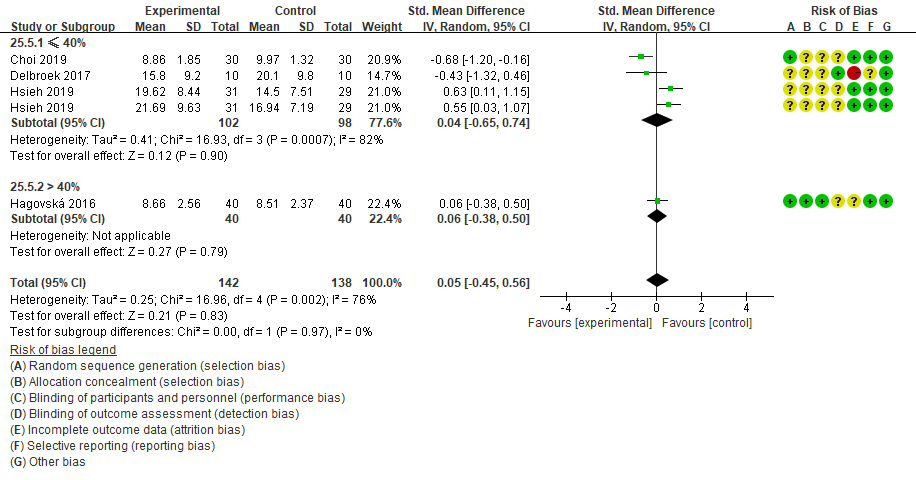
**

**D – Subgroup analysis by Male Proportion**

**Supplementary Figure 21.** Forest plot of subgroup analysis of TUG.


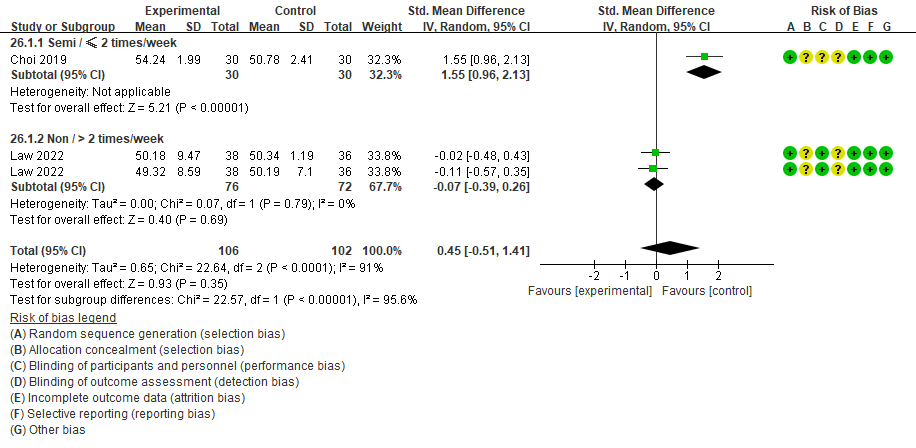


**A – Subgroup analysis by Immersion Level / Frequency**

**Supplementary Figure 22.** Forest plot of subgroup analysis of BBS.
